# Design of a double-decker coordination cage revisited to make new cages and exemplify ligand isomerism

**DOI:** 10.3762/bjoc.15.109

**Published:** 2019-05-21

**Authors:** Sagarika Samantray, Sreenivasulu Bandi, Dillip K Chand

**Affiliations:** 1 Department of Chemistry, Indian Institute of Technology Madras, Chennai 600036, India

**Keywords:** anion binding, double-decker cage, ligand isomerism, macrocycles, palladium, self-assembly, supramolecular

## Abstract

The complexation study of *cis*-protected and bare palladium(II) components with a new tridentate ligand, i.e., pyridine-3,5-diylbis(methylene) dinicotinate (**L1**) is the focus of this work. Complexation of *cis*-Pd(tmeda)(NO_3_)_2_ with **L1** at a 1:1 or 3:2 ratio produced [Pd(tmeda)(**L1**)](NO_3_)_2_ (**1a**). The reaction mixture obtained at 3:2 ratio upon prolonged heating, produced a small amount of [Pd_3_(tmeda)_3_(**L1**)_2_](NO_3_)_6_ (**2a**). Complexation of Pd(NO_3_)_2_ with **L1** at a 1:2 or 3:4 ratios afforded [Pd(**L1**)_2_](NO_3_)_2_ (**3a**) and [(NO_3_)_2_@Pd_3_(**L1**)_4_](NO_3_)_4_ (**4a**), respectively. The encapsulated NO_3_^–^ ions of **4a** undergo anion exchange with halides (F^–^, Cl^–^ and Br^–^ but not with I^–^) to form [(X)_2_@Pd_3_(**L1**)_4_](NO_3_)_4_ **5a–7a**. The coordination behaviour of ligand **L1** and some dynamic properties of these complexes are compared with a set of known complexes prepared using the regioisomeric ligand bis(pyridin-3-ylmethyl)pyridine-3,5-dicarboxylate (**L2**). Importantly, a ligand isomerism phenomenon is claimed by considering complexes prepared from **L1** and **L2**.

## Introduction

Coordination-driven self-assembly is a convenient strategy for the construction of supramolecules of desired dimensions via simple synthetic procedures. Well-defined metal–ligand coordination bonds enable the construction of designer targeted molecules with ease. The use of a palladium(II) component for complexation with a non-chelating bi- or polydentate ligand (usually *N*-donor ligands) is particularly advantageous for the construction of a variety of metallocages [1–5]. Complexation of *cis*-protected palladium(II), i.e., (PdL’) or bare palladium(II) with non-chelating bidentate ligands is known to afford a series of (PdL’)*_m_*L*_m_* or Pd*_m_*L_2_*_m_*-type self-assembled coordination complexes [5]. Pd_2_L_4_-type cages are the simplest representatives among the Pd*_m_*L_2_*_m_*-type complexes, yet most utilised [5–6]. The Pd_2_L_4_-type cages are well explored for the encapsulation of guests that are anionic [7–11], neutral [12–16], radical initiators [17], and drug molecules [18–19]. It is necessary to emphasize here that Pd_2_L_4_-type cages contain a cavity. McMorran and Steel reported the first Pd_2_L_4_-type cage and the anion binding ability of the cavity [20]. They also used a non-chelating tridentate ligand in an attempt to prepare a Pd_3_L_4_-type double-decker coordination cage that would possess two cavities, if formed. However, the plan could not be executed as one of the coordinating atoms of the ligand remained unutilized [21]. Instead of the desired Pd_3_L_4_ architecture, they observed a PdL_2_-type spirometallomacrocycle where bare palladium(II) is the juncture between two metallomacrocyclic rings. We report here a Pd_3_L_4_-type cage prepared from Pd(NO_3_)_2_ and pyridine-3,5-diylbis(methylene) dinicotinate (**L1**)_._ We reported earlier the first Pd_3_L_4_-type double-decker coordination cage using a simple tridentate “E” shaped ester-based ligand bis(pyridin-3-ylmethyl)pyridine-3,5-dicarboxylate (**L2**) [22–23]. An additional feature in our design using **L2** is the stoichiometrically controlled formation of PdL_2_-type spiro and Pd_3_L_4_-type double-decker complexes that is reversible under appropriate conditions. Subsequently, other research groups (Chand, Clever, Crowley and Yoshizawa groups) published Pd_3_L_4_-type cages [24]. This design has been further explored by Crowley et al. for the synthesis of a Pd_4_L_4_-type triple-decker cage [25]. The Clever research group reported a system in which two units of a double-decker cage are interlocked [26]. In this context, we revisited our earlier design of Pd_3_L_4_-type cages to prepare a corresponding analogues cages using the new ligand **L1** (that is a positional isomer or regioisomer of **L2**) in order to exemplify ligand isomerism.

Ligand isomerism includes metal complexes (at least two) having the same molecular formula but are composed of different positional isomers (regioisomers) of the ligand. Positional isomers (regioisomers) of a non-chelating ligand system capable of forming palladium(II) complexes of same molecular formula is a rare phenomenon [27–34]. Such palladium(II) complexes should represent the phenomenon of “ligand isomerism”. We reported a family of Pd_2_L_4_-type complexes that fits under the definition of ligand isomerism [34]. In the pursuit of ligand isomerism in Pd_3_L_4_-type double-decker cages we intended to include our reported cage (prepared from palladium(II) and **L2**) [22–23] and construct a new isomeric Pd_3_L_4_-type complex. The complexation study of *cis*-protected and bare palladium(II) components with the new tridentate ligand, i.e., pyridine-3,5-diylbis(methylene) dinicotinate (**L1**) is the focus of this work. In addition, the dynamic behavior as well as anion binding abilities of selected complexes are also probed. Ligand **L1** is a constitutional isomer of ligand **L2** [22–23] and is expected to exhibit similarities but also some differences in complexation behavior with palladium(II) components. There are also some similarities and some differences in the related properties of these complexes.

## Results and Discussion

### Design and synthesis of ligand **L1**

**Figure 1:**
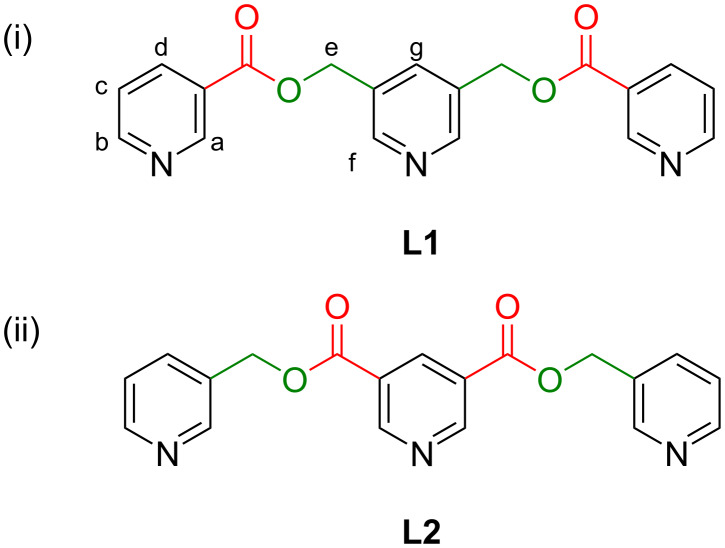
The ligands (i) **L1** and (ii) **L2** that are positional isomers (regioisomers).

The new ligand **L1** was designed as a positional isomer (regioisomer) of the known ligand **L2** (Figure 1). Each of these ligands has three pyridine moieties separated by two spacer moieties (–CH_2_OC(=O)–). Both ligands are semi-rigid/semi-flexible due to the spacers’ conformational mobility. The “E-shaped” conformation of the ligand that is suitable for the formation of the targeted Pd_3_L_4_-type complex is shown here for clarity of discussion. In a given ligand, two of the pyridine rings are substituted in the 3-position and are terminal and symmetrically disposed with respect to the central/internal 3,5-disubstituted pyridine ring. The spacers are identical in both ligands, however, their orientations are reversed in ligand **L1** as compared to the known ligand **L2**. The primary intention of the design of **L1** was to have a positional isomer (regioisomer) of the ligand **L2**.

The tridentate ligand pyridine-3,5-diylbis(methylene) dinicotinate (**L1**, Figure 1) was prepared by condensation of pyridine-3,5-diyldimethanol [35] with nicotinoyl chloride hydrochloride in dry dichloromethane in the presence of triethylamine. The reaction mixture was stirred at room temperature for 24 h followed by aqueous work-up and column chromatography purification to afford pyridine-3,5-diylbis(methylene) dinicotinate (**L1**) as a white solid. The ligand was fully characterized by NMR spectroscopy and ESIMS techniques (Supporting Information File 1, Figures S1–S11). In addition, NOESY analysis was helpful in distinguishing the protons H_a_ and H_f_.

It is assumed that the electron density at the central pyridine ring in **L1** (that is a lutidine derivative) should be higher than that at the central pyridine ring of **L2** (that is a dinicotinate derivative) having electron-withdrawing carbonyl substituents. Also, the electron density at the terminal pyridine ring in **L1** (that is a nicotinate derivative) should be lower than that at the terminal pyridine ring of **L2** (that is a picolyl derivative). The electrostatic potential maps at the terminal and internal pyridine nitrogen calculated using DFT methods (Supporting Information File 1, Table S2), however, are found to be comparable. Nevertheless, it seemed interesting to check whether or not the subtle difference in the electron density at the pyridine N centers has any influence on the coordination behavior of the ligands.

### Complexation of palladium(II) components with ligand **L1**

**Scheme 1:**
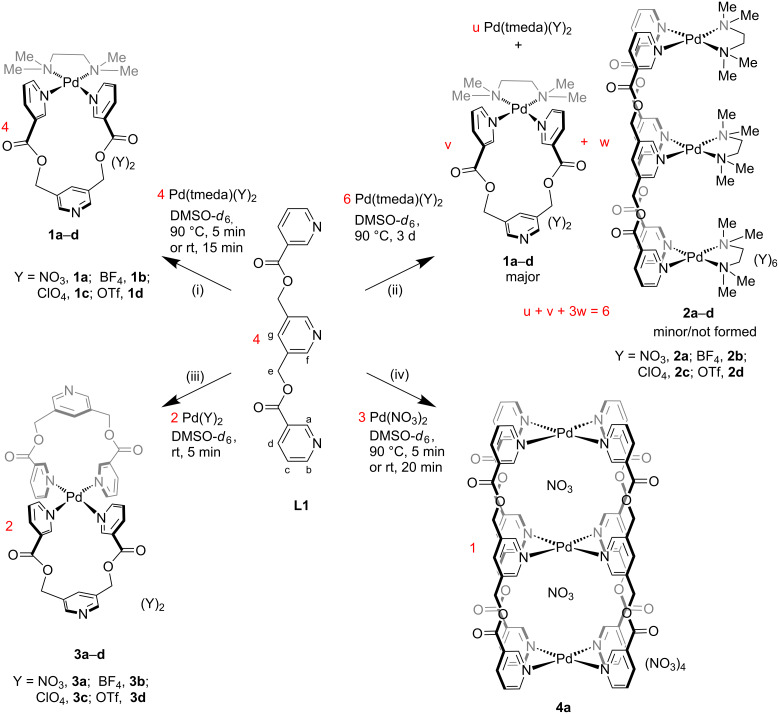
(i)/(ii) Complexation of Pd(tmeda)(Y)_2_ with the ligand **L1** at 1:1 and 2:3 metal-to-ligand ratios, respectively; (iii)/(iv) complexation of Pd(Y)_2_ with the ligand **L1** at 1:2 and 3:4 metal-to-ligand ratios, respectively. For (i)/(ii)/(iii): Y = NO_3_^−^, BF_4_^−^, ClO_4_^−^, or OTf^−^ (**2b** was not formed).

Complexation of *cis*-protected palladium(II) was carried out with the ligand **L1** at two different metal-to-ligand ratios (1:1 and 3:2). We also carried out the complexation of bare palladium(II) with the ligand **L1** at two different metal-to-ligand ratios (1:2 and 3:4). The complexation reactions performed in DMSO-*d*_6_ allowed the monitoring of complex formation and those performed in DMSO were used for isolation of the complex by precipitation methods. The resulting complexes at specified ratios of the reactants are depicted in Scheme 1 and the details of the complexation behavior are described below.

### Complexation of *cis*-protected palladium(II) with ligand **L1** at 1:1 metal-to-ligand ratio

**Figure 2:**
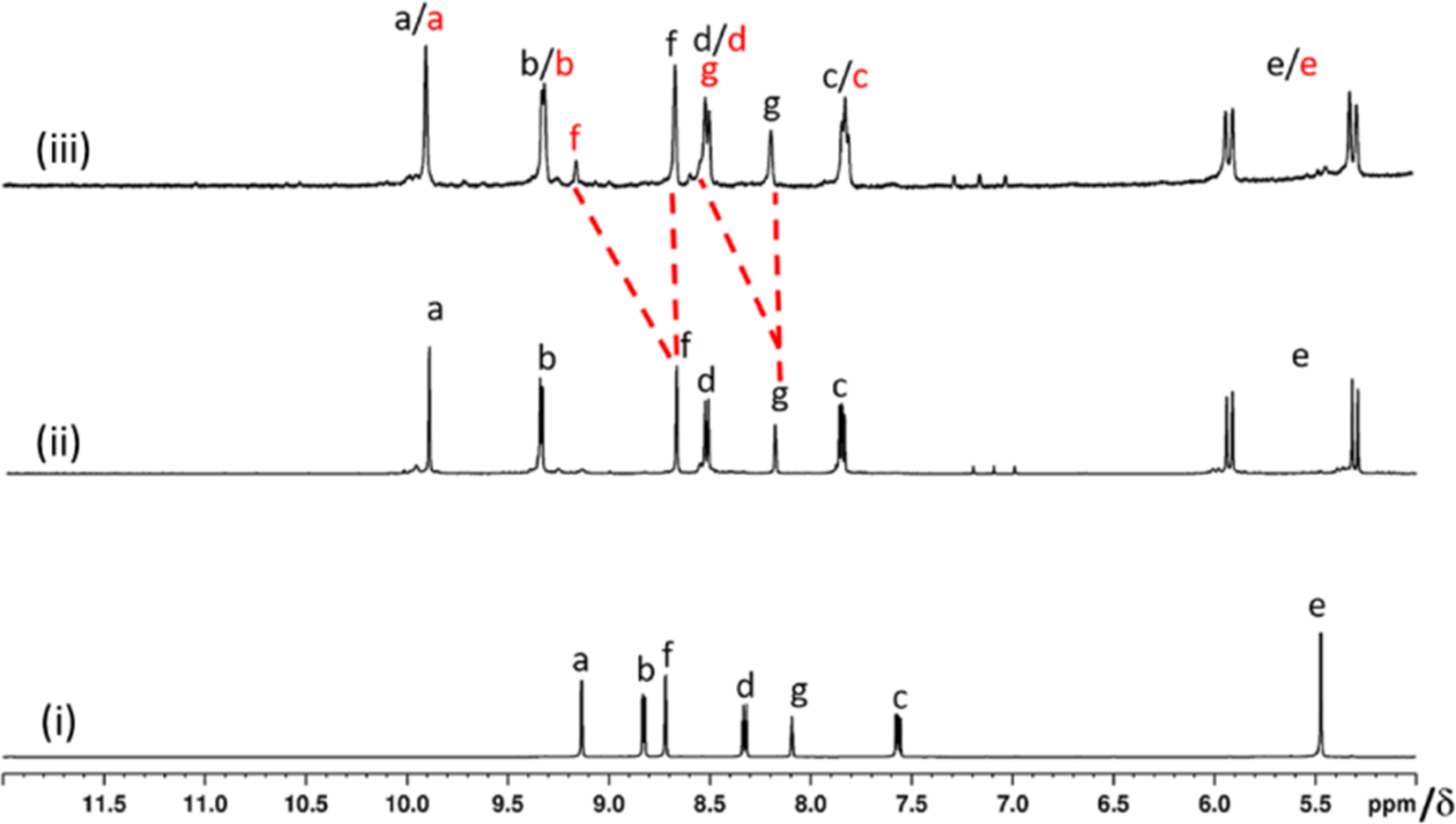
Partial ^1^H NMR spectra in DMSO-*d*_6_ for (i) **L1**, (ii) [Pd(tmeda)(**L1**)](NO_3_)_2_ (**1a**) and (iii) a mixture of [Pd(tmeda)(**L1**)](NO_3_)_2_ (**1a**) and [Pd_3_(tmeda)_3_(**L1**)_2_](NO_3_)_6_ (**2a**).

The addition of one equivalent of *cis*-Pd(tmeda)(NO_3_)_2_ to a clear solution of one equivalent of the ligand **L1** in DMSO-*d*_6_ produced a turbid mixture. However, a clear yellow solution was obtained upon stirring the mixture at 90 °C for 5 min or at rt for 15 min. The reaction was repeated in DMSO and the (PdL’)L-type complex [Pd(tmeda)(**L1**)](NO_3_)_2_ (**1a**, Scheme 1(i)) was isolated from the reaction mixture by a precipitation method that is described in the experimental section. The ^1^H NMR spectrum of the solution showed formation of a single discrete complex (Figure 2(ii)). Counter-anion (BF_4_^−^, ClO_4_^−^ and OTf^−^) variation was also carried out to successfully prepare a series of complexes [Pd(tmeda)(**L1**)](BF_4_)_2_ (**1b**); [Pd(tmeda)(**L1**)](ClO_4_)_2_ (**1c**); [Pd(tmeda)(**L1**)](OTf)_2_ (**1d**). The complexes **1a–d** were prepared by mixing the corresponding metal component Pd(tmeda)(Y)_2_ with the ligand **L1** where Y = NO_3_^−^, BF_4_^−^, ClO_4_^−^ and OTf^−^. The metal components were prepared in situ by reacting Pd(tmeda)(Cl)_2_ with AgY in DMSO-*d*_6_ followed by separation of the precipitated AgCl.

The complex **1a** was characterized by various NMR techniques (Supporting Information File 1, Figures S12–S16). The ^1^H NMR spectrum of compound **1a** showed single set of peaks (Figure 2(ii)) characterized by complexation-induced downfield shifts of protons belonging to the terminal pyridines (Δδ = 0.77, 0.52, and 0.29 ppm for H_a_, H_b_, and H_c_, respectively) as compared to the free ligand **L1**. The peak positions of H_f_ and H_g_ remained unchanged which indicated that the central pyridine ring is not involved in the complexation. The ^1^H NMR spectra of compounds **1b**, **1c** and **1d** are very much comparable to that of **1a** (Supporting Information File 1, Figure S17). One of the coordination sites of the ligand **L1** thus remained unutilized in these mononuclear complexes.

The ESIMS data of the compounds **1b**, **1c** and **1d** confirmed the formation of mononuclear complexes (Supporting Information File 1, Figures S18–S20). As an example, the ESIMS spectrum of compound **1b** (Supporting Information File 1, Figure S18) showed isotopic peak patterns at *m*/*z* 658.14 and 285.57, respectively, which correspond to the cationic fragments [**1b** − BF_4_]^+^ and [**1b** − 2BF_4_]^2+^ that are formed due to the loss of one and two units of counter anions from **1b**. The experimental and theoretical peak patterns were found to be in agreement. The data of **1c** and **1d** are given in Supporting Information File 1.

### Complexation of *cis*-protected palladium(II) with ligand **L1** at a 3:2 metal-to-ligand ratio

The addition of three equivalents of *cis*-Pd(tmeda)(NO_3_)_2_ to a clear solution of two equivalents of ligand **L1** in DMSO-*d*_6_ produced a turbid mixture. However, a clear yellow solution was obtained upon stirring the mixture at 90 °C for 5 min. The progress of the complexation reaction was monitored by ^1^H NMR spectroscopy. We targeted a Pd_3_L’_3_L_2_-type complex [36], i.e., [Pd_3_(tmeda)_3_(**L1**)_2_](NO_3_)_6_ (**2a**, Scheme 1(ii)). However, the NMR spectrum showed the formation of [Pd(tmeda)(**L1**)](NO_3_)_2_ (**1a**) and uncomplexed *cis*-Pd(tmeda)^2+^. The reaction was allowed to continue where upon the integration ratios of the peaks corresponding to H_f_ and H_g_ were lower than expected and that of H_d_ was higher than expected. In addition, a new peak was observed at around 9.15 ppm (Figure 2(iii)). Careful analysis of the data led us to propose the formation of a minor proportion of **2a** along with a major proportion of **1a** and unutilized *cis*-Pd(tmeda)^2+^ remaining in solution. The peaks assigned to H_f_ and H_g_ of **2a** are shifted downfield compared to those of H_f_ and H_g_ in **1a** whereas other signals of **2a** merged with the respective signals of **1a**. The observed downfield shift of the H_f_ signal in **2a** is complexation-induced and found to have a unique position. The downfield shift of the H_g_ signal in **2a** could not be induced by complexation and is best described by considering an anisotropy effect of the nearby carbonyl groups the ligand strand. The H_g_ signal of **2a**, however, merged with the H_d_ signal of **1a**.

Subsequently, anion variation (BF_4_^−^, ClO_4_^−^ and OTf^−^) was carried out in anticipation of the trinuclear products **2b–d** (Scheme 1). Complexation of the metal component Pd(tmeda)(Y)_2_ with the ligand **L1** were carried out where Y = NO_3_^−^, BF_4_^−^, ClO_4_^−^ and OTf^−^. The metal components were prepared in situ by reacting Pd(tmeda)(Cl)_2_ with AgY in DMSO-*d*_6_ followed by separation of the precipitated AgCl. The ^1^H NMR spectra of the samples confirmed the formation of mononuclear complexes **1b–d** at initial stages (Supporting Information File 1, Figures S22–S24). All reactions were allowed to continue and their progress was monitored by ^1^H NMR spectroscopy. The peak positions in the ^1^H NMR spectrum of the sample containing BF_4_^−^ as counter anion remained unchanged but the same for the samples containing ClO_4_^−^ and OTf^−^ behaved in a manner very similar to the case of NO_3_^−^. A new peak was observed at around 9.10 ppm corresponding to H_f_ in each case. When integration ratio of H_a_ is taken as 1.0 the integration ratio of H_f_ for **1d** and **2d** in the mixture were found to be ≈0.8 and ≈0.2, respectively. It may be noted here that a comparable trinuclear complex of ligand **L2**, however, did not form [23].

ESIMS data were collected in anticipation of detecting the trinuclear complexes (Supporting Information File 1, Figures S25 and S26). Isotopic peak patterns at *m*/*z* 391.55 corresponding to the fragment [**2c** − 4ClO_4_]^4+^ confirmed the existence of the trinuclear complex **2c**. Similarly, isotopic peak patterns at *m*/*z* 2111.08 corresponding to the fragment [**2d** − OTf]^+^ confirmed the existence of trinuclear complex **2d**. The experimental and theoretical patterns were found to be in agreement.

### Complexation of bare palladium(II) with ligand **L1** at a 1:2 metal-to-ligand ratio

**Figure 3:**
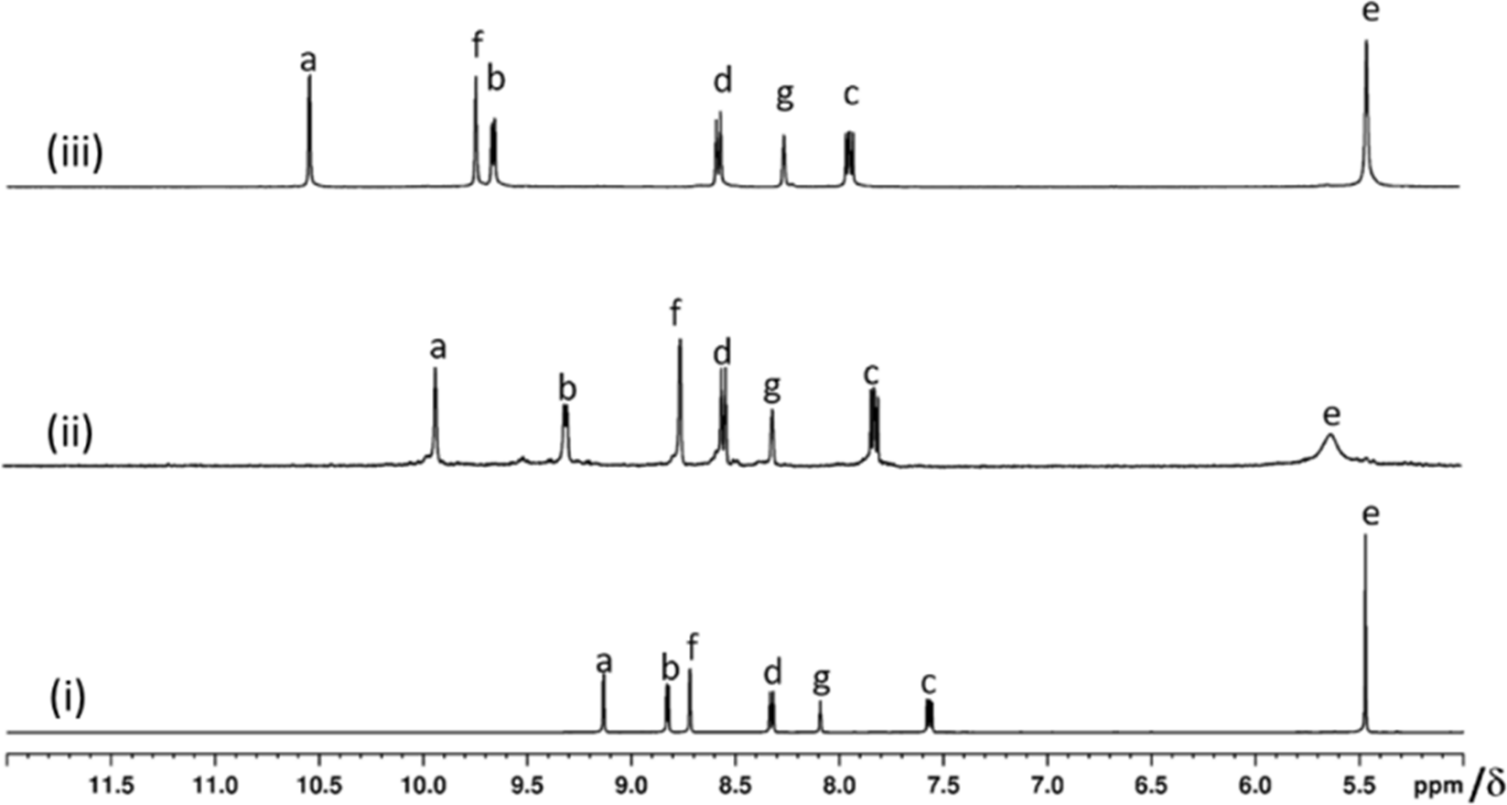
Partial ^1^H NMR spectra in DMSO-*d*_6_ for (i) **L1**, (ii) [Pd(**L1**)_2_](NO_3_)_2_ (**3a**) and (iii) [(NO_3_)_2_@Pd_3_(**L1**)_4_](NO_3_)_4_ (**4a**).

The sample of Pd(NO_3_)_2_ used in this work was commercially acquired. A solution containing one equivalent of Pd(NO_3_)_2_ in DMSO-*d*_6_ was added to a separate solution containing two equivalents of ligand **L1** in DMSO-*d*_6_. The ^1^H NMR spectrum of the resulting solution showed formation of a single discrete complex. The reaction was repeated in DMSO and the PdL_2_-type complex [Pd(**L1**)_2_](NO_3_)_2_ (**3a**, Scheme 1c) was isolated from the reaction mixture by precipitation as described in the experimental section. Counter-anion (BF_4_^−^, ClO_4_^−^ and OTf^−^) variation was also carried out to successfully prepare a series of complexes [Pd(**L1**)_2_](BF_4_)_2_ (**3b**), [Pd(**L1**)_2_](ClO_4_)_2_ (**3c**), and [Pd(**L1**)_2_](OTf)_2_ (**3d**). These complexes were prepared by complexation of Pd(Y)_2_ with the ligand **L1** where Y = BF_4_^−^, ClO_4_^−^, and OTf^−^. It is important to note that Pd(Y)_2_ solutions were prepared by reacting PdI_2_ with AgY and the precipitated AgI was removed by filtration. Following this procedure, the presence of iodide as impurity could not be ruled out but its presence was found to not influence the formation of the targeted complex. In contrast, the presence of chloride remaining as impurity when PdCl_2_ was reacted with AgY to prepare Pd(Y)_2_, contaminated Pd(Y)_2_ and produced upon complexation with **L1** complexes **3a**–**d** along with some other products. The choice of PdI_2_ is on the basis of our previous experience from related cages [23]. The complex **3a** was characterized by various NMR techniques (Supporting Information File 1, Figures S27–S31). The ^1^H NMR spectrum of compound **3a** showed a single set of peaks (Figure 3) featured with complexation-induced downfield shifts of protons belonging to the terminal pyridines (Δδ = 0.79, and 0.47 ppm for H_a_, and H_b_, respectively) as compared to the free ligand **L1**.

The peak position of H_f_ did not change indicating that the central pyridine is not involved in the complexation. The ^1^H NMR spectra of compounds **3b**, **3c** and **3d** are very much comparable to that of **3a** (Supporting Information File 1, Figure S32). One of the coordination sites of the ligand **L1** thus remained unutilized in these mononuclear complexes.

The ESIMS spectrum of compound **3a** (Supporting Information File 1, Figure S33) showed isotopic peak patterns at *m*/*z* 866.10 and 402.05, respectively, which corresponds to the cationic fragments [**3a** − NO_3_]^+^ and [**3a** − 2NO_3_]^2+^ that are formed due to the loss of one and two units of counter anions from **3a**. The experimental and theoretical peak patterns were found to be in agreement. The ESIMS spectrum of **3b** is provided in Supporting Information File 1, Figure S34.

### Complexation of bare palladium(II) with ligand **L1** at a 3:4 metal-to-ligand ratio

A solution containing three equivalents of commercially acquired Pd(NO_3_)_2_ in DMSO-*d*_6_ was added to the solution containing four equivalents of ligand **L1** in DMSO-*d*_6_. The ^1^H NMR spectrum of the resulting solution recorded within 10 min showed a mixture of products. However, a single discrete complex was formed upon heating the solution at 90 °C for 5 min or upon stirring at rt for 20 min. A careful analysis of the ^1^H NMR spectrum revealed the existence of [Pd(**L1**)_2_](NO_3_)_2_ (**3a**) and [(NO_3_)_2_@Pd_3_(**L1**)_4_](NO_3_)_4_ (**4a**) in the mixture (Supporting Information File 1, Figure S35a). The mononuclear complex **3a** is proposed here as a kinetically controlled product and the trinuclear **4a** as the thermodynamic product. The reaction was repeated in DMSO and the Pd_3_L_4_-type complex **4a** (Scheme 1d) was isolated from the reaction mixture by precipitation as described in the experimental section. Complexation of Pd(Y)_2_ (prepared from PdI_2_ and AgY, where Y = BF_4_^−^, ClO_4_^−^, and OTf^−^) with the ligand **L1** at a 3:4 ratio resulted in the formation of only mononuclear complexes **3b–d** (depicted in a later Scheme) and the unutilized proportion of Pd(Y)_2_ remained in solution. Thus, the counter ion nitrate has a determining role by acting as template for the cavities in the formation of the trinuclear complex **4a**. It is proposed that the anion is essential to avoid charge repulsion between the metal centers in the ensuing cavities. However, the anions should be of fitting sizes only to get accommodated in the cavities so that discrete architectures are formed. Larger anions such as BF_4_^−^, ClO_4_^−^ and OTf^−^ are not accommodated in the cavities and not helpful as templates. The Pd(NO_3_)_2_ sample prepared from PdCl_2_ and AgNO_3_, contained chloride as impurity and resulted in a mixture of products along with the targeted **4a** (Supporting Information File 1, Figure S35b). The products in the mixture were identified as [(Cl)(NO_3_)@Pd_3_(**L1**)_4_](NO_3_)_4_ (**6a’**) and [(Cl)_2_@Pd_3_(**L1**)_4_](NO_3_)_4_ (**6a**) and their proportion was found to depend on the amount of chloride as impurity. The influence of chloride on the product composition is discussed in a later section.

The complex **4a** was characterized by various NMR techniques (Supporting Information File 1, Figures S37–S41). The ^1^H NMR spectrum of compound **4a** showed a single set of peaks (Figure 3) featured with complexation-induced downfield shifts of protons belonging to the terminal as well as to the central pyridine rings (Δδ = 1.42, 0.82, 1.02 ppm for H_a_, H_b_, and H_f_, respectively) as compared to the free ligand **L1**. The signal of H_f_ also got downfield-shifted indicating that the central pyridine ring is involved in the complexation.

The ESIMS spectrum of compound **4a** confirmed the formation of a trinuclear complex (Supporting Information File 1, Figure S42). Isotopic peak patterns are found at *m*/*z* 982.04, 634.03 and 460.03, which correspond to the cationic fragments [**4a** − 2NO_3_]^2+^_,_ [**4a** − 3NO_3_]^3+^ and [**4a** − 4NO_3_]^4+^ that are formed due to the loss of two, three and four units of counter anions from **4a**. The experimental and theoretical peak patterns were found to be in agreement.

### DFT studies of the complexes

**Figure 4:**
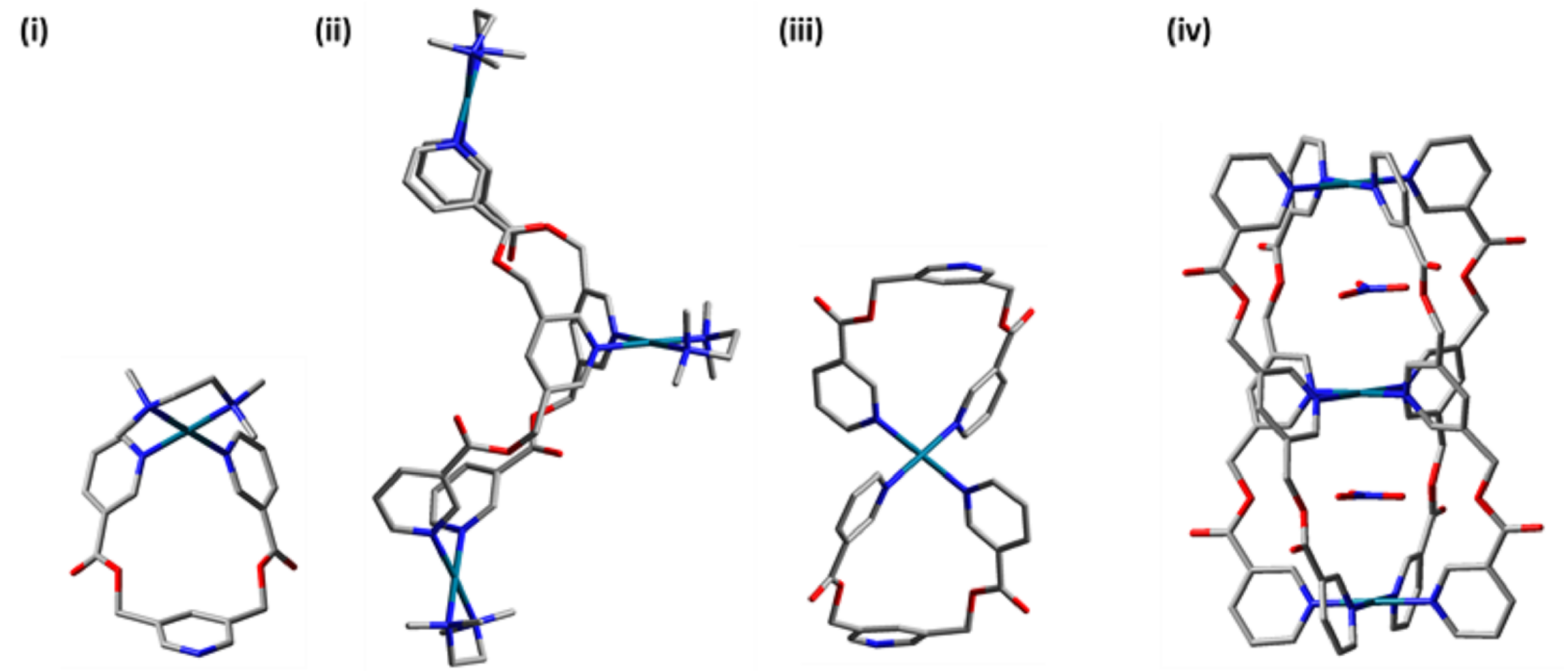
Energy-minimized structures of (i) [Pd(tmeda)(**L1**)]^2+^, (ii) [Pd_3_(tmeda)_3_(**L1**)_2_]^6+^, (iii) [Pd(**L1**)_2_]^2+^, and (iv) [(NO_3_)_2_@Pd_3_(**L1**)_4_]^4+^. Hydrogen atoms are omitted for clarity, red, blue, grey and cyan colors represent oxygen, nitrogen, carbon and palladium, respectively.

The energy-minimized structures of [Pd(tmeda)(**L1**)]^2+^, [Pd_3_(tmeda)_3_(**L1**)_2_]^6+^, [Pd(**L1**)_2_]^2+^, and [(NO_3_)_2_@Pd_3_(**L1**)_4_]^4+^ are shown in Figure 4 (see Supporting Information File 1 for details). Geometry optimization and calculation of frequencies were performed using Gaussian 09 software package at the B3LYP/6-31G* level of theory [37]. Since the complex [Pd_3_(tmeda)_3_(**L1**)_2_]^6+^ could not be prepared exclusively, we looked into the energetics of the system. The overall Gibbs free energies (∆*G*) and the enthalpies (∆*H*) for the formation of the trinuclear complex [Pd_3_(tmeda)_3_(**L1**)_2_]^6+^ considering its formation from 1 equivalent of [Pd(tmeda)(NO_3_)_2_] and 2 equivalents of [Pd(tmeda)(**L1**)]^2+^ were found to be not feasible (616.349 kcal mol^−1^) and endothermic (+537.727 kcal mol^−1^), respectively (see Figure S71 and Table S3 in Supporting Information File 1). However, a small amount of the trinuclear complex was formed experimentally. Probably, the counter anions stayed in the hemi-cage part of the trinuclear structure making it somewhat feasible. A detailed investigation of solvent and counter anion might help.

### Complex-to-complex transformations: **3a** versus **4a**

**Scheme 2:**
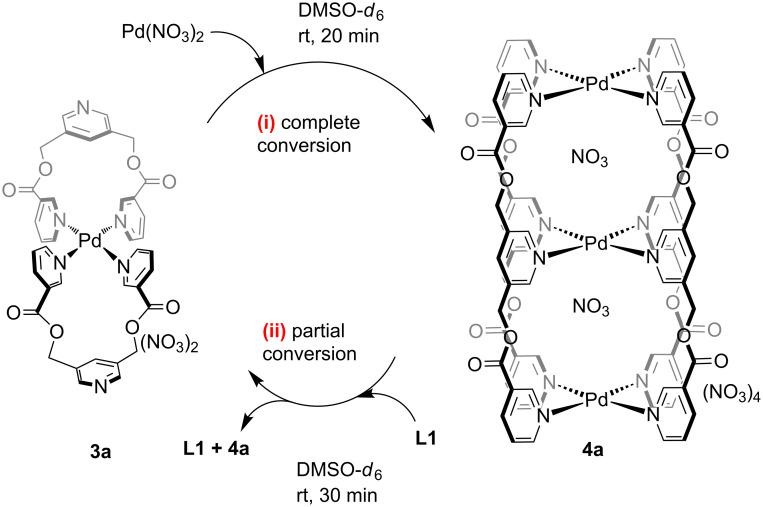
Reorganization of (i) a mixture of Pd(NO_3_)_2_ and **3a** at a 2:1 ratio leading to **4a** with a complete conversion and (ii) a mixture of **L1** and **4a** at a 2:1 ratio leading to **3a** but with a partial conversion.

The in situ prepared mononuclear complex [Pd(**L1**)_2_](NO_3_)_2_ (**3a**) was found to be stable at room temperature for days in DMSO*-d*_6_ (Supporting Information File 1, Figure S43) but not upon heating. The ^1^H NMR spectrum recorded after heating the solution at 90 °C for 24 h revealed decomplexation and signals for the free ligand were observed (Supporting Information File 1, Figure S44). In addition, the solution turned dark and dark particles were observed. Upon cooling the solution, the free ligand should have undergone complexation to form **3a**. However, no complexation was observed and it is assumed that palladium(II) got reduced to palladium(0). In another experiment, Pd(NO_3_)_2_ was added to a solution of **3a** at a 2:1 ratio where upon complex-to-complex conversion was observed at room temperature or upon heating to afford **4a** (Scheme 2(i)). With the appropriate amount of Pd(NO_3_)_2_ a complete formation of **4a** was observed within 20 min at rt or 5 min at 90 °C (Supporting Information File 1, Figures S45 and S46).

On the other hand, the in situ prepared trinuclear complex **4a** was found to be stable at room temperature as well as at 90 °C for days (Supporting Information File 1, Figures S47 and S48). The free ligand **L1** was added to a solution of **4a** in DMSO-*d*_6_ at room temperature and the sample was monitored by ^1^H NMR spectroscopy. The calculated amount of ligand was added to the solution of **4a** (at 2:1 ratio) to match the stoichiometric requirement for the formation of **3a**. Although the formation of **3a** was observed, it remained only as a minor product, and the added ligand **L1** was partially consumed. Thus, the unbound ligand remained in its free state along with **4a**. No further change was observed after 30 min (Supporting Information File 1, Figure S49). Heating of the reaction mixture did not help in further pushing the conversion towards the formation of **3a** (Supporting Information File 1, Figure S50). Prolonged heating could not help because the complex **3a** is unstable under such conditions. This provided additional support on the higher stability of **4a** as compared to **3a**.

### Halide binding by the cavities of a double-decker cage

**Scheme 3:**
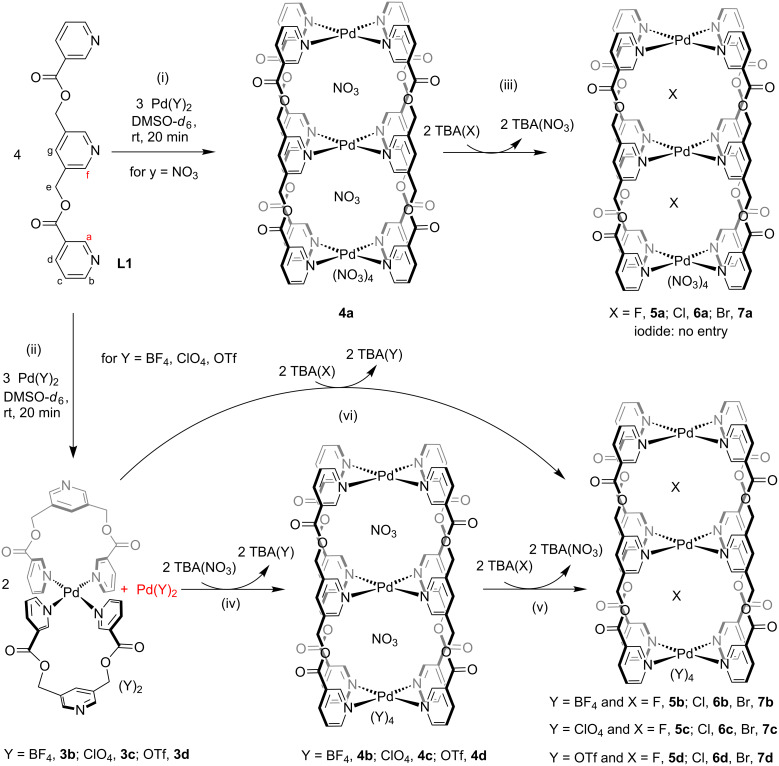
Halide (F^−^, Cl^−^ and Br^−^ but not I^−^) encapsulation by the cavities of the double-decker cage.

The trinuclear complex [(NO_3_)_2_@Pd_3_(**L1**)_4_](NO_3_)_4_ (**4a**) was prepared by mixing Pd(NO_3_)_2_ with ligand **L1** at a 3:4 ratio (Scheme 3(i), also Scheme 1(iv)). Complexation of metal components like Pd(BF_4_)_2_, Pd(ClO_4_)_2_ or Pd(OTf)_2_ with **L1** at a 3:4 ratio did not afford the analogous trinuclear complexes; rather the corresponding mononuclear complexes were formed and the uncomplexed palladium(II) remained in solution (Scheme 3(ii)).

**Figure 5:**
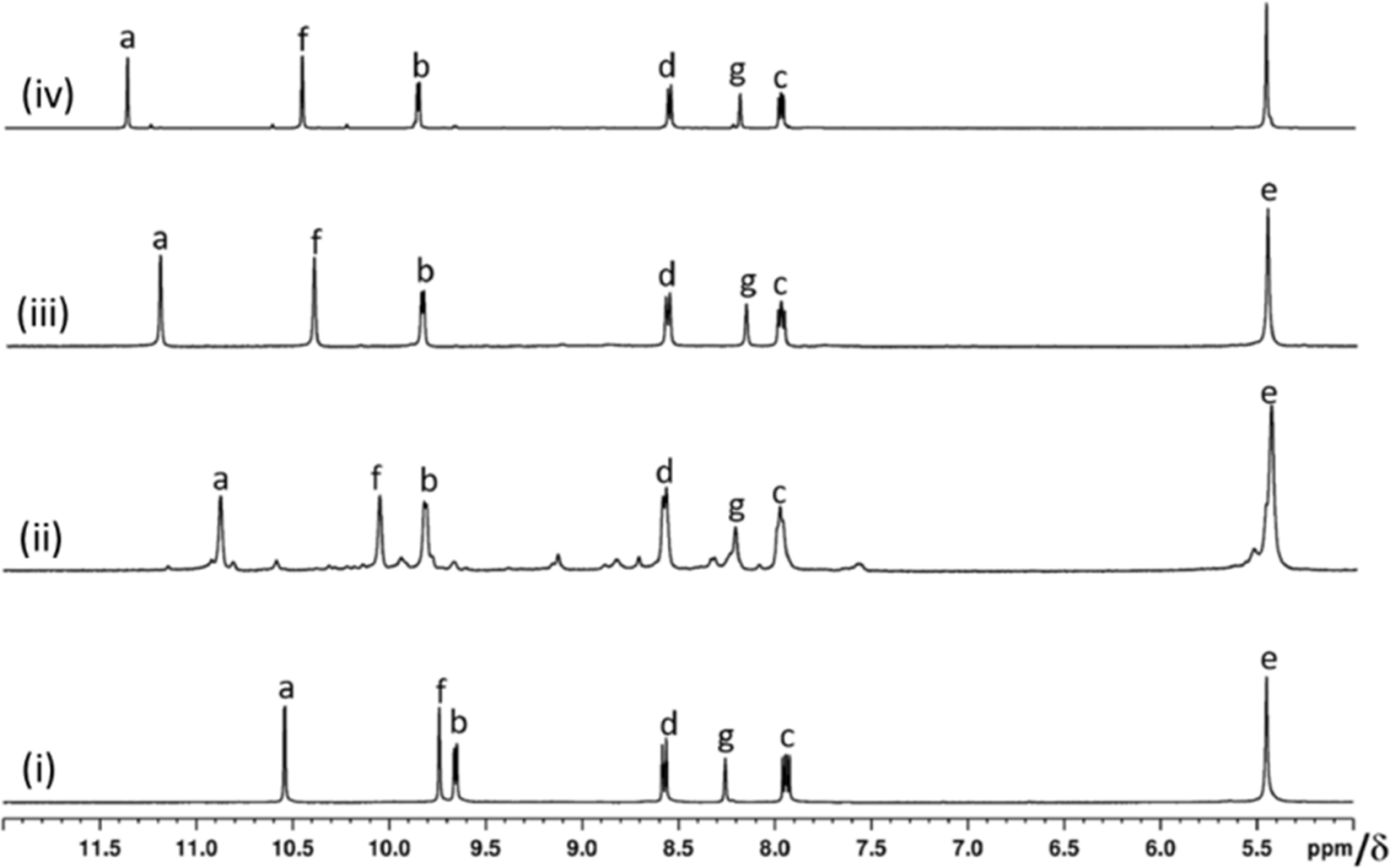
Partial ^1^H NMR spectra at 400 MHz in DMSO-*d*_6_ for (i) [(NO_3_)_2_@Pd_3_(**L1**)_4_](NO_3_)_4_ (**4a**), (ii) [(F)_2_@Pd_3_(**L1**)_4_](NO_3_)_4_ (**5a**), (iii) [(Cl)_2_@Pd_3_(**L1**)_4_](NO_3_)_4_ (**6a**) and (iv) [(Br)_2_@Pd_3_(**L1**)_4_](NO_3_)_4_ (**7a**).

Each of the two cavities of cage **4a** is loaded with one NO_3_^–^. This phenomenon of NO_3_^–^ encapsulation by a related isomeric cage was established by us earlier [22–23]. Halide recognition by the complex **4a** through anion exchange was studied by portionwise addition of freshly prepared solutions of tetra-*n*-butylammonium halide, i.e., TBA(X) (where X stands for F^−^, Cl^−^, Br^−^ and I^−^) in four separate experiments using DMSO-*d*_6_ as the solvent. The anion exchange processes were monitored by ^1^H NMR spectroscopy of the samples (Supporting Information File 1, Figures S51–S54). The addition of a portion of TBACl to the complex **4a** resulted in a mixture of **4a**, [(Cl)(NO_3_)@Pd_3_(**L1**)_4_](NO_3_)_4_ (**6a’**) and [(Cl)_2_@Pd_3_(**L1**)_4_](NO_3_)_4_ (**6a**). With further addition of TBACl, the proportion of **6a** increased at the cost of **4a** and **6a’** to finally yield compound **6a** as the only product. Similarly, the addition of TBABr initially produced a mixture of **4a**, [(Br)(NO_3_)@Pd_3_(**L1**)_4_](NO_3_)_4_ (**7a’**) and [(Br)_2_@Pd_3_(**L1**)_4_](NO_3_)_4_ (**7a**) with complex **7a** as the exclusive final product. In the case of TBAF, initially there was no change observable except a slight broadening of the signals for H_b_ and H_c_. Further addition of TBAF led to the formation of [(F)_2_@Pd_3_(**L1**)_4_](NO_3_)_4_ (**5a**) along with minor impurities. However, the addition of TBAI to **4a** showed no changes and hence iodide encapsulation did not happen. The ^1^H NMR spectra for the mixtures of products formed at intermediate and final stages are provided in Supporting Information File 1 (Figures S51–S54) and those of **4a**, **5a**, **6a** and **7a** are shown in Figure 5. The positions of the signals in the ^1^H NMR spectra of these anion-encapsulated complexes are influenced by coordination of ligand **L1** with palladium(II) and interaction of the encapsulated anion with the endohedrally oriented hydrogens of the cages.

Is iodide not capable of replacing the preexisting nitrate in a competition or iodide is not suited at all for the cavity irrespective of any competition? The following argument might answer this question. The complexation reaction shown in steps (ii) of Scheme 3 suggest that the presence of BF_4_^−^, ClO_4_^−^ or OTf^−^ could not support the formation of the double-decker cage even though the required amount of palladium(II) was available. The addition of TBAI to any of these solutions containing Pd(Y)_2_ and **3b**, **3c** or **3d**, respectively, did not lead to double-decker cages indicates that I^−^ is not suited for the cavity. However, addition of TBANO_3_, TBAF, TBACl and TBABr produced the corresponding anion encapsulated double-decker cages as shown in steps (iv), (v) and (vi) of Scheme 3. Representative ^1^H NMR spectra for the conversion of **3b** to corresponding products **4b**, **5b**, **6b** and **7b** are shown in Supporting Information File 1 (Figure S55).

The ^1^H NMR spectral analysis of the two nitrate anions incorporating compound **4a** discussed in an earlier section revealed downfield shifts of particular signals as compared to the free ligand **L1** and the Δδ values were 1.42, 0.82 and 1.02 ppm for the signals of H_a_, H_b_, and H_f_, respectively. A similar comparison for the (i) two F^−^ encapsulated compound **5a**: Δδ = 1.75, 0.99 and 1.78 ppm for H_a_, H_b_, and H_f_, respectively), (ii) the two Cl^−^ encapsulated compound **6a**: (Δδ = 1.91, 1.01 and 1.69 ppm for H_a_, H_b_ and H_f_, respectively), and (iii) the two Br^−^ encapsulated compound **7a**: (Δδ = 2.24, 1.02 and 1.74 ppm for H_a_, H_b_ and H_f_, respectively) are in line with the expectation. Although fluoride (F^–^) could replace NO_3_^–^ in **4a** to afford complex **5a**, the complex **5a** was found to be unstable and it decomposed within a few hours. Thus the ^1^H NMR and ESIMS spectrum of **5a** were recorded from freshly prepared samples. The complexes **4a**, **6a** and **7a** are quite stable and no decomposition was observed. Detailed characterization data of **4a**–**7a** form a variety of NMR techniques are provided in Supporting Information File 1, Figures S56–S66.

The molecular compositions of the halide encapsulated complexes were confirmed by recording ESIMS data for the systems. The double halide encapsulated complexes **6a** and **7a** were detected. Also, one of the mixed halide–nitrate encapsulated complexes, i.e., **7a’** was also detected.

The ESIMS spectrum of compound **6a** (Supporting Information File 1, Figure S67) showed isotopic peak pattern at *m*/*z =* 956.02 which corresponds to the cationic fragment [**6a** − 2NO_3_]^2+^ that was formed due to the loss of two counter anions from **6a**. The ESIMS spectrum of compound **7a** (Supporting Information File 1, Figure S68) showed isotopic peak patterns at *m*/*z =* 645.99 and at 468.99 which correspond to the cationic fragments [**7a** − 3NO_3_]^3+^ and [**7a** − 4NO_3_]^4+^ that are formed due to the loss of three and four units of counter anions from **7a**. The ESIMS spectrum of compound **7a’** (Supporting Information File 1, Figure S69) showed isotopic peak pattern at *m*/*z =* 991.51 which corresponds to the cationic fragment [**7a’** − 2NO_3_]^2+^ that is formed due to the loss of two units of counter anions from **7a’**. The experimental and theoretical peak patterns were found to be in agreement.

### Coordination complexes of **L1** versus **L2**: ligand isomerism phenomenon

As discussed in the introduction section “The definition of ligand isomerism includes metal complexes (at least two) having the same molecular formula but are composed of different structural isomers of the ligand.” The complexes prepared in this work namely [Pd(tmeda)(**L1**)](NO_3_)_2_ (**1a**), [Pd(**L1**)_2_](NO_3_)_2_ (**3a**), [(NO_3_)_2_@Pd_3_(**L1**)_4_](NO_3_)_4_ (**4a**), and [(X)_2_@Pd_3_(**L1**)_4_](NO_3_)_4_ **5a**–**7a** fulfill the definition of ligand isomerism when compared with the reported complexes [Pd(tmeda)(**L2**)](NO_3_)_2_ (**8a**), [Pd(**L2**)_2_](NO_3_)_2_ (**10a**), [(NO_3_)_2_@Pd_3_(**L2**)_4_](NO_3_)_4_ (**11a**), and [(X)_2_@Pd_3_(**L2**)_4_](NO_3_)_4_ **12a**–**14a**, respectively. We have demonstrated ligand isomerism in Pd_2_L_4_-type cages [29]. The present work demonstrates ligand isomerism in some complexes and more interestingly for the Pd_3_L_4_-type double-decker coordination cages for the first time.

### Palladium(II)-based self-assembled complexes of ligands **L1** and **L2**: a comparison

The complexation behavior of **L1** and **L2** are broadly comparable. However, a closer look revealed certain differences. While complexation of *cis*-Pd(tmeda)(NO_3_)_2_ with **L1** produced the trinuclear complex [Pd_3_(tmeda)_3_(**L1**)_2_](NO_3_)_6_ (**2a**), the ligand **L2** did not afford the targeted [Pd_3_(tmeda)_3_(**L1**)_2_](NO_3_)_6_ (**9a**). The complex [Pd(**L1**)_2_](NO_3_)_2_ (**3a**) was unstable when heated in DMSO medium whereas the corresponding complex [Pd(**L2**)_2_](NO_3_)_2_ (**10a**) was stable under comparable conditions. The addition of two equivalents of **L1** to a solution of [(NO_3_)_2_@Pd_3_(**L1**)_4_](NO_3_)_4_ (**4a**) produced only a small amount of [Pd(**L1**)_2_](NO_3_)_2_ (**3a**) and the added ligand remained in solution. On the other hand, addition of the required amount of **L2** to a solution of [(NO_3_)_2_@Pd_3_(**L2**)_4_](NO_3_)_4_ (**11a**) resulted in complete transformation to complex Pd(**L2**)_2_](NO_3_)_2_ (**10a**). The F^−^ encapsulated complex [(F)_2_@Pd_3_(**L1**)_4_](NO_3_)_4_ (**5a**) decomposed within a few hours whereas [(F)_2_@Pd_3_(**L2**)_4_](NO_3_)_4_ (**12a**) was stable for a few hours. These differences are ascribed to the positional exchanged functionalities in the ligands **L1** and **L2**. Probably, the coordination ability of the central pyridine ring is better than that of the terminal pyridine rings in case of **L1**. However, in the mononuclear complexes of **L1** the central pyridine remained uncomplexed, which may be due to the formation of metallomacrocyclic rings. This behavior is not observed in the case of mononuclear complexes of **L2**. Thus, the mononuclear complexes of **L1** are reluctant to form (e.g., from **4a** and **L1**) and prone to decomposition. As far as trinuclear complex formation is concerned the central pyridine ring of **L1** is in a relatively favorable situation, thus the complex **4a** could form and **3a** was a kinetic product.

## Conclusion

A set of mononuclear and trinuclear complexes were prepared through complexation of *cis*-protected palladium(II) and bare palladium(II) components with the new tridentate ligand **L1**. A variety of counter anions were employed to broaden the scope of the choice of metal components. Mononuclear complexes with PdL’L composition could be prepared easily, however, Pd_3_L’_3_L_2_-type trinuclear complexes were obtained in only small amounts. Also, mononuclear complexes of PdL_2_ and trinuclear complexes of Pd_3_L_4_-type compositions were prepared easily. The choice of the counter anion did not influence the formation of mononuclear complexes whereas the counter anion displayed a template role for the formation of trinuclear complexes, especially for Pd_3_L_4_-type complexes. The anions helped to screen the charge repulsion between the palladium(II) ions. The complexation behavior of palladium(II) components with the ligand **L2** have been reported earlier [23]. The similarities and differences in the complexation behaviors of the ligands **L1** and **L2** were highlighted. A qualitative comparison indicated that ligands **L1** and **L2** are well suited for the formation of trinuclear only and mononuclear/trinuclear complexes, respectively. The Pd_3_L’_3_L_2_-type complexes could be prepared, though in small proportions, using ligand **L1** but not **L2**. The ligands **L1** and **L2** are positional isomers (regioisomers) hence many of their complexes could be rightfully considered under ligand isomerism in coordination complexes.

## Experimental

**Synthesis of ligand L1:** A mixture of pyridine-3,5-diyldimethanol (282.6 mg, 2.03 mmol) and nicotinoyl chloride hydrochloride (500.0 mg, 4.06 mmol) in dry DCM (50 mL) was placed in a 100 mL round-bottomed flask. The flask was placed in an ice bath to cool the mixture followed by the dropwise addition of triethylamine (2 mL). Then, the reaction mixture was stirred at room temperature for 24 h followed by the addition of a saturated aqueous solution of sodium bicarbonate. The organic layer was separated and the solvent was evaporated using a rotavapor. The crude product was purified by column chromatography using EtOAc/hexane 8:2 to afford the product as white solid (507.3 mg, isolated yield 71%) after evaporation of the solvent and drying under vacuum. Mp 124 °C; ^1^H NMR (500 MHz, DMSO-*d*_6_, 300 K) δ 9.13 (dd, *J*_1_ = 2.8 Hz, *J*_2_ = 1.5 Hz, 1H, H_a_), 8.83 (dd, *J*_1_ = 6.5 Hz, *J*_2_ = 3.2 Hz, 1H, H_b_), 8.72 (d, *J* = 2.0 Hz, 1H, H_f_), 8.34–8.32 (m, 1H, H_d_), 8.09 (t, *J* = 2.0 Hz, 1H, H_g_), 7.58–7.55 (m, 1H, H_c_), 5.47 (s, 2H, H_e_); ^13^C NMR (100 MHz, DMSO-*d*_6_*,* 300 K) δ 164.59, 153.88, 150.12, 149.10, 137.01, 135.62, 131.46, 125.37, 123.97, 64.13; ESIMS (*m*/*z*)*:* 372.098 [M + Na]^+^.

**[Pd(tmeda)(L1)](NO_3_)_2_ (1a):** To a solution of *cis*-Pd(tmeda)(NO_3_)_2_ (10.3 mg, 0.03 mmol), in 3 mL of DMSO ligand **L1** (10.5 mg, 0.03 mmol) was added. The reaction mixture was stirred at room temperature for 10 min to obtain a clear yellow solution. The product was precipitated by the addition of ethyl acetate (10 mL), separated by centrifugation, washed with acetone (4 mL) and dried under vacuum to afford complex **1a** (16.8 mg, isolated yield 80%). ^1^H NMR (500 MHz, DMSO-*d*_6_, 300 K) δ 9.90 (d, *J* = 1.5 Hz, 1H, H_a_), 9.35 (dd, *J*_1_ = 1.0 Hz, *J*_2_ = 5.8 Hz, 1H, H_b_), 8.67 (s, 1H, H_f_), 8.54–8.52 (m, 1H, H_d_), 8.18 (s, 1H, H_g_), 7.85 (dd, *J*_1_ = 7.9 Hz, *J*_2_ = 5.8 Hz, 1H, H_c_), 5.91–5.29 (dd, *J*_1_ = 13.8 Hz, *J*_2_ = 13.8 Hz, 2H, H_e_); ^13^C NMR (125 MHz, DMSO-*d*_6_*,* 300 K) δ 164.50, 154.60, 151.91, 147.21, 140.92, 133.03, 132.24, 129.41, 127.31, 63.41, 62.31.

**[Pd(L1)_2_](NO_3_)_2_ (3a):** To a solution of ligand **L1** (20.9 mg, 0.06 mmol) in 3 mL of DMSO, Pd(NO_3_)_2_ (6.9 mg, 0.03 mmol) was added and the reaction mixture was stirred for 10 min at room temperature to give a clear yellow solution. The product was precipitated by the addition of 10 mL of ethyl acetate. The pale yellow precipitate was separated by centrifugation, washed with acetone and dried under vacuum to afford complex **3a** (18.6 mg, isolated yield 66%). ^1^H NMR (500 MHz, DMSO-*d*_6_, 300 K) δ 9.92 (s, 1H, H_a_), 9.30 (d, *J* = 5.3 Hz, 1H, H_b_), 8.75 (d, *J* = 1.3 Hz, 1H, H_f_), 8.55–8.53 (m, 1H, H_d_), 8.31 (s, 1H, H_g_), 7.82 (dd, *J*_1_ = 7.9 Hz, *J*_2_ = 5.8 Hz, 1H, H_c_), 5.63 (s, 1H, H_e_); ^13^C NMR (125 MHz, DMSO-*d*_6_*,* 300 K) δ 162.43, 154.57, 151.59, 147.83, 141.30, 133.97, 132.31 129.57, 127.38, 63.86; ESIMS (*m/z)*: 866.10 [**3a** − 1NO_3_]^+^; 402.05 [**3a** − 2NO_3_]^2+^.

**[(NO_3_)_2_@Pd_3_(L1)_4_](NO_3_)_4_ (4a):** To a solution of ligand **L1** (14.0 mg, 0.04 mmol) in 2 mL of DMSO, Pd(NO_3_)_2_ (7.0 mg, 0.03 mmol) in 1 mL of DMSO was added and the reaction mixture was stirred for 5 min at 90 °C to give a clear yellow solution. The product was precipitated by the addition of 10 mL of ethyl acetate. The pale yellow precipitate was separated by centrifugation, washed with acetone and dried under vacuum to afford the complex **4a** (15.6 mg, isolated yield 75%). ^1^H NMR (500 MHz, DMSO-*d*_6_, 300 K) δ 10.54 (d, *J* = 1.4 Hz, 1H, H_a_), 9.74 (s, 1H, H_f_), 9.66 (d, *J* = 5.0 Hz, 1H, H_b_), 8.58 (d, *J* = 8.0 Hz, 1H, H_d_), 8.26 (s, 1H, H_g_), 7.95–7.93 (m, 1H, H_c_), 5.45 (s, 1H, H_e_); ^13^C NMR (125 MHz, DMSO-*d*_6_, 300 K) δ 162.40, 155.05, 153.19, 149.91, 141.88, 138.39, 133.83, 128.66, 127.68, 64.84; ESIMS (*m/z*): 982.04 [**4a** − 2NO_3_]^2+^; 634.03 [**4a** − 3NO_3_]^3+^; 460.03 [**4a** − 4NO_3_]^4+^.

## Supporting Information

File 1 Experimental procedures, NMR, ESIMS data, and theoretical study.
