# Supplementary material for: Design of a double-decker coordination cage revisited to make new cages and exemplify ligand isomerism
Source: Beilstein J Org Chem. 2019 May 21;15:1129–40. doi: 10.3762/bjoc.15.109 (PMC6541329; doi:10.3762/bjoc.15.109)
Supplement: File 1 — Experimental procedures, NMR, ESIMS data, and theoretical study. [file Beilstein_J_Org_Chem-15-1129-s001.pdf]

BEILSTEIN JOURNAL OF ORGANIC CHEMISTRY

Supporting Information

for

Design of a double-decker coordination cage revisited to make new cages and exemplify ligand isomerism

Sagarika Samantray, Sreenivasulu Bandi and Dillip K. Chand

Beilstein J. Org. Chem. **2019,** *15,* 1129–1140. doi:10.3762/bjoc.15.109

Experimental procedures, NMR, ESIMS data, and theoretical study

License and Terms: This is a supporting information file under the terms of the Creative Commons Attribution License (http://creativecommons.org/licenses/by/4.0). Please note that the reuse, redistribution and reproduction in particular requires that the authors and source are credited.
The license is subject to the *Beilstein Journal of Organic Chemistry* terms and conditions: (https://www.beilstein-journals.org/bjoc)

Table of Contents

Section S1. Materials and instruments

Section S2. Numbering scheme of the compounds (Chart 1)

Section S3. Synthesis of [Pd(tmeda)(**L1**)]$(Y)_2$, **1b-1d**. (Y = BF_4, ClO_4 and OTf)

Section S4. Synthesis of [Pd$(\mathbf{L1})_2$]$(Y)_2$, **3b-3d**. (Y = BF_4, ClO_4 and OTf)

Section S5. Synthesis of $[(X)_2@Pd_3(\mathbf{L1})_4](NO_3)_4$, **5a-7a** (where X= F, Cl or Br)

Section S6. Synthesis of $[(NO_3)_2@Pd_3(\mathbf{L1})_4](BF_4)_4$, **4b** and $[(X)_2@Pd_3(\mathbf{L1})_4](BF_4)_4$, **5b-7b** (where X= F, Cl or Br)

Section S7. Figures (**S1** to **S69**) depicting NMR spectra and ESIMS of the ligands, complexes, and anion encapsulated complexes. NMR spectra to support dynamic behaviors of the complexes.

Section S8: Figures S70, S71), Tables S2, S3) and explanatory note related to theoretical study. (Energy minimized structures of various components and self-assembled compounds, calculated data and explanation)

<u>List of Figures S1–S69 and Table S1 related to section S7:</u>

Figure S1: ^{1}H NMR (400 MHz, $CDCl_3$, 300 K) for **L1**.

Figure S2: ^{13}C NMR (125 MHz, $CDCl_3$, 300 K) for **L1**.

Figure S3a: H-H COSY (500 MHz, $CDCl_3$, 300 K) for **L1**.

Figure S3b: H-H COSY expansion (500 MHz, $CDCl_3$, 300 K) for **L1**.

Figure S4a: C-H COSY (500 MHz, $CDCl_3$, 300 K) for **L1**.

Figure S4b: C-H COSY expansion (500 MHz, $CDCl_3$, 300 K) for **L1**.

Figure S5a: NOESY (500 MHz, $CDCl_3$, 300 K) for **L1**.

Figure S5b: NOESY expansion (500 MHz, $CDCl_3$, 300 K) for **L1**.

Figure S6: ^{1}H NMR (500 MHz, DMSO-d_6, 300 K) for **L1**.

Figure S7: ^{13}C NMR (125 MHz, DMSO-d_6, 300 K) for **L1**.

Figure S8a: H-H COSY (500 MHz, DMSO-d_6, 300 K) for **L1**.

Figure S8b: H-H COSY expansion (500 MHz, DMSO-d_6, 300 K) for **L1**.

Figure S9a: C-H COSY (500 MHz, DMSO-d_6, 300 K) for **L1**.

Figure S9b: C-H COSY expansion (500 MHz, DMSO-d_6, 300 K) for **L1**.

Figure S10a: NOESY (400 MHz, DMSO-d_6, 300 K) for **L1**.

Figure S10b: NOESY expansion (500 MHz, DMSO-d_6, 300 K) for **L1**.

Figure S11: ESI-MS for **L1** showing $(M+Na)^+$**.**

Figure S12: ^{1}H NMR (500 MHz, DMSO-d_6, 300 K) for [Pd(tmeda)(**L1**)]$(NO_3)_2$, **1a**.

Figure S13: ^{13}C NMR (125 MHz, DMSO-d_6, 300 K) for [Pd(tmeda)(**L1**)]$(NO_3)_2$, **1a**.

Figure S14a: H-H COSY (500 MHz, DMSO-d_6, 300 K) for [Pd(tmeda)(**L1**)]$(NO_3)_2$, **1a**.

Figure S14b : H-H COSY expansion (500 MHz, DMSO-d_6, 300 K) for [Pd(tmeda)(**L1**)]$(NO_3)_2$, **1a**.

Figure S15a: C-H COSY (500 MHz, DMSO-d_6, 300 K) for [Pd(tmeda)(**L1**)]$(NO_3)_2$, **1a**.

Figure S15b: C-H COSY expansion (500 MHz, DMSO-d_6, 300 K) for [Pd(tmeda)(**L1**)]$(NO_3)_2$, **1a**.

Figure S16a: NOESY (500 MHz, DMSO-d_6, 300 K) for [Pd(tmeda)(**L1**)]$(NO_3)_2$, **1a**.

Figure S16b: NOESY expansion (500 MHz, DMSO-d_6, 300 K) for [Pd(tmeda)(**L1**)]$(NO_3)_2$, **1a**.

Figure S17: Partial ^{1}H NMR spectra (400 MHz, DMSO-d_6, 300 K) for (i) ligand **L1**; (ii)[Pd(tmeda)(**L1**)]$(NO_3)_2$**,** **1a**; (iii) [Pd(tmeda)(**L1**)]$(BF_4)_2$**,** **1b**;(iv) [Pd(tmeda)(**L1**)]$(ClO_4)_2$**,** **1c**; and (v) [Pd(tmeda)(**L1**)]$(OTf)_2$**,** **1d**.

Figure S18a: ESI-MS, isotopic pattern for $[$**1b**$-BF_4]^{+}$ i) experimental and ii) theoretical.

Figure S18b: ESI-MS, isotopic pattern for $[$**1b**$-2BF_4]^{2+}$ i) experimental and ii) theoretical.

Figure S19a: ESI-MS, isotopic pattern for $[$**1c**$-ClO_4]^{+}$ i) experimental and ii) theoretical.

Figure S19b: ESI-MS, isotopic pattern for $[$**1c**$-2ClO_4]^{2+}$ i) experimental and ii) theoretical.

Figure S20a: ESI-MS, isotopic pattern for $[$**1d**$-OTf]^{+}$ i) experimental and ii) theoretical.

Figure S20b: ESI-MS, isotopic pattern for $[$**1d**$-2OTf]^{2+}$ i) experimental and ii) theoretical.

Figure S21: Partial ^{1}H NMR spectra (400 MHz, DMSO-d_6, 300 K) for (i) ligand **L1**; (ii)-(iii) monitoring the reaction of **L1** and Pd(tmeda)$(NO_3)_2$ in 2:3 ratio (ii) after 24 h formation of [Pd(tmeda)(**L1**)] $(NO_3)_2$, **1a**; (iii) upon heating the mixture for further 72 h at 90 °C [Pd(tmeda)(**L1**)] $(NO_3)_2$,, **1a** and $[Pd_3(tmeda)_3($**L1**$)_2](NO_3)_6$, **2a** (20 percentage).

Figure S22: Partial ^{1}H NMR spectra (400 MHz, DMSO-d_6, 300 K) for (i) ligand **L1**; (ii)-(iii) monitoring the reaction of **L1** and Pd(tmeda)$(BF_4)_2$ in 2:3 ratio (ii) after 24 h formation of [Pd(tmeda)(**L1**)]$(BF_4)_2$, **1b**; (iii) upon heating the mixture for further 72 h at 90 °C there was no change complex [Pd(tmeda)(**L1**)]$(BF_4)_2$, **1b** retained as such.

Figure S23: Partial ^{1}H NMR spectra (400 MHz, DMSO-d_6, 300 K) for (i) ligand **L1**; (ii)-(iii) monitoring the reaction of **L1** and Pd(tmeda)$(ClO_4)_2$ in 2:3 ratio (ii) after 24 h formation of [Pd(tmeda)(**L1**)]$(ClO_4)_2$, **1c** and $[Pd_3(tmeda)_3($**L1**$)_2](ClO_4)_6$, **2c** (20 percentage) (iii) upon heating the mixture for further 72 h at 90 °C there was no change in the percentage of formation of $[Pd_3(tmeda)_3($**L1**$)_2](ClO_4)_6$, **2c**.

Figure S24: Partial ^{1}H NMR spectra (400 MHz, DMSO-d_6, 300 K) for (i) ligand **L1**; (ii)-(iii) monitoring the reaction of **L1** and Pd(tmeda)(**L1**)$(OTf)_2$ in 2:3 ratio (ii) after 24 h formation of [Pd(tmeda)(**L1**)]($OTf)_2$, **1d** and $[Pd_3(tmeda)_3$(**L1**$)_2$]($OTf)_6$, **2d** (20 percentage) (iii) upon heating the mixture for further 72 h at 90 °C there was no change in the percentage of formation of $[Pd_3(tmeda)_3$(**L1**$)_2$]$(OTf)_6$, **2d**. (Some amount of $[(Cl)_2{\subset}Pd_3$(**L1**$)_4$]$(OTf)_4$, **6d** detected unexpectedly, probably due to chloride impurity.)

Figure S25: ESI-MS, isotopic pattern for [**2c**-$4ClO_4]^{4+}$ i) experimental and ii) theoretical.

Figure S26: ESI-MS, isotopic pattern for [**2d**-OTf$]^+$ i) experimental and ii) theoretical.

Figure S27: ^{1}H NMR (400 MHz, DMSO-d_6, 300 K) for [Pd(**L1**$)_2$]$(NO_3)_2$, **3a**.

Figure S28: ^{13}C NMR (125 MHz, DMSO-d_6, 300 K) for [Pd(**L1**$)_2$]$(NO_3)_2$, **3a**.

Figure S29a: H-H COSY (500 MHz, DMSO-d_6, 300 K) for [Pd(**L1**$)_2$]$(NO_3)_2$, **3a**.

Figure S29b: Expansion of H-H COSY (500 MHz, DMSO-d_6, 300 K) for [Pd(**L1**$)_2$]$(NO_3)_2$, **3a**.

Figure S30a: C-H COSY (500 MHz, DMSO-d_6, 300 K) for [Pd(**L1**$)_2$]$(NO_3)_2$, **3a**.

Figure S30b: Expansion of C-H COSY (500 MHz, DMSO-d_6, 300 K) for [Pd(**L1**$)_2$]$(NO_3)_2$, **3a**.

Figure S31a: NOESY (500 MHz, DMSO-d_6, 300 K) for [Pd(**L1**$)_2$]$(NO_3)_2$, **3a**.

Figure S31b: Expansion for NOESY (500 MHz, DMSO-d_6, 300 K) for [Pd(**L1**$)_2$]$(NO_3)_2$, **3a**.

Figure S32: Partial ^{1}H NMR spectra (400 MHz, DMSO-d_6, 300 K) for (i) ligand **L1**; (ii) [Pd(**L1**$)_2$]$(NO_3)_2$, **3a**; (iii) [Pd(**L1**$)_2$]$(BF_4)_2$, **3b**; (iv) [Pd(**L1**$)_2$]$(ClO_4)_2$, **3c** and (v) [Pd(**L1**$)_2$]$(OTf)_2$, **3d**.

Figure S33a: ESI-MS, isotopic pattern for [**3a**-$NO_3]^+$ i) experimental and ii) theoretical.

Figure S33b: ESI-MS, isotopic pattern for [**3a**-$2NO_3]^{2+}$ i) experimental and ii) theoretical..

Figure S34a: ESI-MS, isotopic pattern for [**3b**-$BF_4]^+$ i) experimental and ii) theoretical.

Figure S34b: ESI-MS, isotopic pattern for [**3b**-$2BF_4]^{2+}$ i) experimental and ii) theoretical.

Figure S35a: Partial ^{1}H NMR spectra (400 MHz, DMSO-d_6, 300 K) for (i) ligand **L1**; (ii)-(iii) monitoring the reaction of **L1** and $Pd(NO_3)_2$ in 4 :3 ratio upon mixing at room temperature (ii) formation of mixture of $[Pd_1$(**L1**$)_2$]$(NO_3)_2$, **3a** (labelled in green) and $[(NO_3)_2@Pd_3$(**L1**$)_4$]$(NO_3)_4$, **4a** (labelled in blue) after 10 min; (iii) exclusive formation of $[(NO_3)_2@Pd_3$(**L1**$)_4$]$(NO_3)_4$, **4a** after 20 min.

Figure S35b: Partial ^{1}H NMR spectra (400 MHz, DMSO-d_6, 300 K) for (i) ligand **L1**; (ii) the presence of $[(NO_3)_2@Pd_3$(**L1**$)_4$]$(NO_3)_4$, **4a** peaks labelled in black color and

$[(NO_3)(Cl)@Pd_3($**L1**$)_4](NO_3)_4$, **5a'** peaks labelled in red color. [When the metal-to-ligand ratio was 3:4 and the $Pd(NO_3)_2$ was prepared from $PdCl_2$]

Figure S36: Partial ^1H NMR spectra (400 MHz, DMSO-d_6, 300 K) for (i) ligand **L1**; (ii) exclusive formation of $[(NO_3)_2@Pd_3($**L1**$)_4](NO_3)_4$, **4a** formed by stirring **L1** and $Pd(NO_3)_2$ in 4:3 ratio for 5 min at 90 °C.

Figure S37: ^1H NMR (500 MHz, DMSO-d_6, 300 K) for $[(NO_3)_2@Pd_3($**L1**$)_4](NO_3)_4$, **4a**.

Figure S38: ^{13}C NMR (125 MHz, DMSO-d_6, 300 K) for $[(NO_3)_2@Pd_3($**L1**$)_4](NO_3)_4$, **4a**.

Figure S39a: H-H COSY (500 MHz, DMSO-d_6, 300 K) for $[(NO_3)_2@Pd_3($**L1**$)_4](NO_3)_4$, **4a**.

Figure S39b: H-H COSY expansion (500 MHz, DMSO-d_6, 300 K) for $[(NO_3)_2@Pd_3($**L1**$)_4](NO_3)_4$, **4a**.

Figure S40a: C-H COSY (500 MHz, DMSO-d_6, 300 K) for $[(NO_3)_2@Pd_3($**L1**$)_4](NO_3)_4$, **4a**.

Figure S40b: C-H COSY expansion (500 MHz, DMSO-d_6, 300 K) for $[(NO_3)_2@Pd_3($**L1**$)_4](NO_3)_4$, **4a**.

Figure S41a: NOESY (500 MHz, DMSO-d_6, 300 K) for $[(NO_3)_2@Pd_3($**L1**$)_4](NO_3)_4$, **4a**.

Figure S41b: NOESY expansion (500 MHz, DMSO-d_6, 300 K) for $[(NO_3)_2@Pd_3($**L1**$)_4](NO_3)_4$, **4a**.

Figure S42a: ESI-MS, isotopic pattern for [**4a**-$2NO_3]^{2+}$ i) experimental and ii) theoretical.

Figure S42b: ESI-MS, isotopic pattern for [**4a**-$3NO_3]^{3+}$ i) experimental and ii) theoretical.

Figure S42c: ESI-MS, isotopic pattern for [**4a**-$4NO_3]^{4+}$ i) experimental and ii) theoretical.

Figure S43: Partial ^1H NMR spectra (400 MHz, DMSO-d_6, 300 K) for (i) ligand **L1**; (ii) a solution of $[Pd($**L1**$)_2](NO_3)_2$, **3a** after standing for 15 days.

Figure S44: Partial ^1H NMR spectra (400 MHz, DMSO-d_6, 300 K) for (i) ligand **L1**; (ii) a solution of $[Pd($**L1**$)_2](NO_3)_2$, **3a** after heating at 90 ºC 24 h (**3a** labelled in green and free ligand **L1** in black) (iii) pure $[Pd($**L1**$)_2](NO_3)_2$, **3a** for comparison.

Figure S45: Partial ^1H NMR spectra (400 MHz, DMSO-d_6, 300 K) for (i) complex $[Pd_1($**L1**$)_2](NO_3)_2$, **3a**; (ii) Monitoring formation of complex $[Pd_1($**L1**$)_2](NO_3)_2$, **3a** (labelled in green) and $[(NO_3)_2@Pd_3($**L1**$)_4](NO_3)_4$, **4a** (labelled in blue) by combining $Pd(NO_3)_2$ with **3a** and recorded after 10 min (iii) exclusive formation of $[(NO_3)_2@Pd_3($**L1**$)_4](NO_3)_4$, **4a** after 20 min.

Figure S46: Partial ^1H NMR spectra (400 MHz, DMSO-d_6, 300 K) for (i) complex $[Pd_1($**L1**$)_2](NO_3)_2$, **3a**; (ii) exclusive formation of $[(NO_3)_2@Pd_3($**L1**$)_4](NO_3)_4$, **4a** by heating a mixture of $Pd(NO_3)_2$ and **3a** at 90 °C.

Figure S47: Partial ^{1}H NMR spectra (400 MHz, DMSO-d_6, 300 K) for (i) ligand **L1**; (ii) $[(NO_3)_2@Pd_3($**L1**$)_4](NO_3)_4$, **4a** after standing for15 days.

Figure S48: Partial ^{1}H NMR spectra (400 MHz, DMSO-d_6, 300 K) for (i) ligand **L1**; (ii) a solution of $[(NO_3)_2@Pd_3($**L1**$)_4](NO_3)_4$, **4a** heated at 90 ºC for 5 days showing no change.

Figure S49: Partial ^{1}H NMR spectra (400 MHz, DMSO-d_6, 300 K) for (i) ligand **L1** ii) $[(NO_3)_2@Pd_3($**L1**$)_4](NO_3)_4$, **4a**; (iii) a mixture of pre-prepared $[(NO_3)_2@Pd_3($**L1**$)_4](NO_3)_4$, **4a** (labelled in blue) and ligand **L1** (iv) minor amount of $[Pd_1($**L1**$)_2](NO_3)_2$, **3a** (labelled in green) generated from a mixture of **4a** and **L1** after stirring for 20 min.

Figure S50: Partial ^{1}H NMR spectra (400 MHz, DMSO-d_6, 300 K) for (i) ligand **L1** ii) $[(NO_3)_2@Pd_3($**L1**$)_4](NO_3)_4$, **4a**; (iii) a mixture of pre-prepared $[(NO_3)_2@Pd_3($**L1**$)_4](NO_3)_4$, **4a** (labelled in blue) and ligand **L1** that generated minor amount of $[Pd_1($**L1**$)_2](NO_3)_2$, **3a** (labelled in green) after stirring at 90 °C for 5 min.

Figure S51: 400 MHz ^{1}H NMR spectra in DMSO-d_6 for *in situ* prepared (i) $[(NO_3)_2@Pd_3($**L1**$)_4](NO_3)_4$, **4a**; (ii) a sample prepared by addition of 1.5 equivalent of *n*-Bu_4NF to $[(NO_3)_2@Pd_3($**L1**$)_4](NO_3)_4$, **4a** (iii) $[(F)_2@Pd_3($**L1**$)_4](NO_3)_4$, **5a** obtained from a 3:1 combination of *n*-Bu_4NF with **4a**.

Figure S52: 400 MHz partial ^{1}H NMR spectra in DMSO-d_6 for *in situ* prepared (i) $[(NO_3)_2@Pd_3($**L1**$)_4](NO_3)_4$, **4a**; (ii) a sample prepared by addition of 1.5 equivalent of *n*-Bu_4NCl to $[(NO_3)_2@Pd_3($**L1**$)_4](NO_3)_4$, **4a** (iii) $[(Cl)_2@Pd_3($**L1**$)_4](NO_3)_4$, **6a** obtained from a 3:1 combination of *n*-Bu_4NCl with **4a**.

Figure S53: 400 MHz partial ^{1}H NMR spectra in DMSO-d_6 for *in situ* prepared (i) $[(NO_3)_2@Pd_3($**L1**$)_4](NO_3)_4$, **4a**; (ii) a sample prepared by addition of 1.5 equivalent of *n*-Bu_4NBr to $[(NO_3)_2@Pd_3($**L1**$)_4](NO_3)_4$, **4a** (iii) $[(Br)_2@Pd_3($**L1**$)_4](NO_3)_4$, **7a** obtained from a 3:1 combination of *n*-Bu_4NBr with **4a**.

Figure S54: Partial ^{1}H NMR for complex $[(NO_3)_2@Pd_3($**L1**$)_4](NO_3)_4$, **4a** and ii) a mixture of addition of TBAI **4a** (no changes).

Figure S55: Partial ^{1}H NMR spectra (400 MHz, DMSO-d_6, 300 K) for (i) $[Pd_1($**L1**$)_2](BF_4)_2$, **3b**; (ii) $[(NO_3)_2@Pd_3($**L1**$)_4](BF_4)_4$, **4b**; (iii) $[(F)_2@Pd_3($**L1**$)_4](BF_4)_4$, **5b**; (iv) $[(Cl)_2@Pd_3($**L1**$)_4](BF_4)_4$, **6b** and (v) $[(Br)_2@Pd_3($**L1**$)_4](BF_4)_4$, **7b**.

Figure S56: ^{1}H NMR (400 MHz, DMSO-d_6, 300 K) for **5a**.

Figure S57: ^{1}H NMR (400 MHz, DMSO-d_6, 300 K) for **6a**.

Figure S58: ^{13}C NMR (125 MHz, DMSO-d_6, 300 K) for **6a**.

Figure S59a: H-H COSY (500 MHz, DMSO-d_6, 300 K) for **6a.**

Figure S59b: H-H COSY expansion (500 MHz, DMSO-d_6, 300 K) for **6a**
Figure S60a: C-H COSY (500 MHz, DMSO-d_6, 300 K) for **6a**
Figure S60b: C-H COSY expansion (500 MHz, DMSO-d_6, 300 K) for **6a**
Figure S61a: NOESY (500 MHz, DMSO-d_6, 300 K) for **6a**
Figure S61b: NOESY expansion (500 MHz, DMSO-d_6, 300 K) for **6a**
Figure S62: ^{1}H NMR (500 MHz, DMSO-d_6, 300 K) for **7a**.
Figure S63: ^{13}C NMR (125 MHz, DMSO-d_6, 300 K) for **7a**.
Figure S64a: H-H COSY (500 MHz, DMSO-d_6, 300 K) for **7a**.
Figure S64b: H-H COSY expansion (500 MHz, DMSO-d_6, 300 K) for **7a**.
Figure S65a: C-H COSY (500 MHz, DMSO-d_6, 300 K) for **7a**.
Figure S65b: C-H COSY expansion (500 MHz, DMSO-d_6, 300 K) for **7a**.
Figure S66a: NOESY (500 MHz, DMSO-d_6, 300 K) for **7a**.
Figure S66b: NOESY expansion (500 MHz, DMSO-d_6, 300 K) for **7a**.
Figure S67: ESI-MS, isotopic pattern for [**6a**-2NO$_3$]$^{2+}$ i) experimental and ii) theoretical.
Figure S68a: ESI-MS, isotopic pattern for [**7a**-3NO$_3$]$^{3+}$i) experimental and ii) theoretical.
Figure S68b: ESI-MS, isotopic pattern for [**7a**-4NO$_3$]$^{4+}$ i) experimental and ii) theoretical.
Figure S69: ESI-MS, isotopic pattern for [(NO$_3$)(Br)@Pd$_3$(**L1**)$_4$](NO$_3$)$_4$ - 2NO$_3$]$^{2+}$ i.e. [**7a'**-2NO$_3$]$^{2+}$ i) experimental and ii) theoretical.
Table S1: ^{1}H NMR (DMSO-d$_6$) chemical shift of protons in ppm for the ligand **L1** and complexes **3a-7a**.

<u>List of Figures (S70-S71) and Table (S2-S3) related to Section S7:</u>

Figure S70. Energy minimization and electrostatic potential map of ligand **L1** (bottom) and ligand **L2** (bottom).
Figure S71. Energy minimized structures of (i) ligand **L1**, (ii) Pd^{2+}, (iii) [Pd(tmeda)]$^{2+}$, (iv) NO$_3^-$, (v) Cl$^-$, (vi) [Pd(tmeda)(**L1**)]$^{2+}$ i.e. complexed cation of **1a**, (vii) [Pd$_3$(tmeda)$_3$(**L**)$_2$]$^{6+}$, i.e. complexed cation of **2a**, (viii) [Pd(**L**)$_2$]$^{2+}$, i.e. complexed cation of **3a**, (ix) [(NO$_3$)$_2$@Pd$_3$(**L**)$_4$]$^{4+}$ i.e. complexed cation (with encapsulated anion) of **4a**, and (x) [(Cl)$_2$@Pd$_3$(**L**)$_4$]$^{4+}$, i.e. complexed cation (with encapsulated anion) of **5a**.
Table S2. DFT calculated electron density at the terminal and internal pyridine nitrogen atoms of ligands, **L1** and **L2**.
Table S3: ΔG and ΔH values of (i) ligand **L1**, (ii) [Pd(DMSO)$_2$(NO$_3$)$_2$], (iii) [Pd(tmeda)(NO$_3$)$_2$] (iv) NO$_3^-$, (v) Cl$^-$, (vi) [Pd(tmeda)(**L**)]$^{2+}$, (vii) [Pd$_3$(tmeda)$_3$(**L**)$_2$]$^{6+}$, (viii) [Pd(**L**)$_2$]$^{2+}$, (ix) [(NO$_3$)$_2$@Pd$_3$(**L**)$_4$]$^{4+}$, and (x) [(Cl)$_2$@Pd$_3$(**L**)$_4$]$^{4+}$ (xi) DMSO.

Section S1. Materials and Instruments

$PdCl_2$, $Pd(NO_3)_2$, tetra-*n*-butylammonium halides (fluoride, chloride, bromide and iodide) and tetra-*n*-butylammonium nitrate were obtained from Sigma-Aldrich. Triethylamine, $AgNO_3$, nicotinic acid, 4-dimethylaminopyridine, pyridine-3,5-dicarboxylic acid and common solvents were obtained from Spectrochem, India. Pyridine-3,5-diyldimethanol was prepared by reducing pyridine-3,5-dicarboxylic acid. Nicontinoylchloride hydrochloride was prepared from nicotinic acid. The deuterated solvents DMSO-d_6 and $CDCl_3$ were obtained either from Sigma-Aldrich or Cambridge Isotope Laboratories. Nuclear magnetic resonance (NMR) spectra were recorded in $CDCl_3$ or DMSO-d_6 at room temperature (rt) using Bruker AV400 and AV500 spectrometers at 400 and 500 MHz for ^{1}H NMR, COSY, NOESY and at 100 and 125 MHz for ^{13}C NMR, respectively. Chemical shifts are reported in parts per million (ppm) relative to residual solvent protons (7.26 ppm for $CDCl_3$ in ^{1}H NMR and 77.16 in ^{13}C NMR; 2.50 ppm for DMSO-d_6 in ^{1}H NMR and 39.43 in ^{13}C NMR). ESIMS spectra were recorded with Agilent Q-TOF and Micromass Q-TOF spectrometers.

Section S2: Numbering scheme of the compounds

Chart 1:

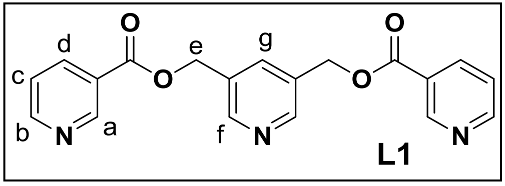

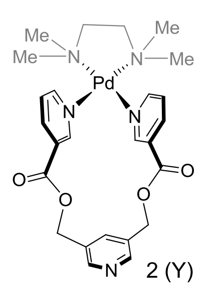	[Pd(tmeda)(**L1**)]$(Y)_2$	
	Y=NO_3	**1a**
	Y=BF_4	**1b**
	Y= ClO_4	**1c**
	Y=OTf	**1d**

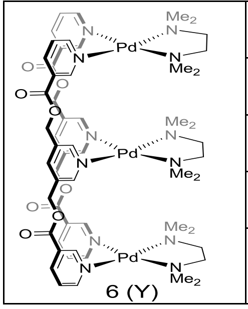	$[Pd_3(tmeda)_3($**L1**$)_2](Y)_6$	
	Y=NO_3	**2a**
	Y=BF_4	**2b**
	Y= ClO_4	**2c**
	Y=OTf	**2d**

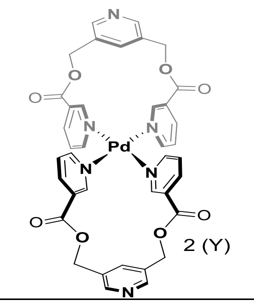	[Pd(**L1**)$_2$]$(Y)_2$	
	Y=NO_3	**3a**
	Y=BF_4	**3b**
	Y= ClO_4	**3c**
	Y=OTf	**3d**

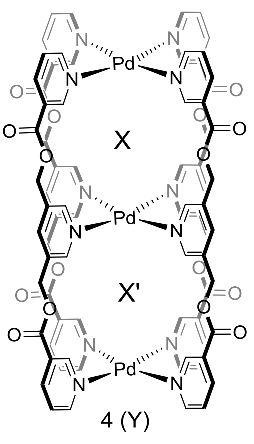	[(X)(X')@Pd_3(**L1**)$_4$]$(Y)_4$						
		X=NO_3 X'=NO_3	X=F X'=NO_3	X=F X'=F	X=Cl X'=NO_3	X=Cl X'=Cl	X=Br X'=NO_3	X=Br X'=Br
	Y=NO_3	**4a**	**5a'**	**5a**	**6a'**	**6a**	**7a'**	**7a**
	Y=BF_4	**4b**	**5b'**	**5b**	**6b'**	**6b**	**7b'**	**7b**

Section S3. Synthesis of [Pd(tmeda)(**L1**)]$(Y)_2$, **1b-1d**. (Y= BF_4, ClO_4 and OTf)

[Pd(tmeda)(L1)](BF_4)$_2$, 1b: To a solution of Pd(tmeda)$(BF_4)_2$ (11.9 mg, 0.03 mmol) in 3 mL of DMSO, ligand **L1** (10.5 mg, 0.03 mmol) was added. The reaction mixture was stirred at room temperature for 10 min to obtain a clear yellow solution. The resulting product was precipitated by addition of ethyl acetate (10 mL) and separated by centrifugation. The resulting off-white solid was washed with acetone (4 mL), dried under vacuum to afford the complex **1b** (19.2 mg, isolated yield 86%).

The ^{1}H NMR spectrum of the compound **1b** is closely comparable with the data of compound **1a**.

ESI-MS *m/z* : 658.14 [**1b** – $1BF_4]^{1+}$; 285.57 [**1b** – $2BF_4]^{2+}$

[Pd(tmeda)(L1)](ClO_4)$_2$, 1c: To a solution of Pd(tmeda)$(ClO_4)_2$ (12.7 mg, 0.03 mmol) in 3 mL of DMSO, ligand **L1** (10.5 mg, 0.03 mmol) was added. The reaction mixture was stirred at room temperature for 10 min to obtain a clear yellow solution. The resulting product was precipitated by addition of ethyl acetate (10 mL) and separated by centrifugation. The resulting off-white solid was washed with acetone (4 mL), dried under vacuum to afford the complex **1c** (20.1 mg, isolated yield 87%).

The ^{1}H NMR spectrum of the compound **1c** is closely comparable with the data of compound **1a**.

ESI-MS *m/z* : 672.09 [**1c** – $1ClO_4]^{1+}$; 285.57 [**1c** – $2ClO_4]^{2+}$

[Pd(tmeda)(L1)](OTf)$_2$, 1d: To a solution of Pd(tmeda)$(OTf)_2$ (15.6 mg, 0.03 mmol) in 3 mL of DMSO, ligand **L1** (10.5 mg, 0.03 mmol) was added. The reaction mixture was stirred at room temperature for 10 min to obtain a clear yellow solution. The resulting product was precipitated by addition of ethyl acetate (10 mL) and separated by centrifugation. The

resulting off-white solid was washed with acetone (4 mL), dried under vacuum to afford the complex **1d** (20.1 mg, isolated yield 77%).

The ^{1}H NMR spectrum of the compound **1d** is closely comparable with the data of compound **1a**.

ESI-MS *m/z* : 720.09 [**1d** – 1OTf]$^{1+}$; 285.57 [**1d** – 2OTf]$^{2+}$

Section S4. Synthesis of [Pd(**L1**)$_2$](Y)$_2$, **3b-3d**. (Y = BF_4, ClO_4 and OTf)

[Pd(L1)$_2$](BF$_4$)$_2$, 3b: To a solution of [Pd(DMSO)$_4$](BF_4)$_2$, prepared by stirring a mixture of PdI_2 (10.8 mg, 0.03 mmol) and $AgBF_4$ (11.7 mg, 0.06 mmol) in 3 mL of DMSO at 90 °C for 30 min, ligand **L1** (21.0 mg, 0.06 mmol) was added. The reaction mixture was kept stirring at room temperature for 10 min. Subsequently, addition of 10 mL of ethyl acetate to the resulting solution precipitated a white solid which was separated by centrifugation. The solid was washed with 2 × 10 mL of acetone and dried under vacuum to afford complex **3b** (16.5 mg, isolated yield 56%).

The ^{1}H NMR spectrum of the compound **3b** is closely comparable with the data of compound **3a**.

ESI-MS *m/z* : 891.12 [**3b** – 1BF_4]$^{1+}$; 402.05 [**3b** – 2BF_4]$^{2+}$

[Pd(L1)$_2$](ClO$_4$)$_2$, 3c: To a solution of [Pd(DMSO)$_4$](ClO_4)$_2$, prepared by stirring a mixture of PdI_2 (10.8 mg, 0.03 mmol) and $AgClO_4$ (12.4 mg, 0.06 mmol) in 3 mL of DMSO at 90 °C for 30 min, ligand **L1** (21.0 mg, 0.06 mmol) was added. The reaction mixture was kept stirring at room temperature for 10 min. Subsequently, addition of 10 mL of ethyl acetate to the resulting solution precipitated a white solid which was separated by centrifugation. The solid was washed with 2 × 10 mL of acetone and dried under vacuum to afford complex **3c** (20.2 mg, isolated yield 67%). The ^{1}H NMR spectrum of the compound **3c** is closely comparable with the data of compound **3a**.

[Pd(L1)$_2$](OTf)$_2$, 3d: To a solution of [Pd(DMSO)$_4$](OTf)$_2$, prepared by stirring a mixture of PdI_2 (10.8 mg, 0.03 mmol) and AgOTf (15.4 mg, 0.06 mmol) in 3 mL of DMSO at 90 °C for 30 min, ligand **L1** (20.1 mg, 0.06 mmol) was added. The reaction mixture was kept stirring at room temperature for 10 min. Subsequently, addition of 10 mL of ethyl acetate to the resulting solution precipitated a white solid which was separated by centrifugation. The solid was washed with 2 × 10 mL of acetone and dried under vacuum to afford complex **3d** (18.9 mg, isolated yield 57%). The ^{1}H NMR spectrum of the compound **3d** is closely comparable with the data of compound **3a**.

Section S5. Synthesis of [(X)$_2$@Pd$_3$(**L1**)$_4$](NO$_3$)$_4$, **5a-7a** (where X= F, Cl or Br)

[(F)$_2$@Pd$_3$(L1)$_4$](NO$_3$)$_4$, 5a: To a solution of compound [(NO$_3$)$_2$@Pd$_3$(L1)$_4$](NO$_3$)$_4$, **4a** in 0.4 mL of DMSO-d_6 (4.2 mg, 0.002 mmol), a solution of tetra-*n*-butylammonium fluoride (1.0 mg, 0.004 mmol) in DMSO-d_6 (0.2 mL) was added in portions (2 × 0.1 mL) and subsequently heated at 90 °C (the net volume for carrying out the reaction was 0.6 mL). The progress of the reaction was monitored by ^{1}H NMR spectroscopy at room temperature after the addition of each portion of the guest. The final spectrum showed a single set of peaks and a downfield shift of the pyridine α-H and β-H atoms of the pyridine rings. Since the complex was unstable it could not be isolated and was characterized by ^{1}H NMR spectroscopy. ^{1}H NMR (400 MHz, DMSO-d_6, 300 K): 10.88 (s, 1H, H_a), 10.05 (s, 1H, H_f), 9.82 (d, 1H, J = 4.6 Hz, H_b), 8.58 (d, 1H, J =7.0 Hz, H_d), 8.21 (s, 1H, H_g), 7.98 (s, 1H, H_c), 5.44 (s, 2H, H_e).

[(Cl)$_2$@Pd$_3$(L1)$_4$](NO$_3$)$_4$, 6a: Synthesis of **6a** was carried out in a similar fashion as for the synthesis of **5a** but instead of TBAF, TBACl (1.1 mg, 0.004 mmol) was used. ^{1}H NMR (500 MHz, DMSO-d_6, 300 K): 11.21(d, 1H, J = 1.8 Hz, H_a), 10.42 (s, 1H, H_f), 9.84-9.83 (m, 1H, H_b), 8.60-8.58 (m, 1H, H_d), 8.17 (s, 1H, H_g), 8.01-7.99 (m, 1 H, H_c), 5.47 (s, 2H, H_e). ^{13}C

NMR (125 MHz, DMSO-d_6, 300 K): 162.30, 154.97, 153.01, 148.59, 141.47, 133.92, 128.23, 127.71, 64.38.

ESI-MS *m/z* : 956.02 [**6a** – $2NO_3$]$^{2+}$

[(Br)$_2$@Pd$_3$(L1)$_4$](NO$_3$)$_4$, 7a: Synthesis of **7a** was carried out in a similar fashion as for the synthesis of **5a** but instead of TBAF, TBABr (1.3 mg, 0.004 mmol) was used. ^{1}H NMR (500 MHz, DMSO-d_6, 300 K): 11.37(s, 1H, H_a), 10.46 (s, 1H, H_f), 9.86 (d, 1H, J = 5.6 Hz, H_b), 8.56 (d, 1H, J = 8.0 Hz, H_d), 8.19 (s, 1H, H_g), 7.99-7.96 (m, 1H, H_c), 5.46 (s, 2H, H_e). ^{13}C NMR (125 MHz, DMSO-d_6, 300 K): 162.37, 155.00, 153.71, 149.81, 141.42, 137.53, 133.60, 128.23, 127.70, 64.69.

ESI-MS *m/z* : 645.99 [**7a** – $3NO_3$]$^{3+}$; 468.99 [**7a** – $4NO_3$]$^{4+}$

Section S6. Synthesis of [(NO$_3$)$_2$@Pd$_3$(**L1**)$_4$](BF$_4$)$_4$, **4b** and [(X)$_2$@Pd$_3$(**L1**)$_4$](BF$_4$)$_4$, **5b-7b** (where X= F, Cl or Br)

[(NO$_3$)$_2$@Pd$_3$(L1)$_4$](BF$_4$)$_4$, 4b: A solution of [Pd(DMSO)$_4$](BF$_4$)$_2$ was prepared in 0.6 mL of DMSO-d_6 by stirring a mixture of PdI_2 (2.2 mg, 0.006 mmol) and $AgBF_4$ (2.3 mg, 0.012 mmol) at 90 °C for 30 min. The precipitated AgI was separated by centrifugation and the clear solution was transferred using a syringe. The ligand **L1** (2.8 mg, 0.008 mmol) was added to the solution of Pd(BF$_4$)$_2$ (1.5 mg, 0.006 mmol) that was prepared in 0.6 mL of DMSO-d_6 followed by stirring the reaction mixture at room temperature for 10 min. To the in situ prepared [Pd(**L1**)$_2$](BF$_4$)$_2$, **3b**, TBANO$_3$ (1.2 mg, 0.004 mmol) solution prepared in DMSO-d_6 was added and the mixture heated at 90 °C for 5 min. Based on ^{1}H NMR analysis it was clear that the mononuclear complex [Pd(**L1**)$_2$](BF$_4$)$_2$, **3b** has converted to [(NO$_3$)$_2$@Pd$_3$(**L1**)$_4$](BF$_4$)$_4$, **4b**.

[(F)$_2$@Pd$_3$(L1)$_4$](BF$_4$)$_4$, 5b: A similar procedure was followed as for the synthesis of **4b** but instead of TBANO$_3$, TBAF (1.0 mg, 0.004 mmol) was used. Based on ^{1}H NMR analysis it

was clear that the mononuclear complex [Pd(**L1**)$_2$](BF_4)$_2$, **3b** has converted to [(Cl)$_2$@Pd$_3$(**L1**)$_4$](BF_4)$_4$, **5b**.

[(Cl)$_2$@Pd$_3$(L1)$_4$](BF_4)$_4$, 6b: A similar procedure was followed as for the synthesis of **4b** but instead of TBANO$_3$, TBACl (1.1 mg, 0.004 mmol) was used. Based on ^{1}H NMR analysis it was clear that the mononuclear complex [Pd(**L1**)$_2$](BF_4)$_2$, **3b** has converted to [(Cl)$_2$@Pd$_3$(**L1**)$_4$](BF_4)$_4$, **6b**.

[(Br)$_2$@Pd$_3$(L1)$_4$](BF_4)$_4$, 7b: A similar procedure was followed as for the synthesis of **4b** but instead of TBANO$_3$, TBABr (1.3 mg, 0.004 mmol) was used. Based on ^{1}H NMR analysis it was clear that the mononuclear complex [Pd(**L1**)$_2$](BF_4)$_2$, **3b** has converted to [(Br)$_2$@Pd$_3$(**L1**)$_4$](BF_4)$_4$, **7b**.

Section S7. Figures **S1**–**S69**) depicting NMR spectra and ESIMS of the ligands, complexes, and anion encapsulated complexes. NMR spectra to support dynamic behaviours of the complexes.

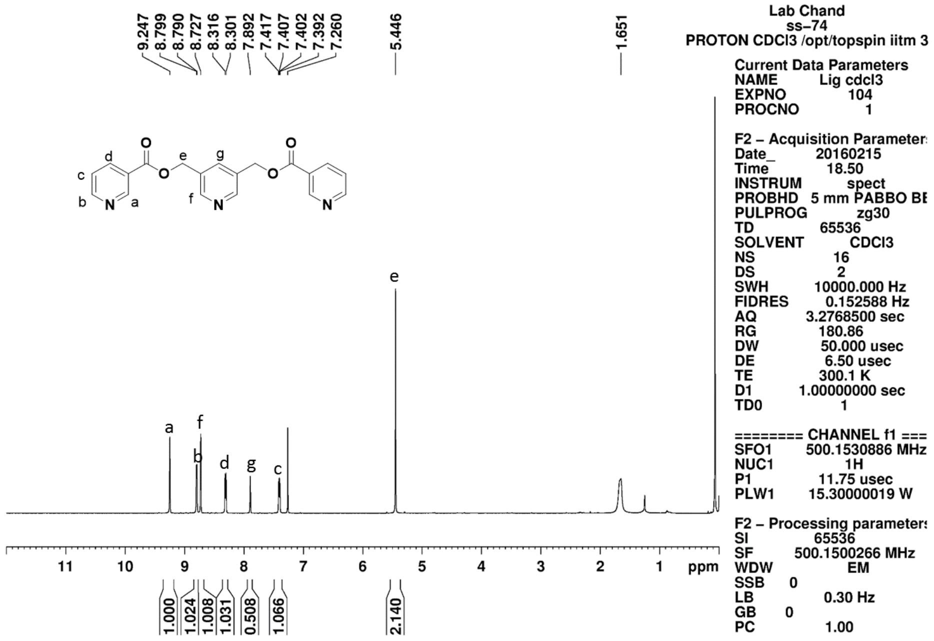

Figure S1: ^{1}H NMR (400 MHz, CDCl$_3$, 300 K) for **L1**.

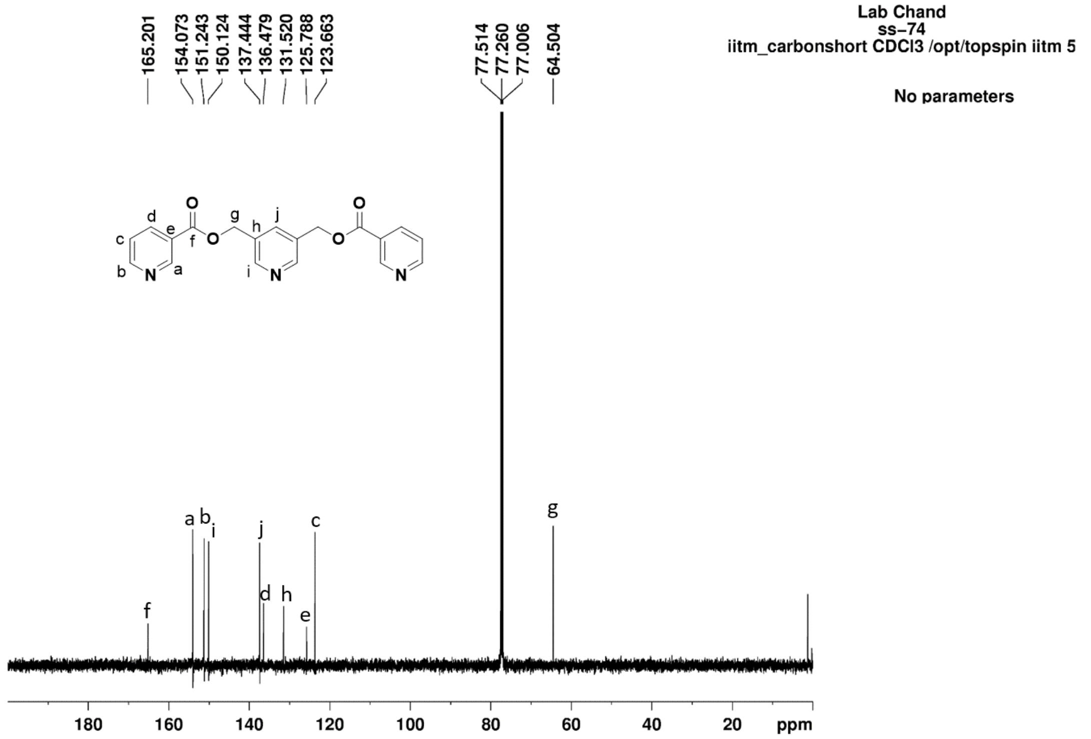

Figure S2: ^{13}C NMR (125 MHz, $CDCl_3$, 300 K) for **L1**.

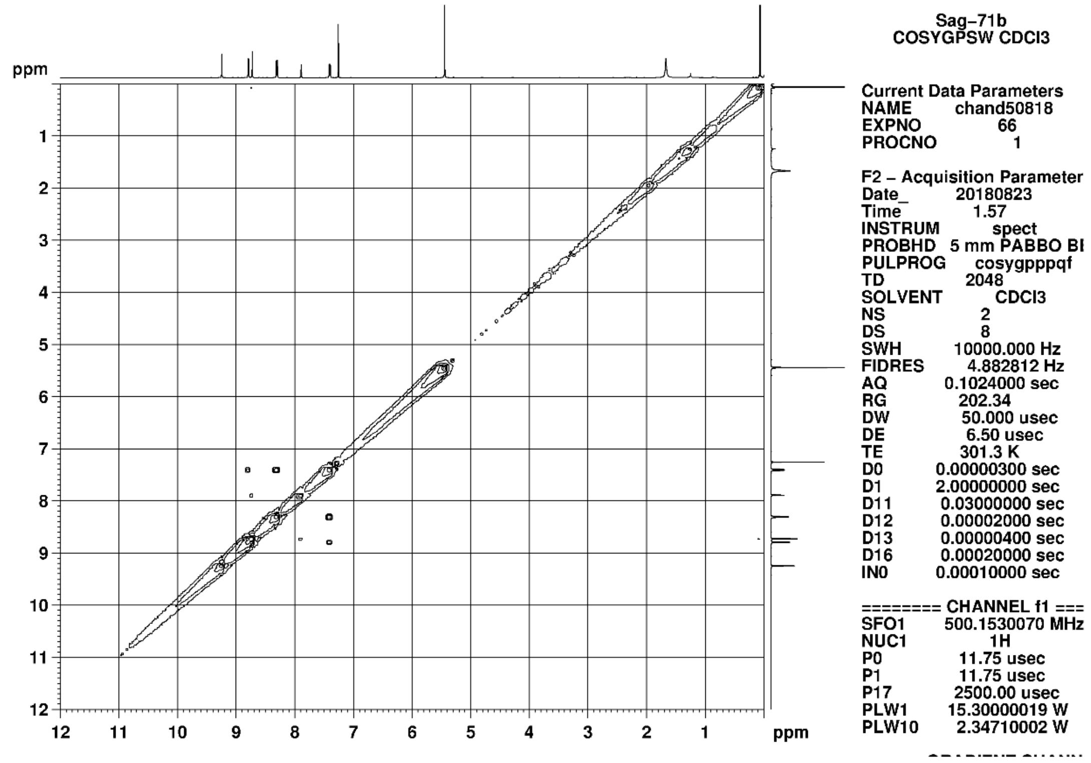

Figure S3a: H-H COSY (500 MHz, $CDCl_3$, 300 K) for **L1**.

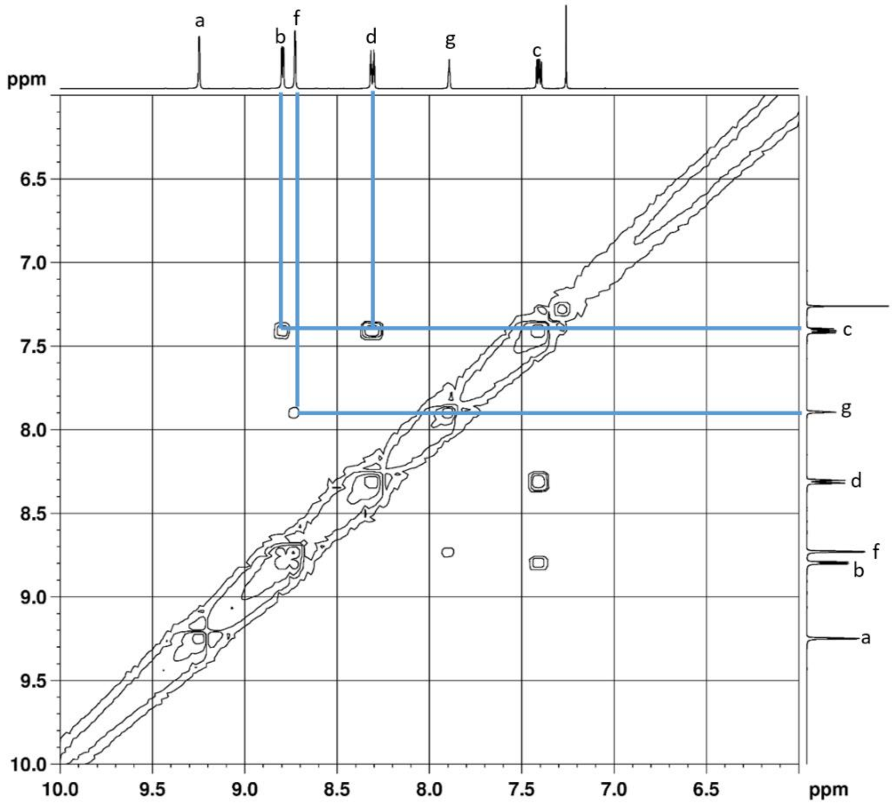

Figure S3b: H-H COSY expansion (500 MHz, $CDCl_3$, 300 K) for **L1**.

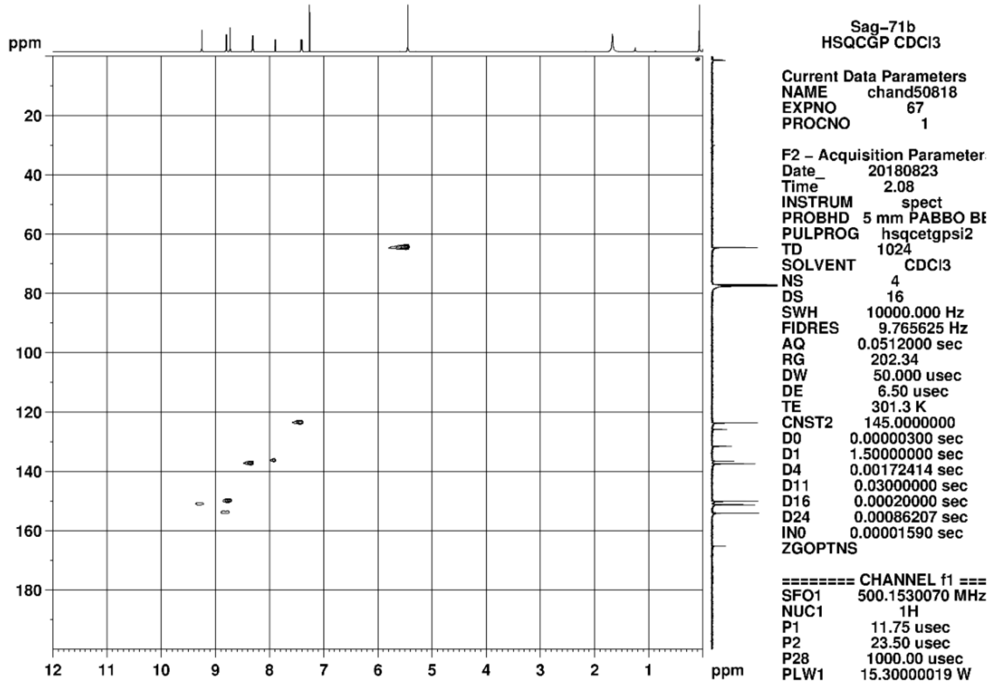

Figure S4a: C-H COSY (500 MHz, $CDCl_3$, 300 K) for **L1**.

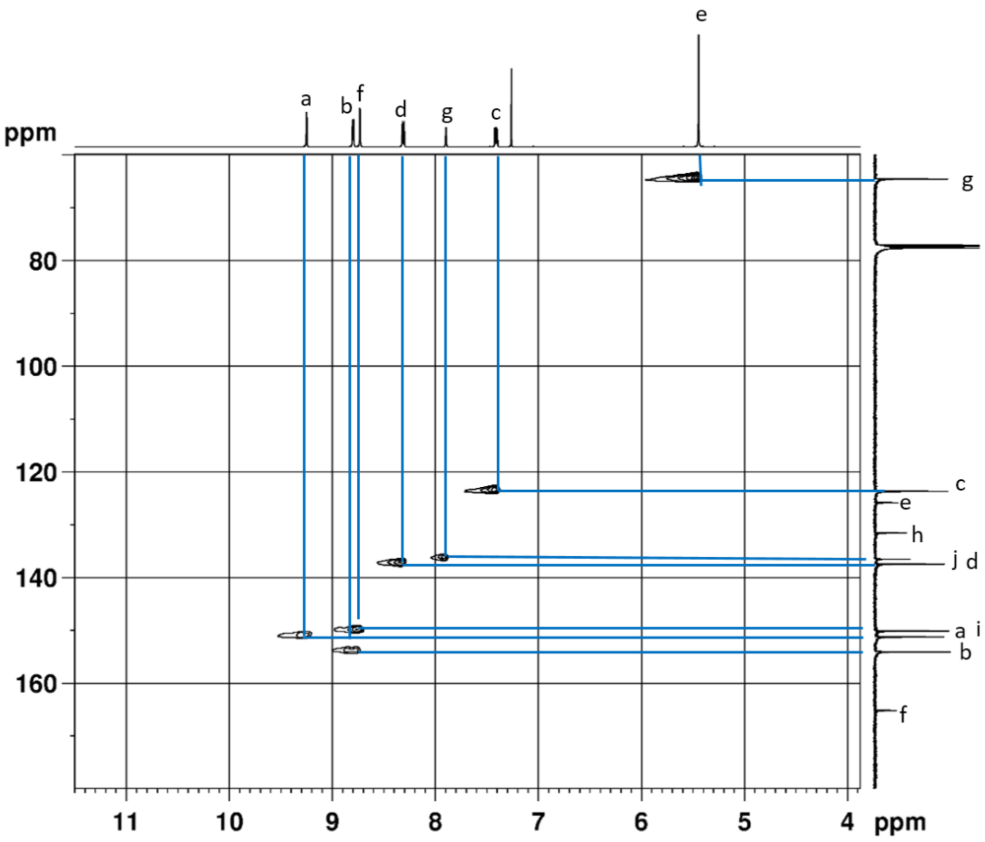

Figure S4b: C-H COSY expansion (500 MHz, $CDCl_3$, 300 K) for **L1**.

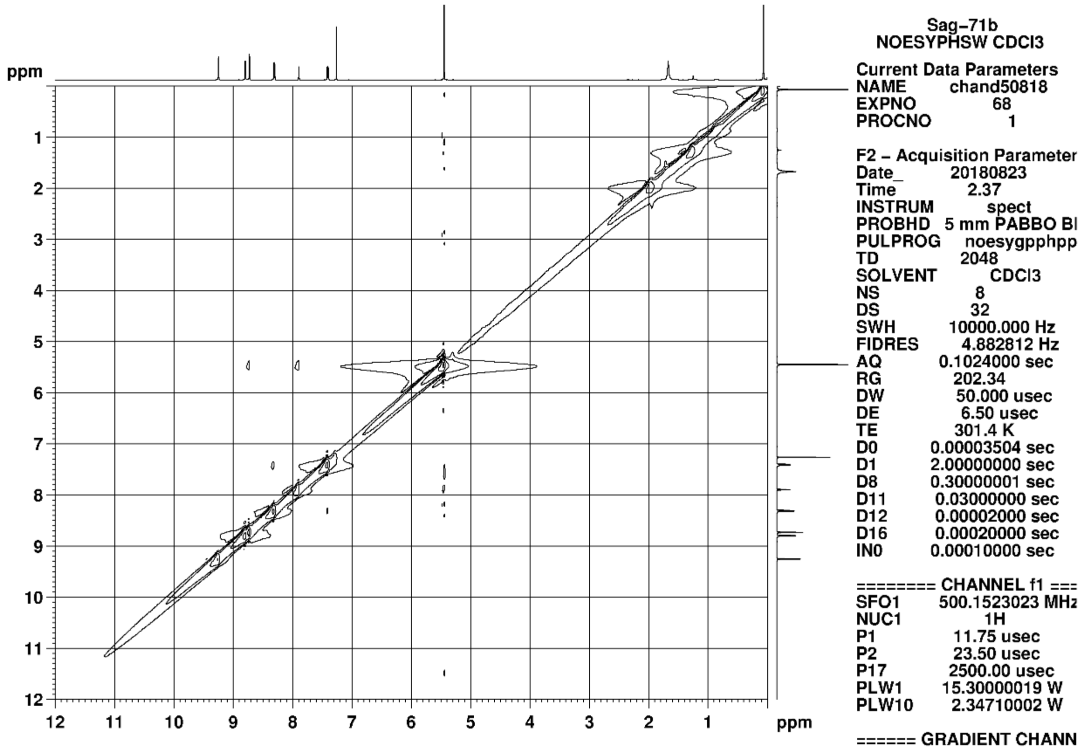

Figure S5a: NOESY (500 MHz, $CDCl_3$, 300 K) for **L1**.

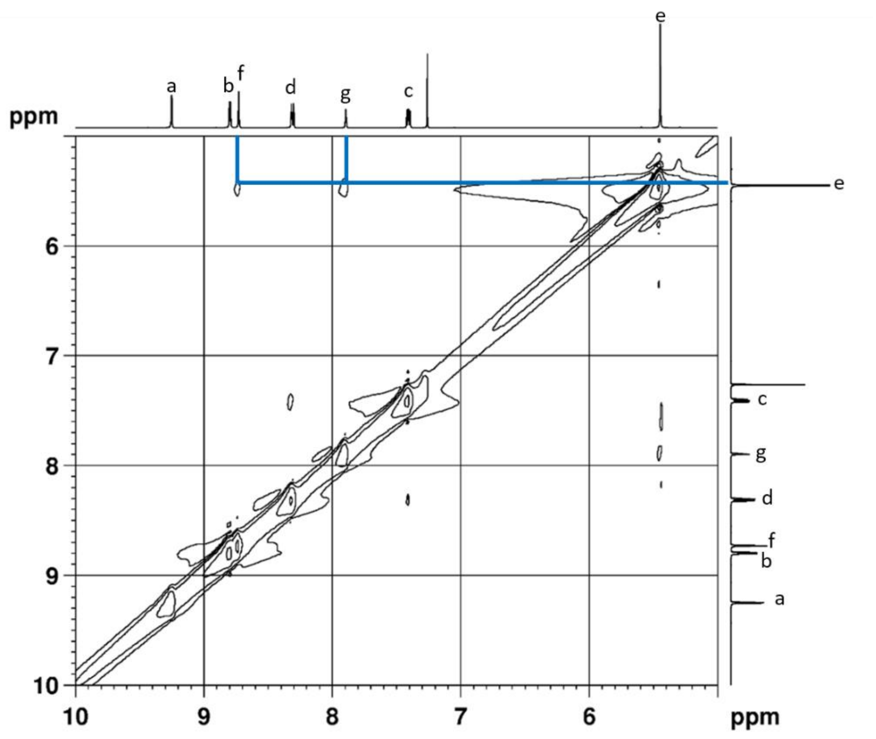

Figure S5b: NOESY expansion (500 MHz, $CDCl_3$, 300 K) for **L1**.

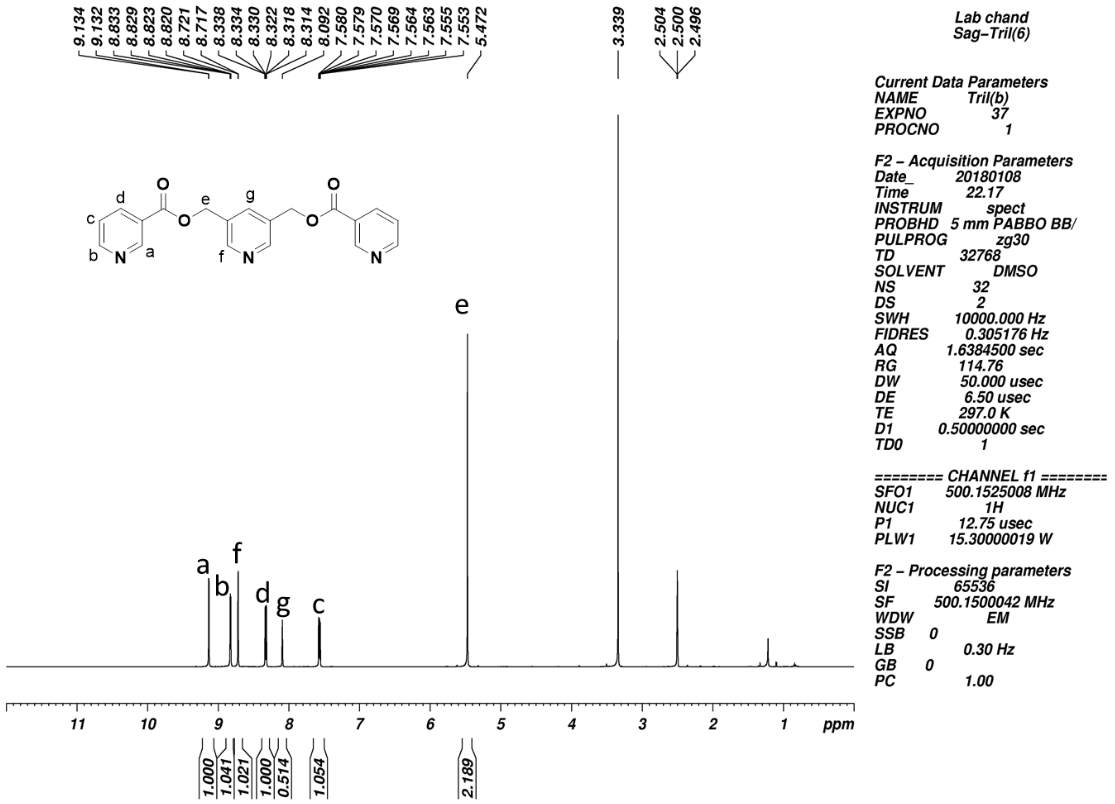

Figure S6: ^{1}H NMR (500 MHz, DMSO-d_6, 300 K) for **L1**.

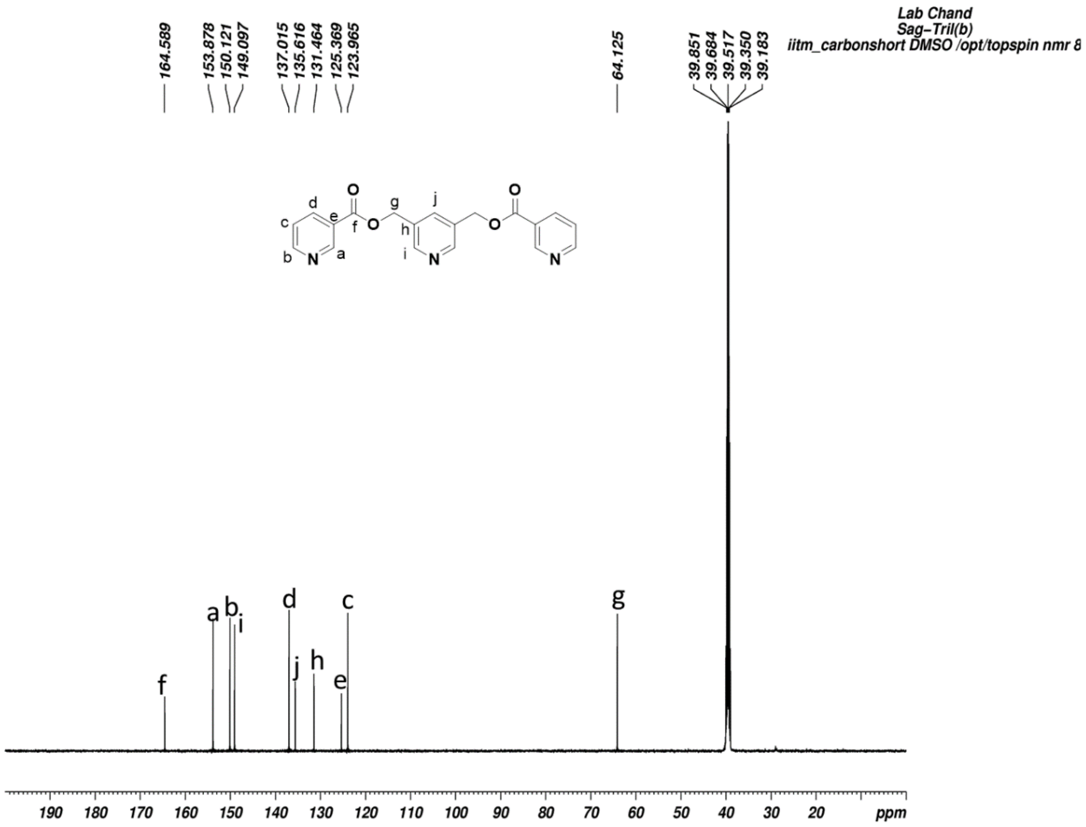

Figure S7: ^{13}C NMR (125 MHz, DMSO-d_6, 300 K) for **L1**.

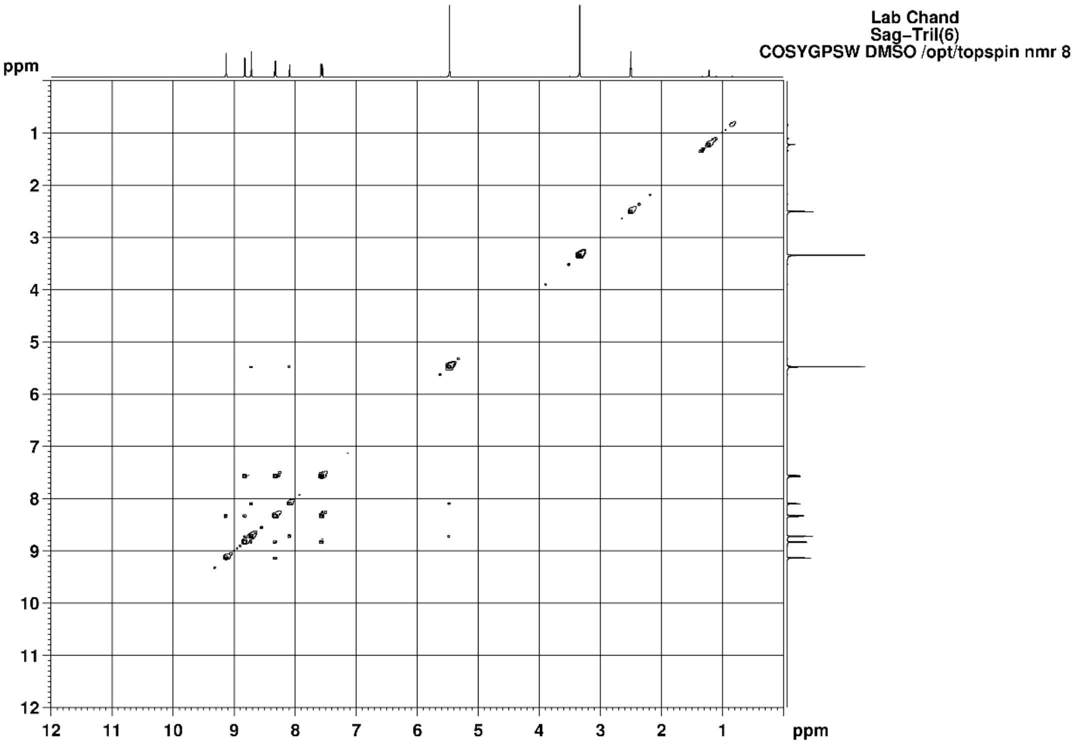

Figure S8a: H-H COSY (500 MHz, DMSO-d_6, 300 K) for **L1**.

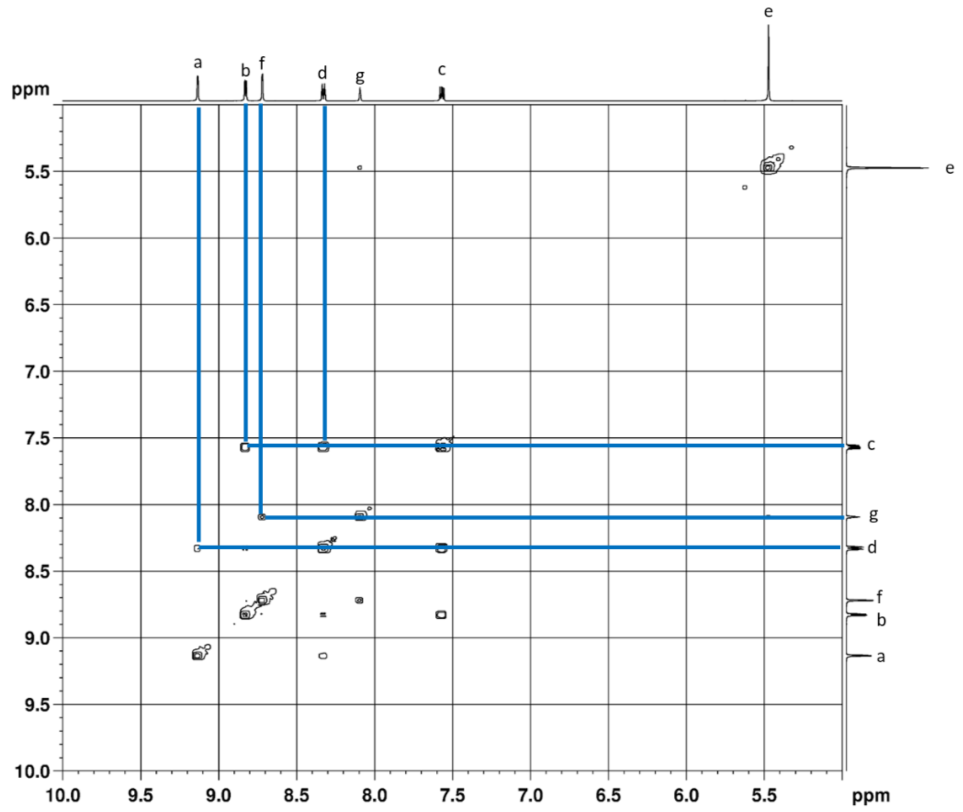

Figure S8b: H-H COSY expansion (500 MHz, DMSO-d_6, 300 K) for **L1**.

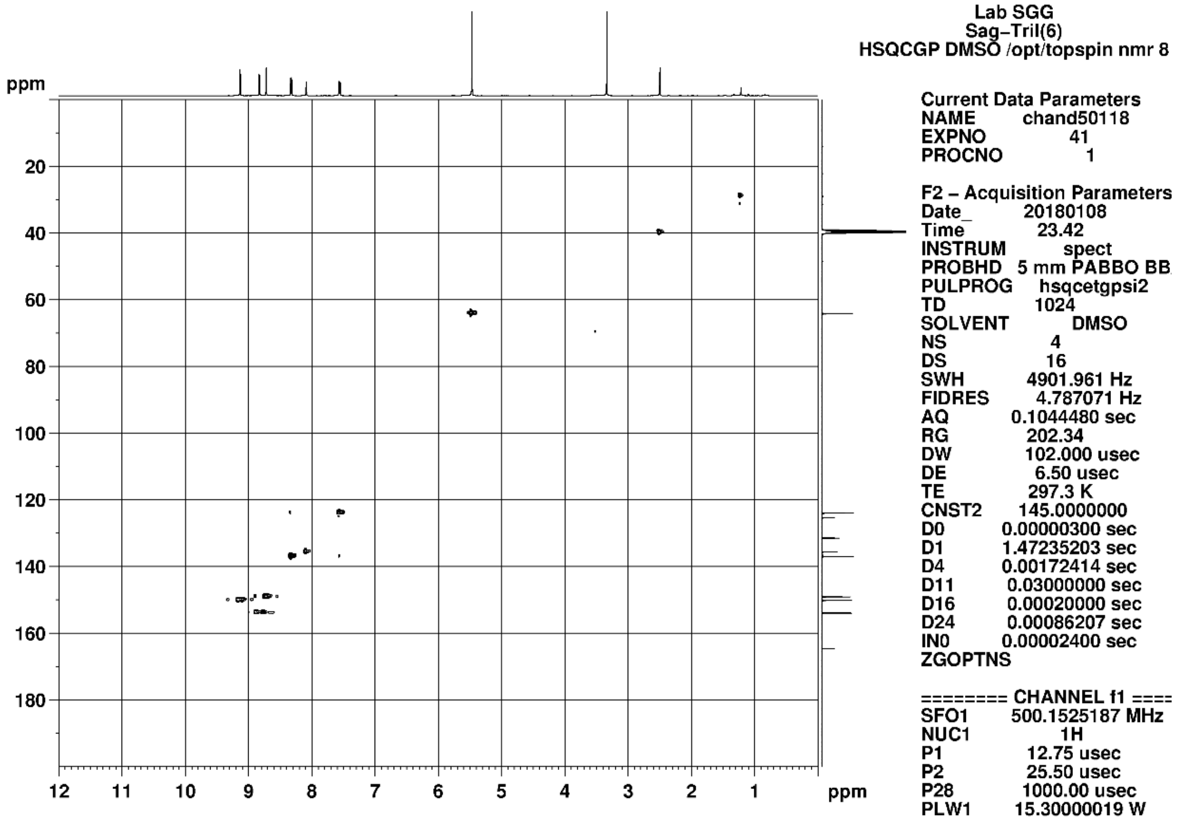

Figure S9a: C-H COSY (500 MHz, DMSO-d_6, 300 K) for **L1**.

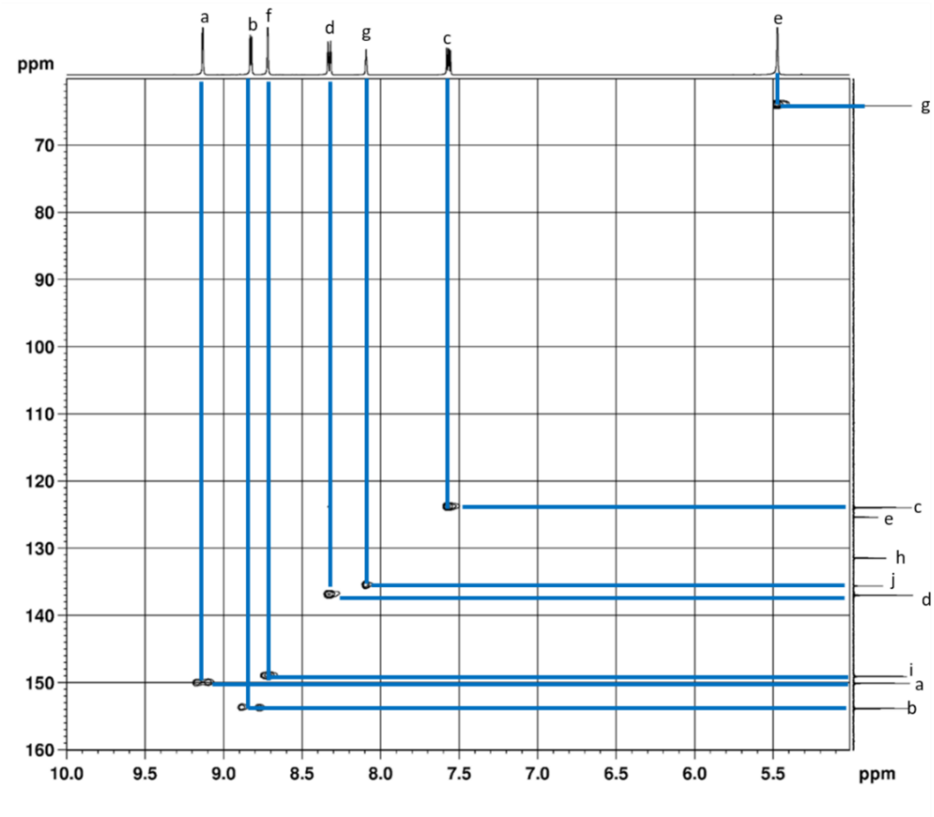

Figure S9b: C-H COSY expansion (500 MHz, DMSO-d_6, 300 K) for **L1**.

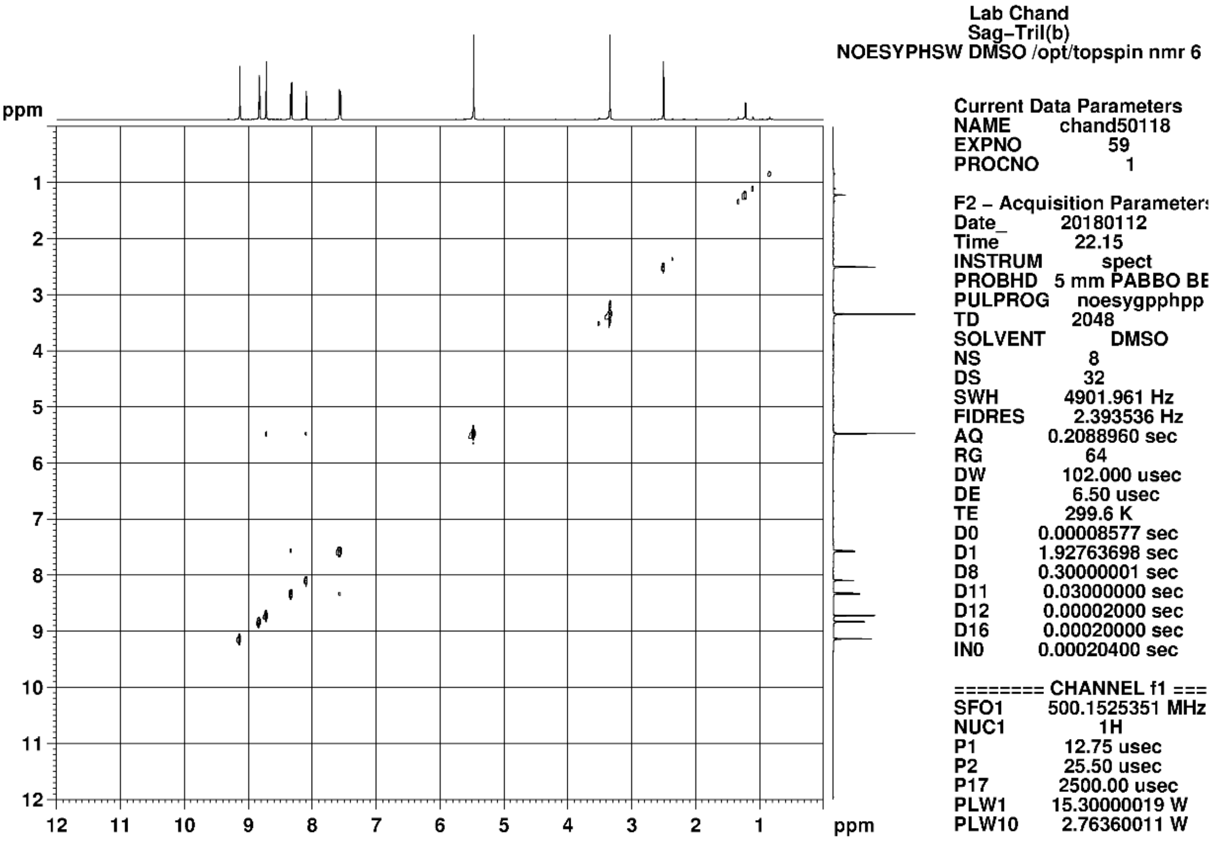

Figure S10a: NOESY (400 MHz, DMSO-d_6, 300 K) for **L1**.

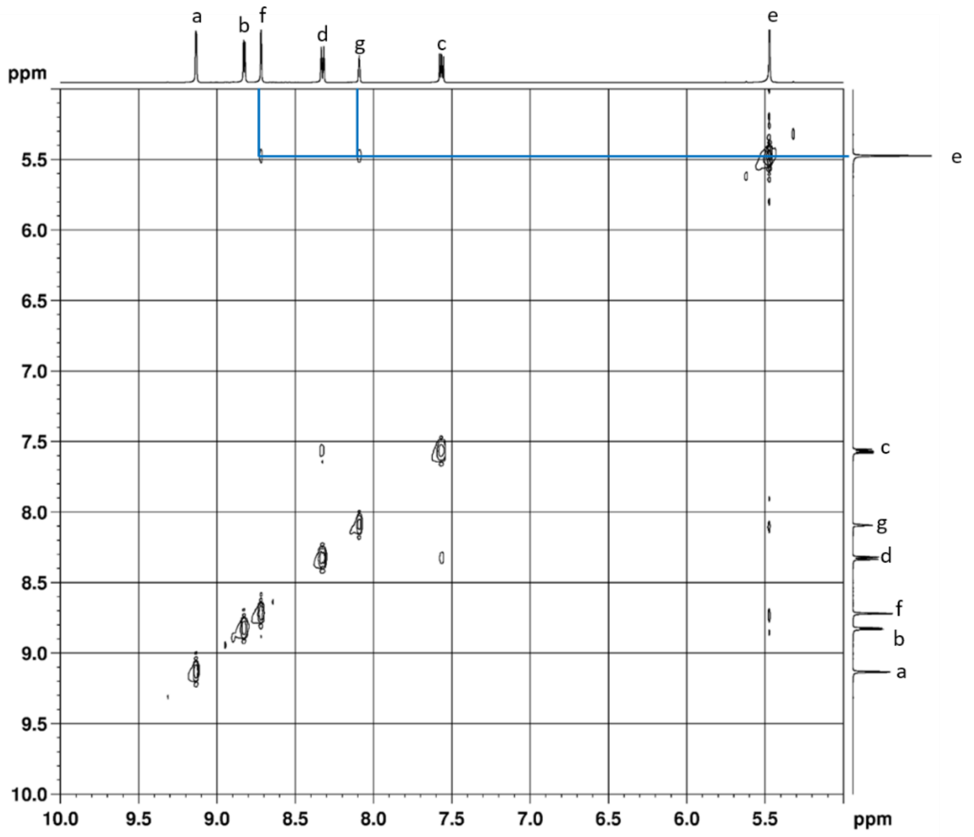

Figure S10b: NOESY expansion (500 MHz, DMSO-d_6, 300 K) for **L1**.

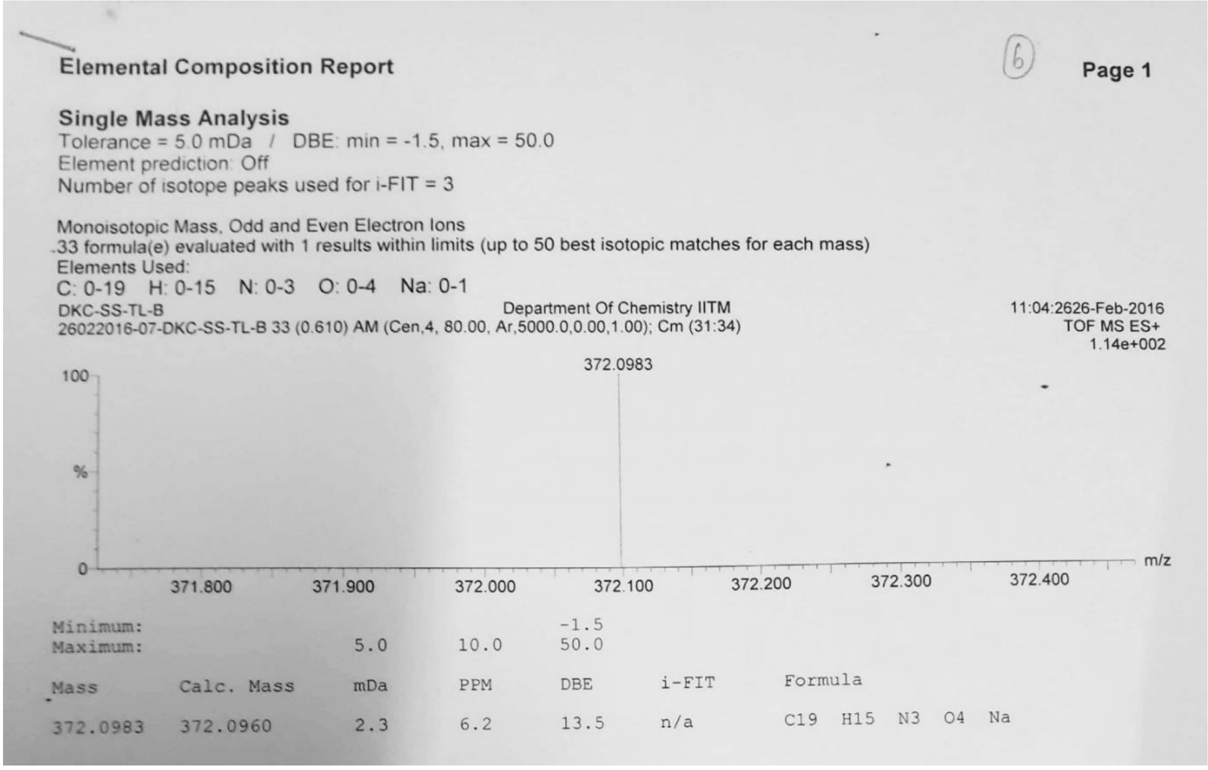

Figure S11: ESI-MS for **L1** showing $[M + Na]^+$.

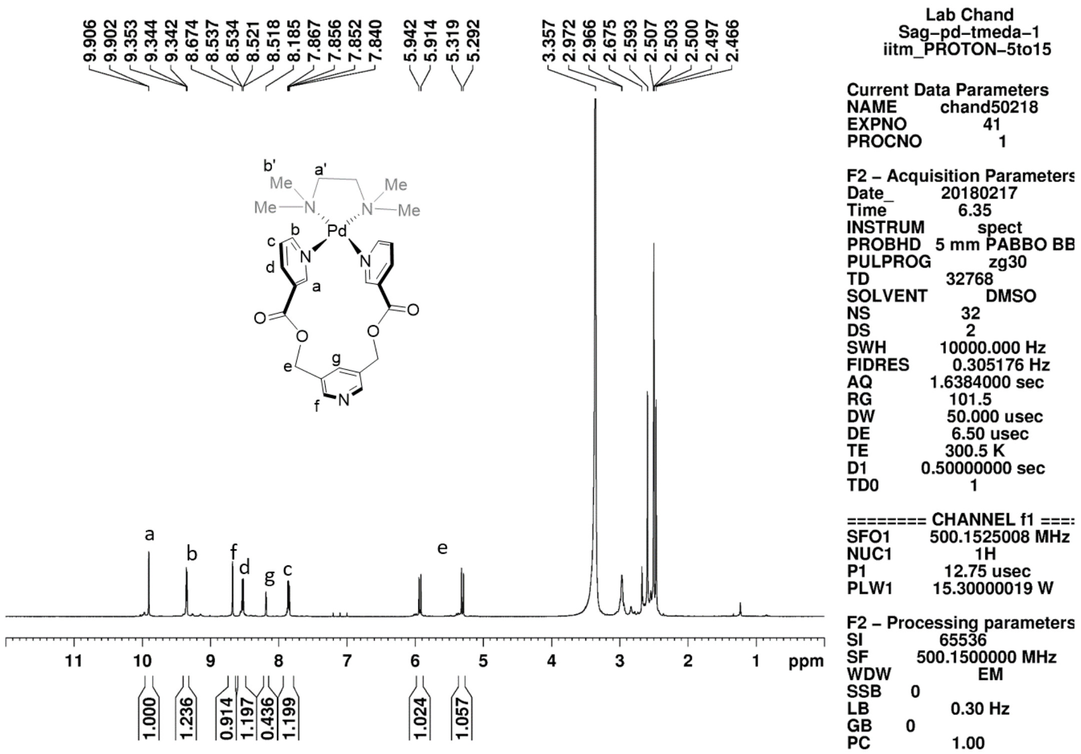

Figure S12: ^{1}H NMR (500 MHz, DMSO-d_6, 300 K) for [Pd(tmeda)(**L1**)](NO_3)$_2$, **1a**.

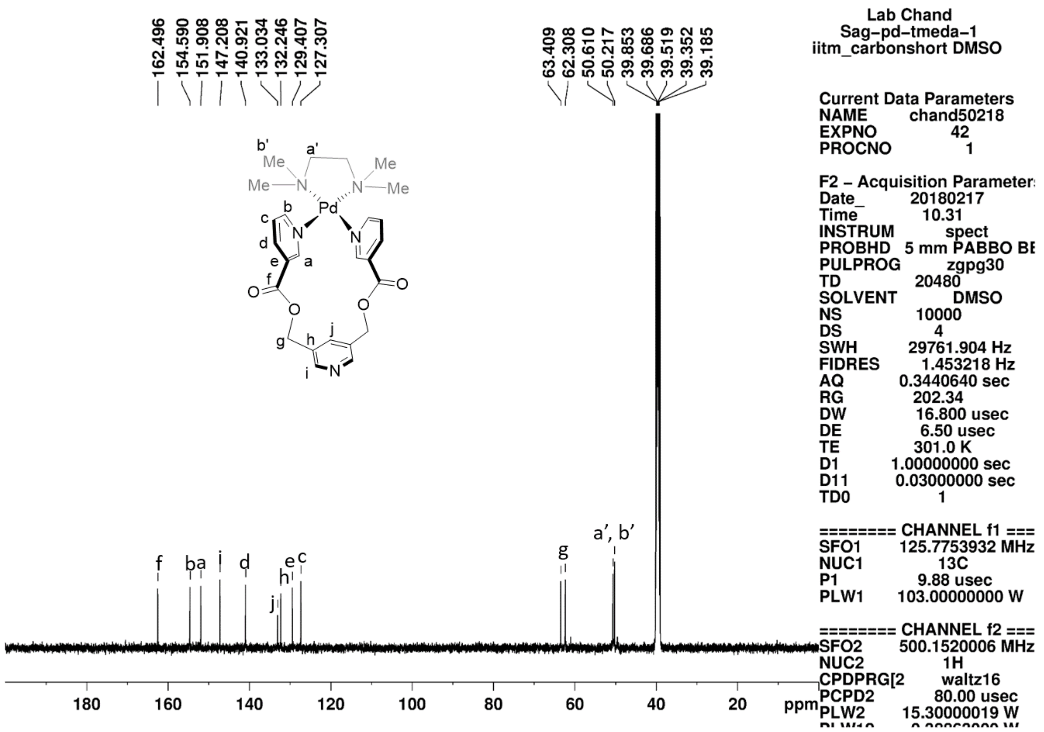

Figure S13: ^{13}C NMR (125 MHz, DMSO-d_6, 300 K) for [Pd(tmeda)(**L1**)](NO_3)$_2$, **1a**.

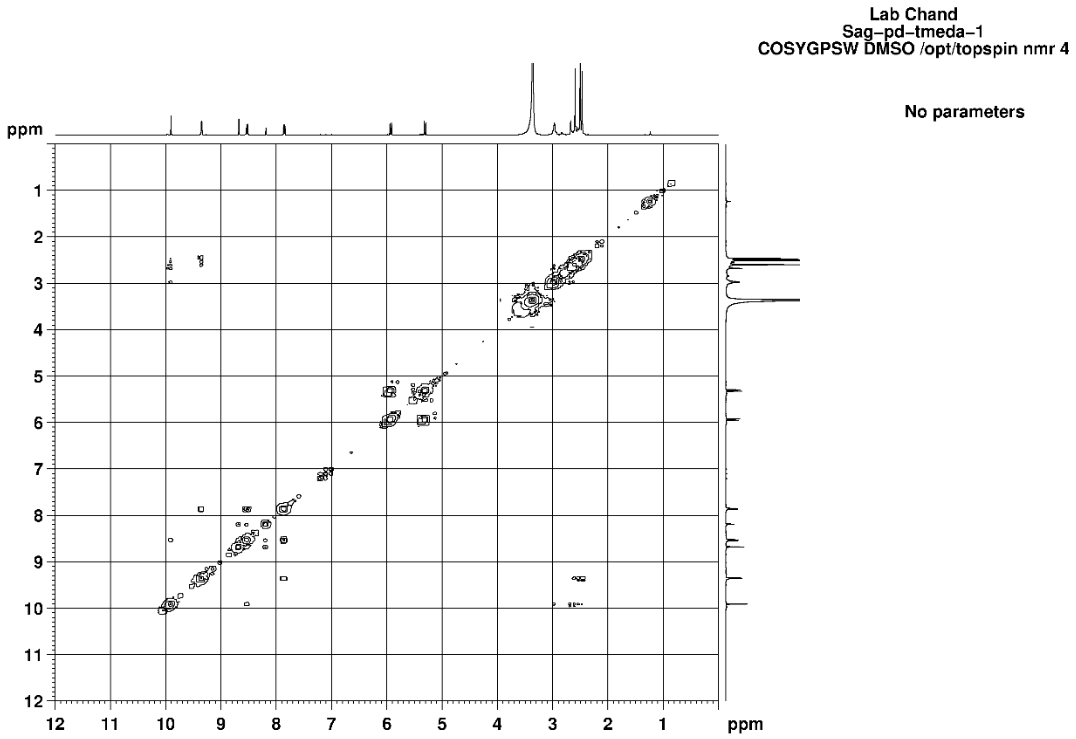

Figure S14a: H-H COSY (500 MHz, DMSO-d_6, 300 K) for [Pd(tmeda)(**L1**)]$(NO_3)_2$, **1a**.

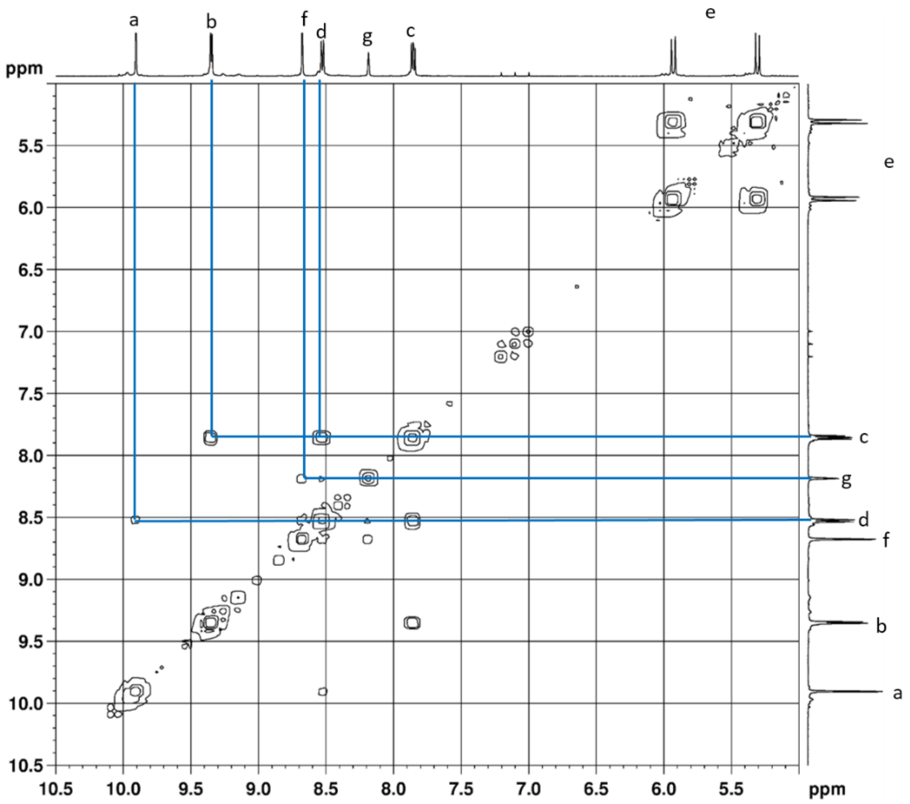

Figure S14b : H-H COSY expansion (500 MHz, DMSO-d_6, 300 K) for [Pd(tmeda)(**L1**)]$(NO_3)_2$, **1a**.

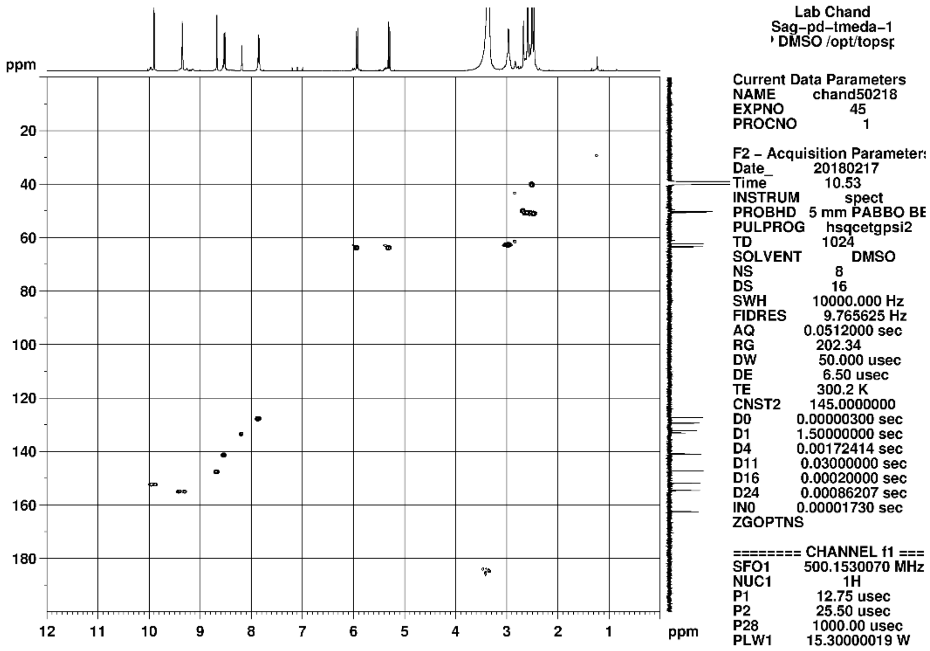

Figure S15a: C-H COSY (500 MHz, DMSO-d_6, 300 K) for [Pd(tmeda)(**L1**)](NO_3)$_2$, **1a**.

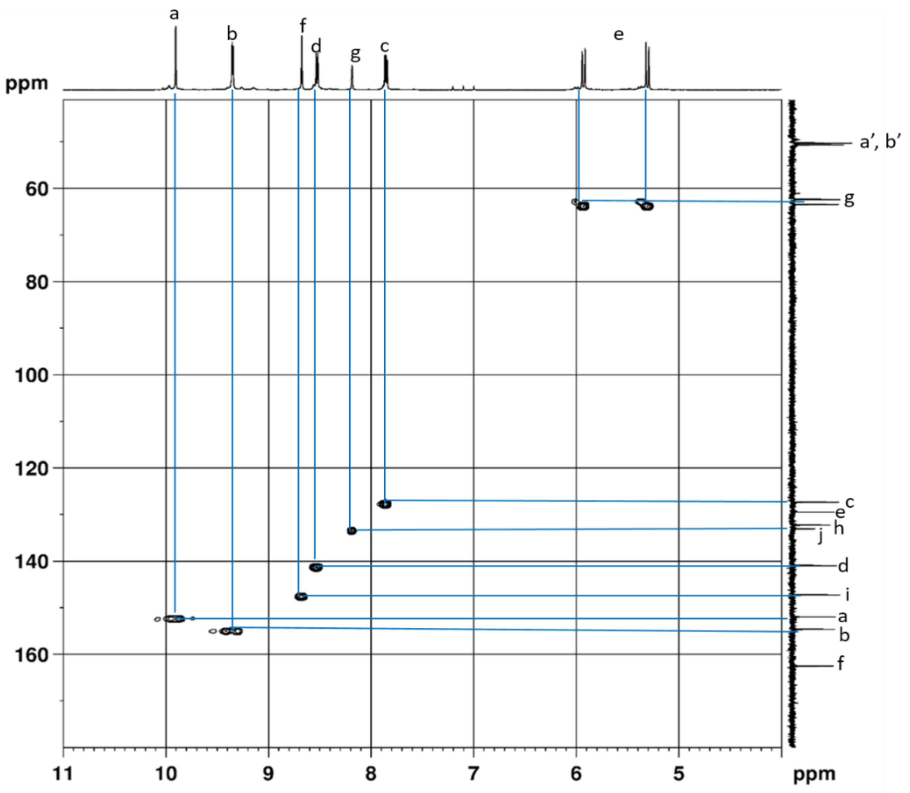

Figure S15b: C-H COSY expansion (500 MHz, DMSO-d_6, 300 K) for [Pd(tmeda)(**L1**)](NO_3)$_2$, **1a**.

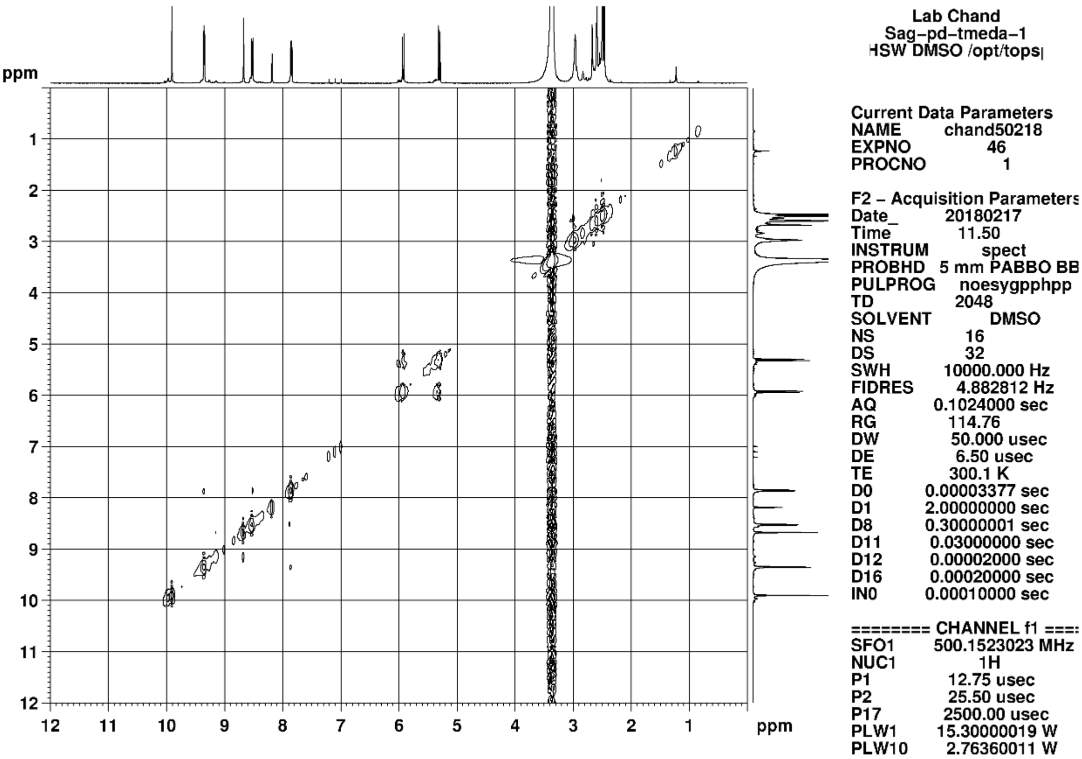

Figure S16a: NOESY (500 MHz, DMSO-d_6, 300 K) for [Pd(tmeda)(**L1**)]$(NO_3)_2$, **1a**.

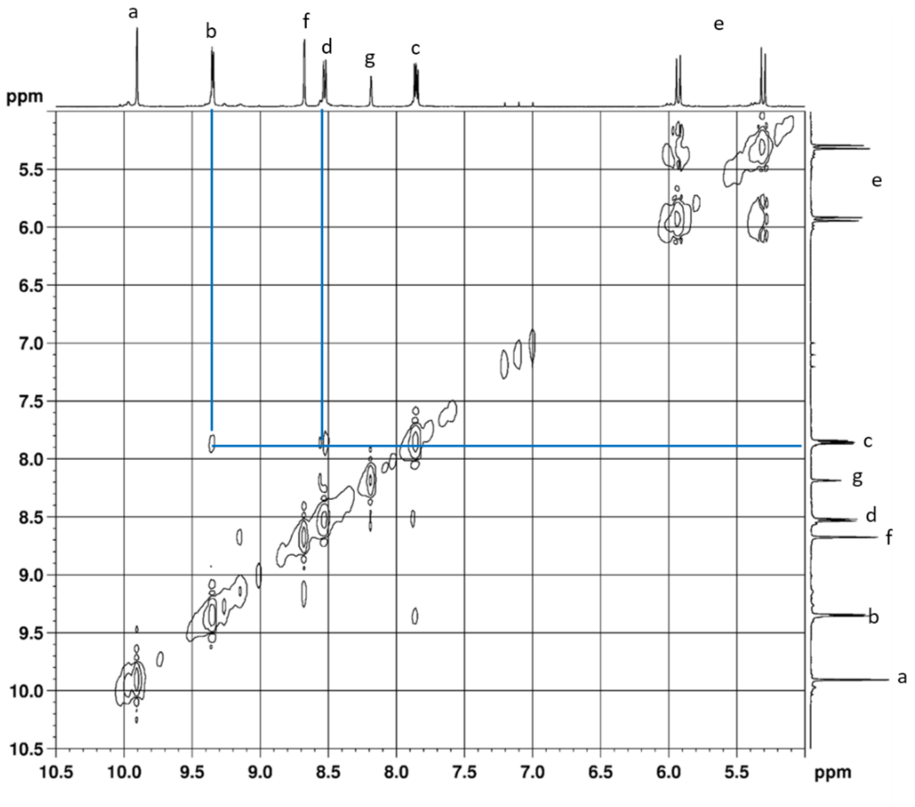

Figure S16b: NOESY expansion (500 MHz, DMSO-d_6, 300 K) for [Pd(tmeda)(**L1**)]$(NO_3)_2$, **1a**.

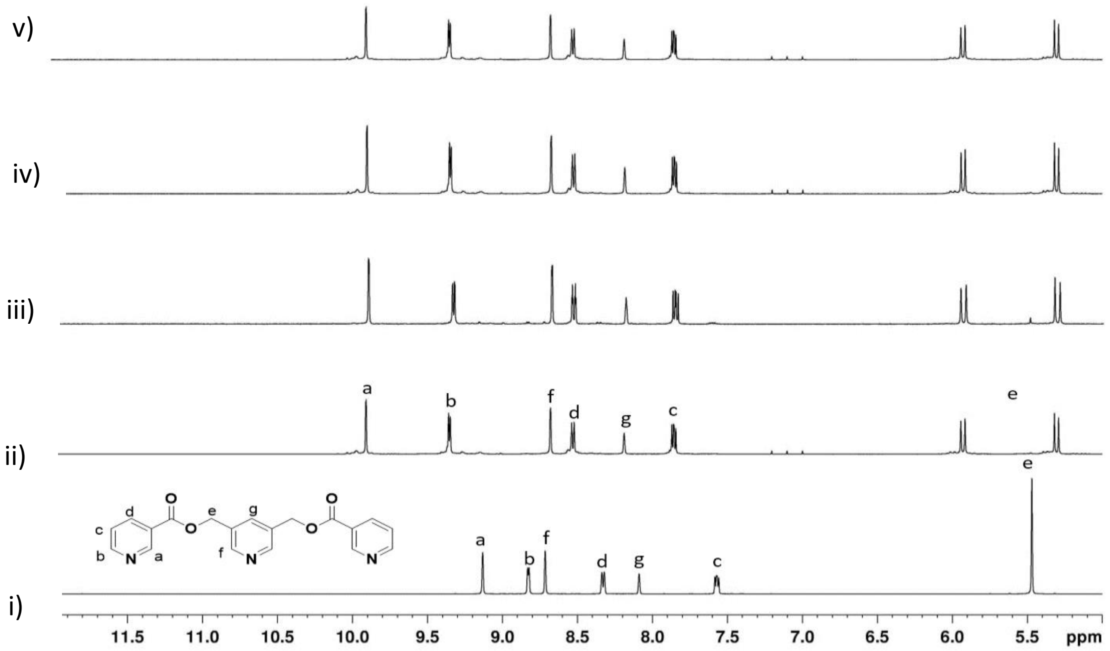

Figure S17: Partial ^{1}H NMR spectra (400 MHz, DMSO-d_6, 300 K) for (i) ligand **L1**; (ii)[Pd(tmeda)(**L1**)]$(NO_3)_2$**, 1a**; (iii) [Pd(tmeda)(**L1**)]$(BF_4)_2$**, 1b**;(iv) [Pd(tmeda)(**L1**)]$(ClO_4)_2$**, 1c**; and (v) [Pd(tmeda)(**L1**)]$(OTf)_2$**, 1d**.

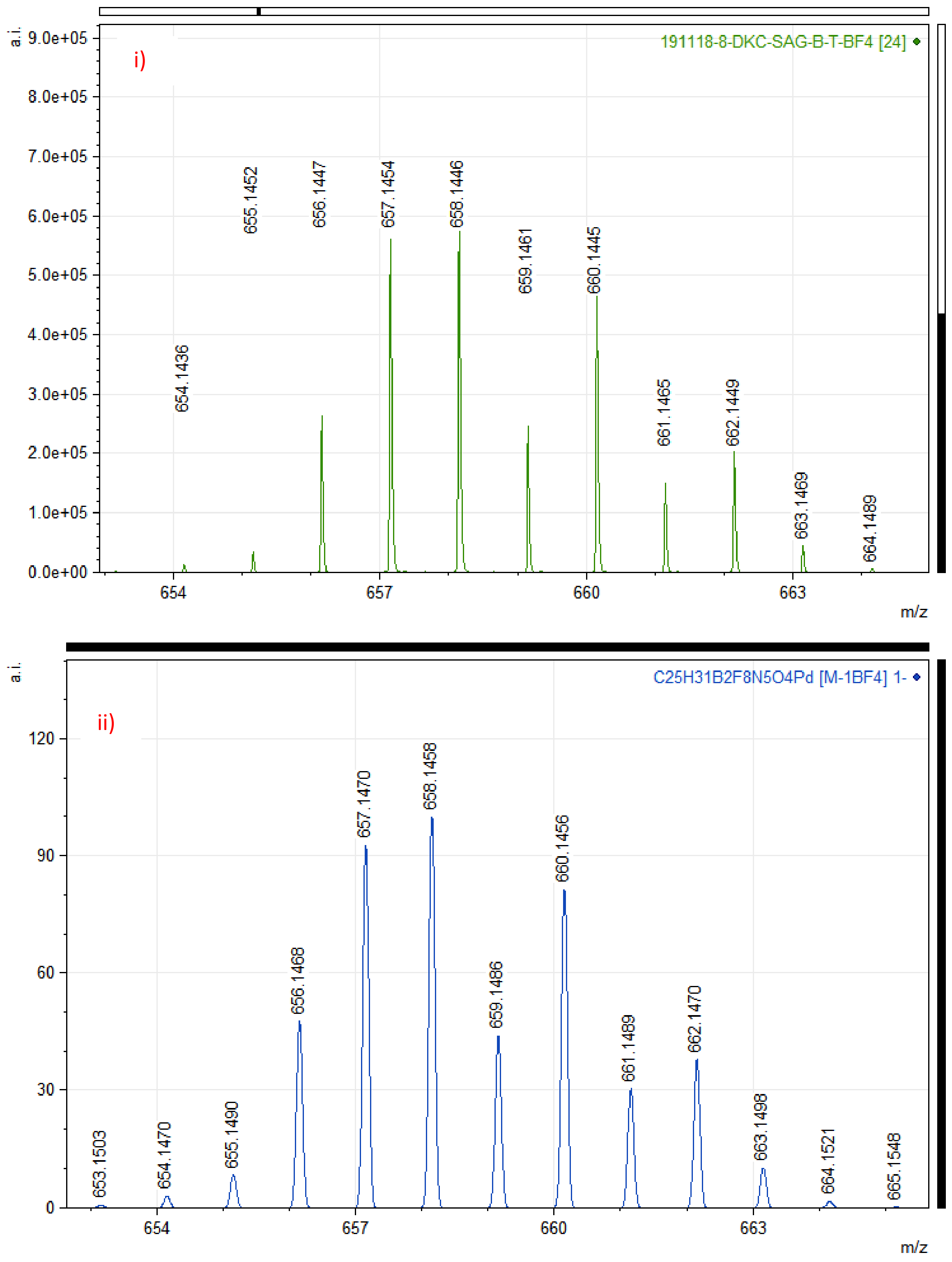

Figure S18a: ESIMS, isotopic pattern for [**1b** − BF_4]$^+$ i) experimental and ii) theoretical.

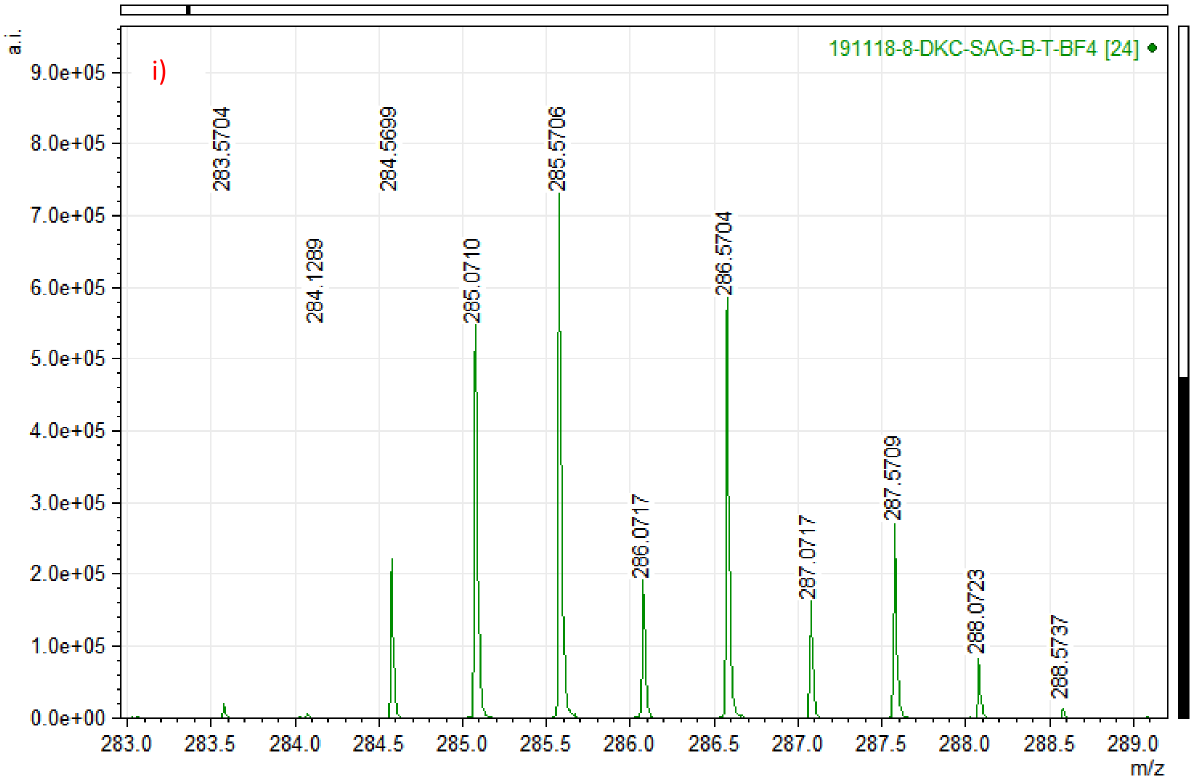

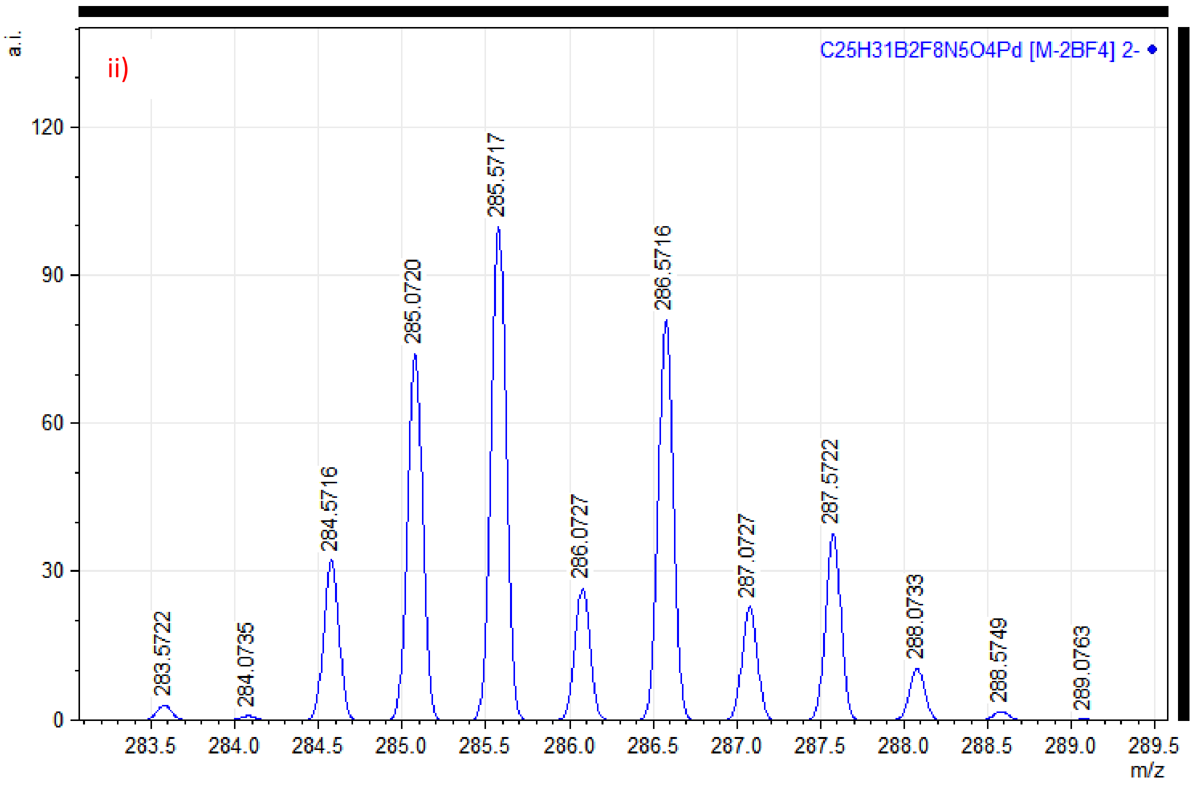

Figure S18b: ESI MS, isotopic pattern for [**1b** − $2BF_4]^{2+}$ i) experimental and ii) theoretical.

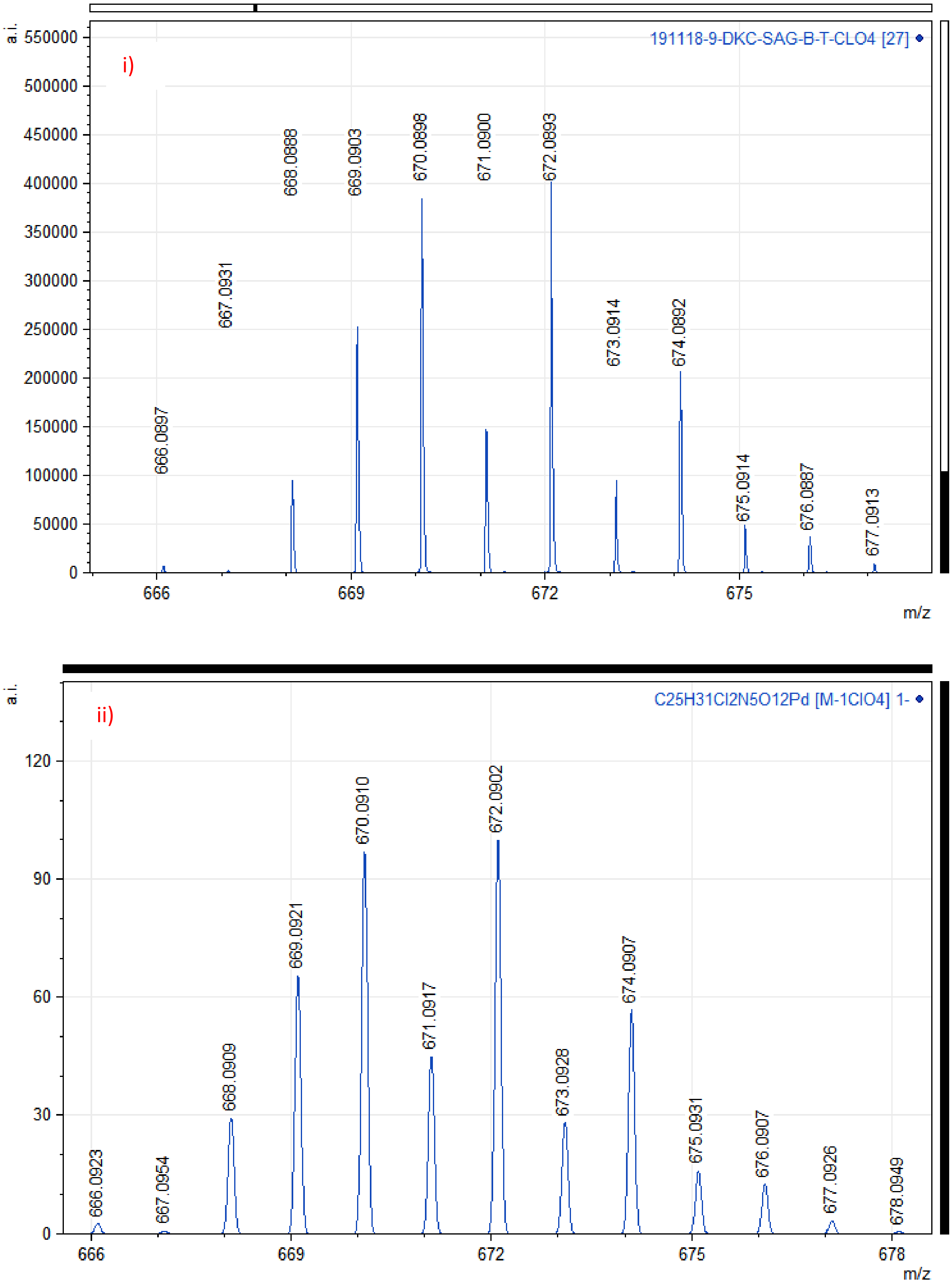

Figure S19a: ESIMS, isotopic pattern for [**1c** – ClO_4]$^+$ i) experimental and ii) theoretical.

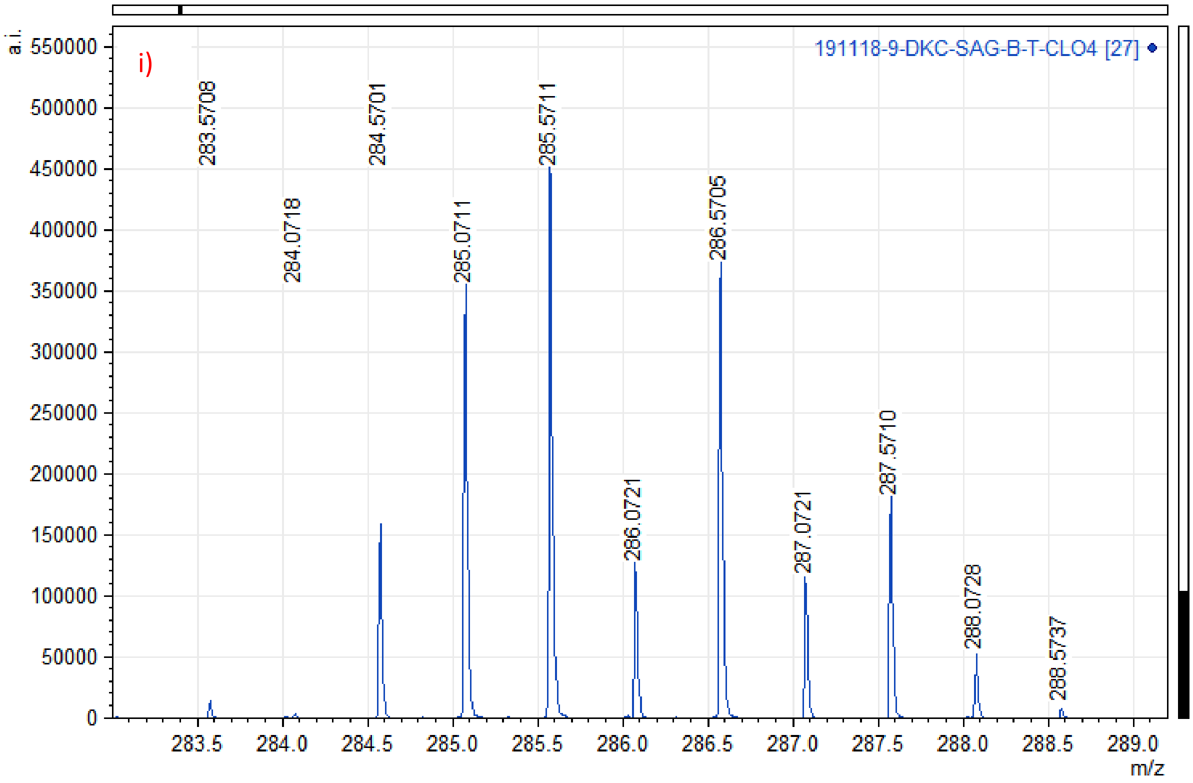

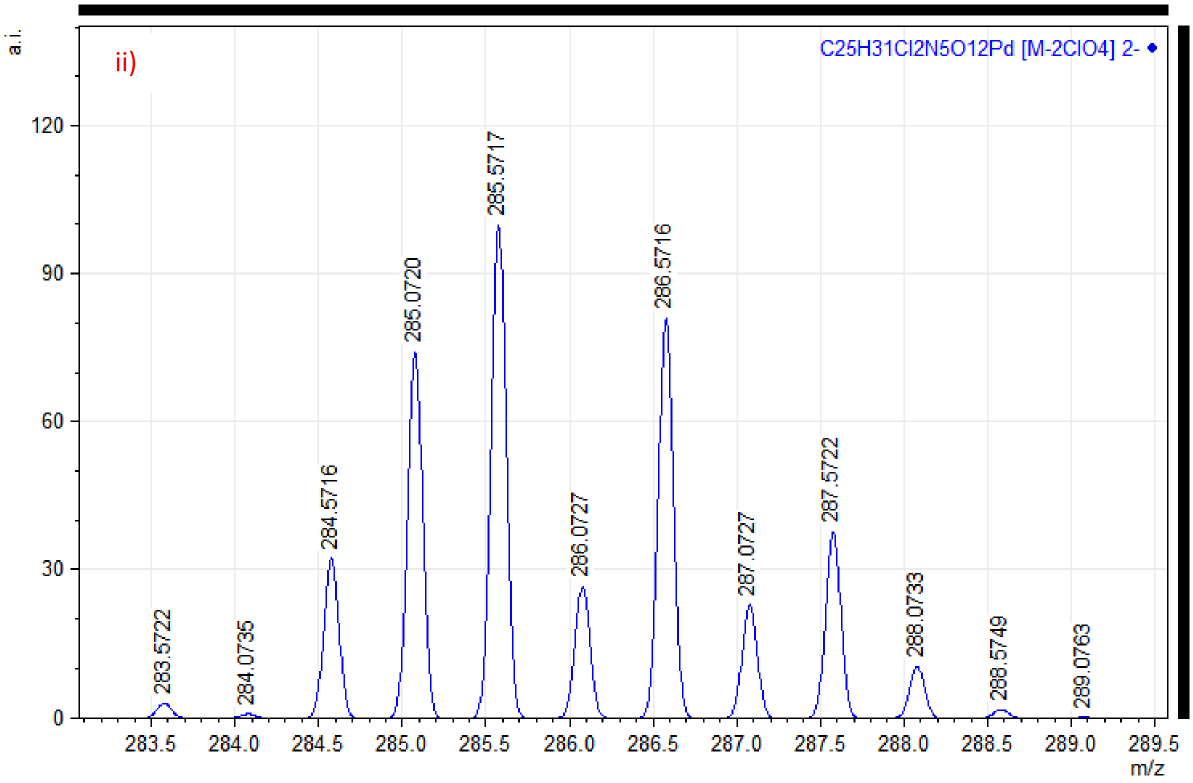

Figure S19b: ESIMS, isotopic pattern for [**1c** − $2ClO_4]^{2+}$ i) experimental and ii) theoretical.

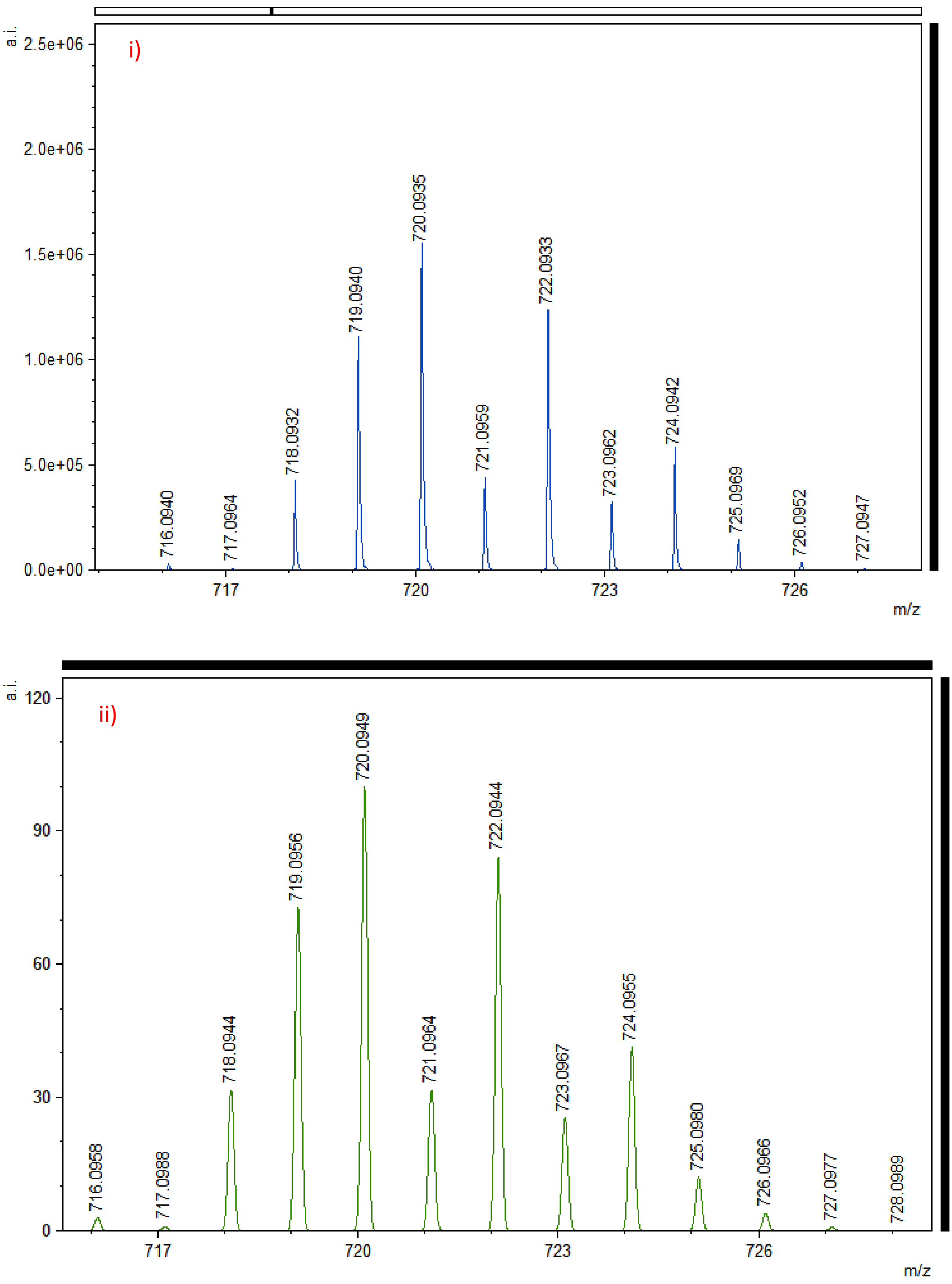

Figure S20a: ESIMS, isotopic pattern for [**1d** − OTf]$^{+}$ i) experimental and ii) theoretical.

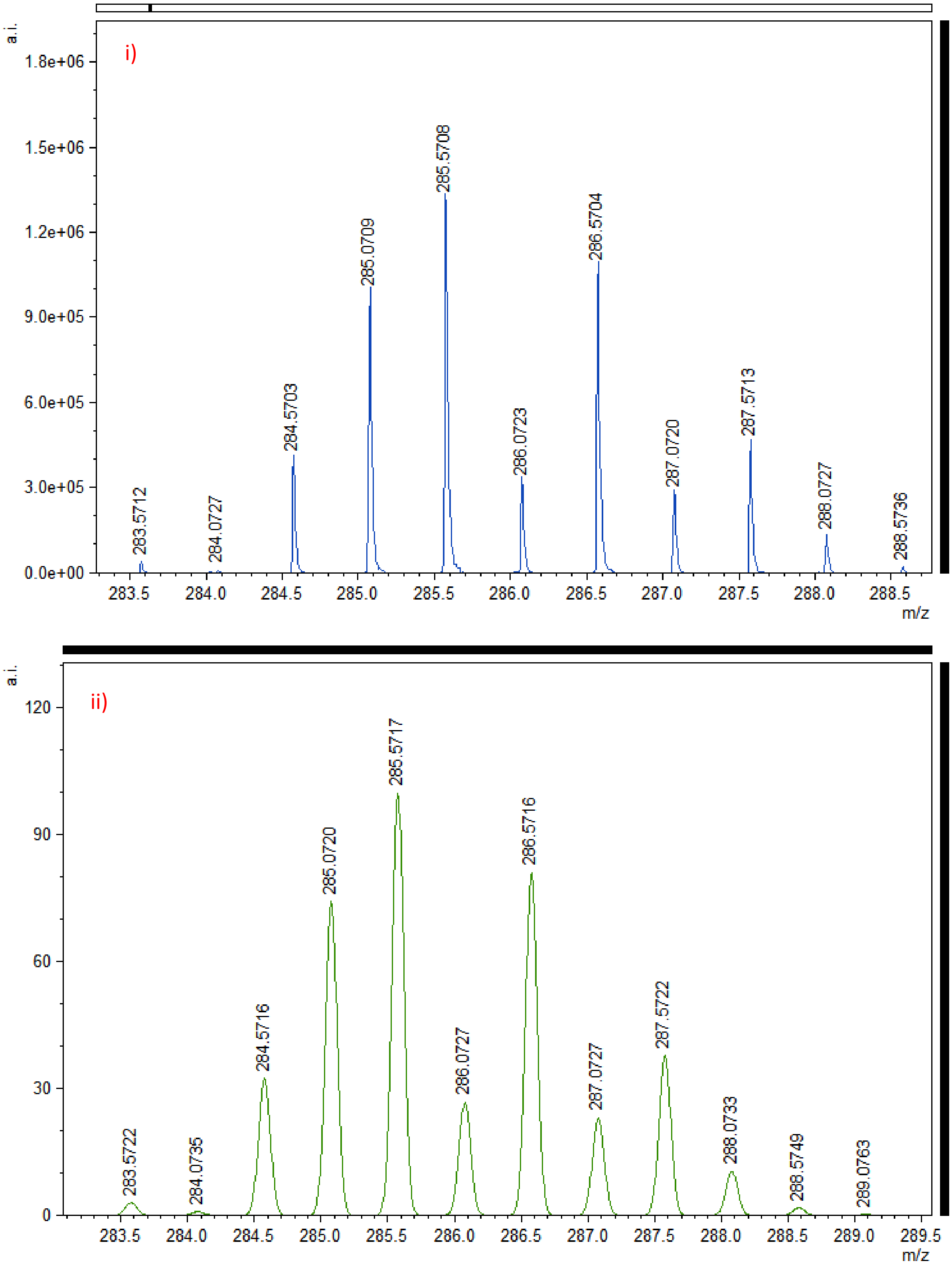

Figure S20b: ESIMS, isotopic pattern for [**1d** − 2OTf]$^{2+}$ i) experimental and ii) theoretical.

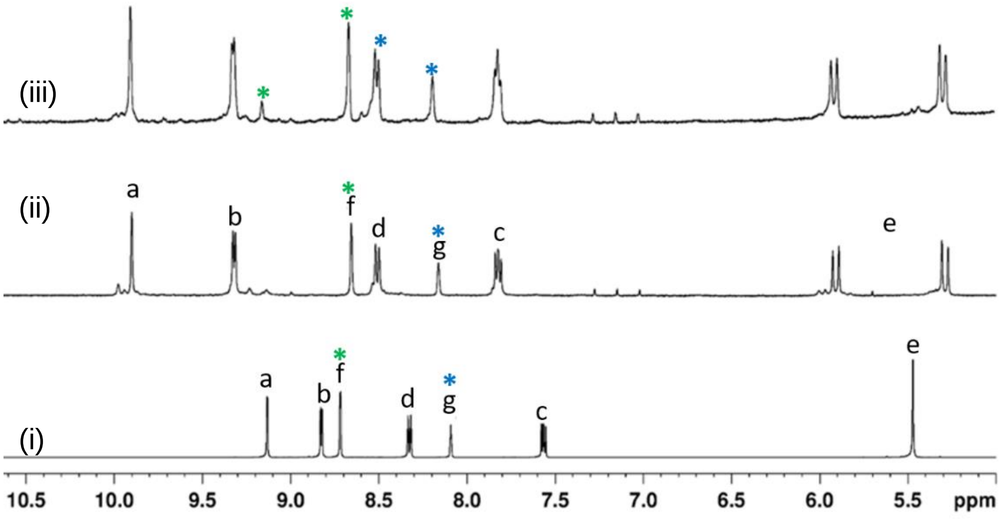

Figure S21: Partial ^{1}H NMR spectra (400 MHz, DMSO-d_6, 300 K) for (i) ligand **L1**; (ii), (iii) monitoring the reaction of **L1** and Pd(tmeda)$(NO_3)_2$ in 2:3 ratio (ii) after 24 h formation of [Pd(tmeda)(**L1**)]$(NO_3)_2$, **1a**; (iii) upon heating the mixture for further 72 h at 90 °C [Pd(tmeda)(**L1**)]$(NO_3)_2$,, **1a** and [Pd_3(tmeda)$_3$(**L1)**$_2$]$(NO_3)_6$, **2a** (20 percentage).

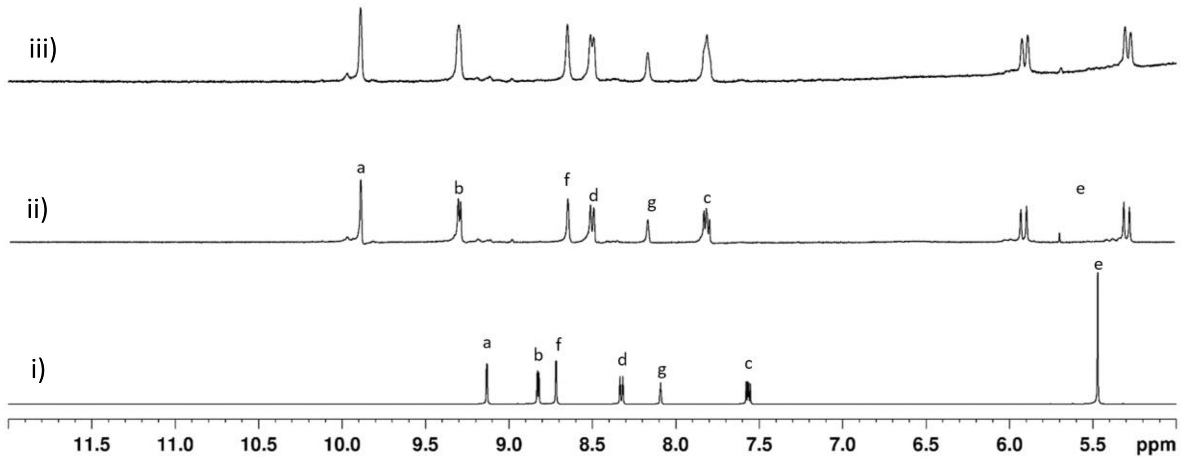

Figure S22: Partial ^{1}H NMR spectra (400 MHz, DMSO-d_6, 300 K) for (i) ligand **L1**; (ii), (iii) monitoring the reaction of **L1** and Pd(tmeda)$(BF_4)_2$ in 2:3 ratio (ii) after 24 h formation of [Pd(tmeda)(**L1**)]$(BF_4)_2$, **1b**; (iii) upon heating the mixture for further 72 h at 90 °C there was no change complex [Pd(tmeda)(**L1**)]$(BF_4)_2$, **1b** retained as such.

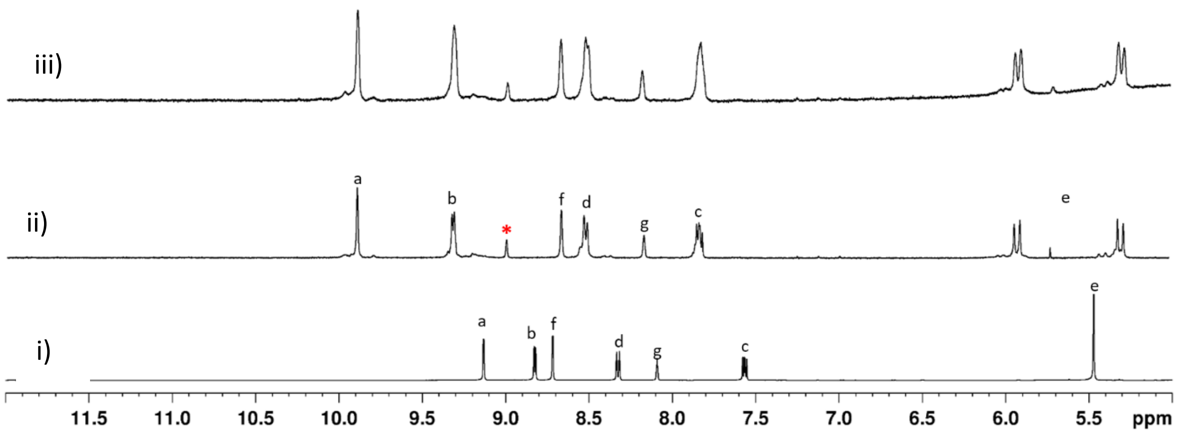

Figure S23: Partial ^{1}H NMR spectra (400 MHz, DMSO-d_6, 300 K) for (i) ligand **L1**; (ii), (iii) monitoring the reaction of **L1** and Pd(tmeda)(ClO_4)$_2$ in 2:3 ratio (ii) after 24 h formation of [Pd(tmeda)(**L1**)](ClO_4)$_2$, **1c** and [Pd_3(tmeda)$_3$(**L1)**$_2$](ClO_4)$_6$, **2c** (20 percentage) (iii) upon heating the mixture for further 72 h at 90 °C there was no change in the percentage of formation of [Pd_3(tmeda)$_3$(**L1)**$_2$](ClO_4)$_6$, **2c**.

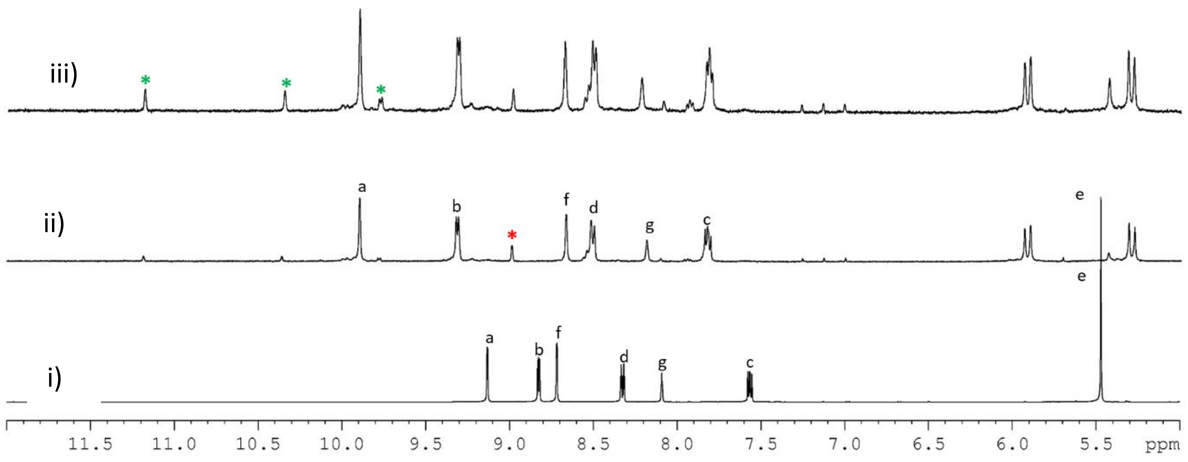

Figure S24: Partial ^{1}H NMR spectra (400 MHz, DMSO-d_6, 300 K) for (i) ligand **L1**; (ii), (iii) monitoring the reaction of **L1** and Pd(tmeda)(**L1**)(OTf)$_2$ in 2:3 ratio (ii) after 24 h formation of [Pd(tmeda)(**L1**)](OTf)$_2$, **1d** and [Pd_3(tmeda)$_3$(**L1)**$_2$](OTf)$_6$, **2d** (20 percentage) (iii) upon heating the mixture for further 72 h at 90 °C there was no change in the percentage of formation of [Pd_3(tmeda)$_3$(**L1)**$_2$](OTf)$_6$, **2d**. (Some amount of [(Cl)$_2\subset$$Pd_3$(**L1**)$_4$](OTf)$_4$, **6d** was detected unexpectedly, probably due to chloride impurity.)

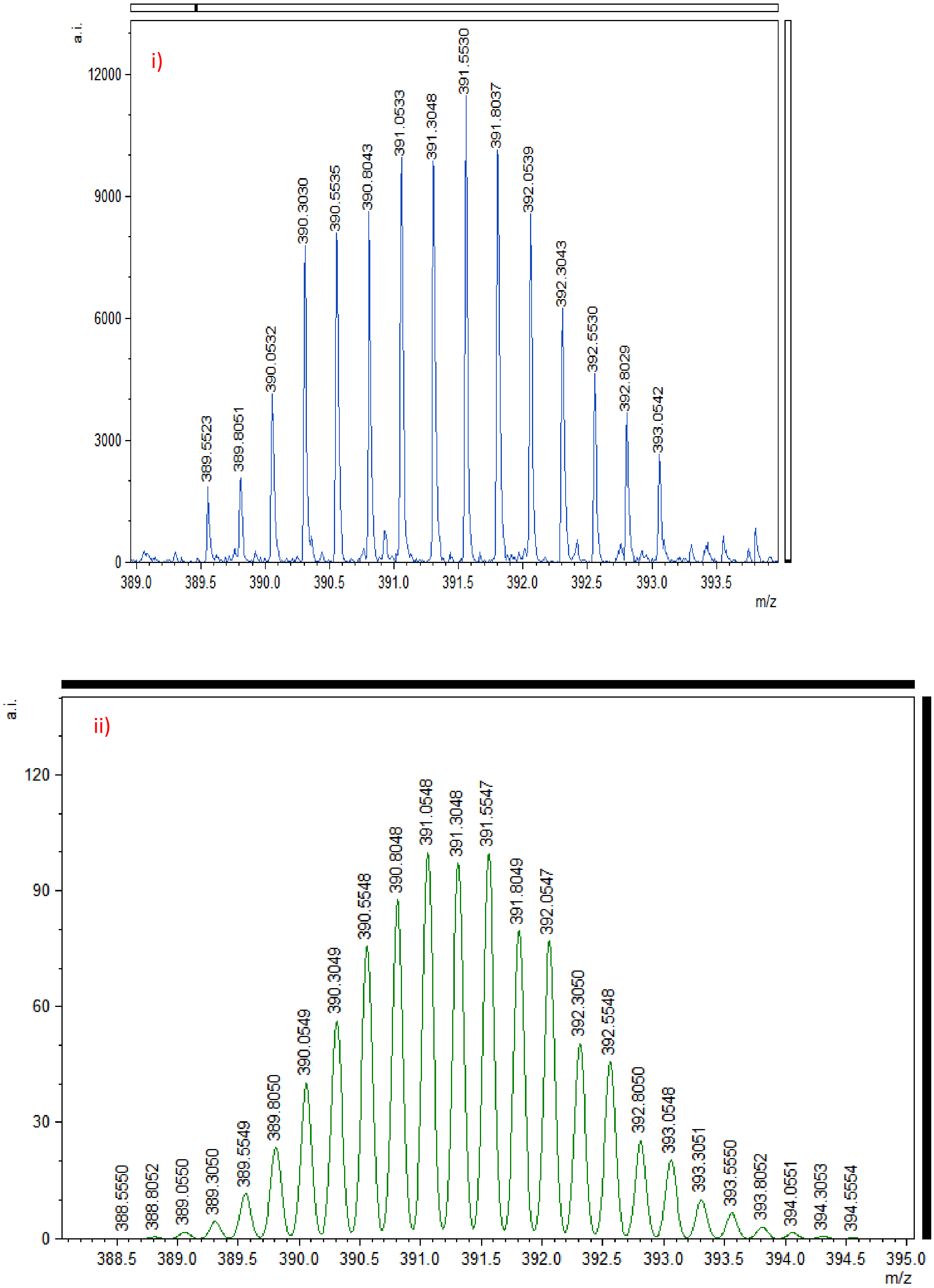

Figure S25: ESIMS, isotopic pattern for [**2c** – ClO_4]$^{4+}$ i) experimental and ii) theoretical.

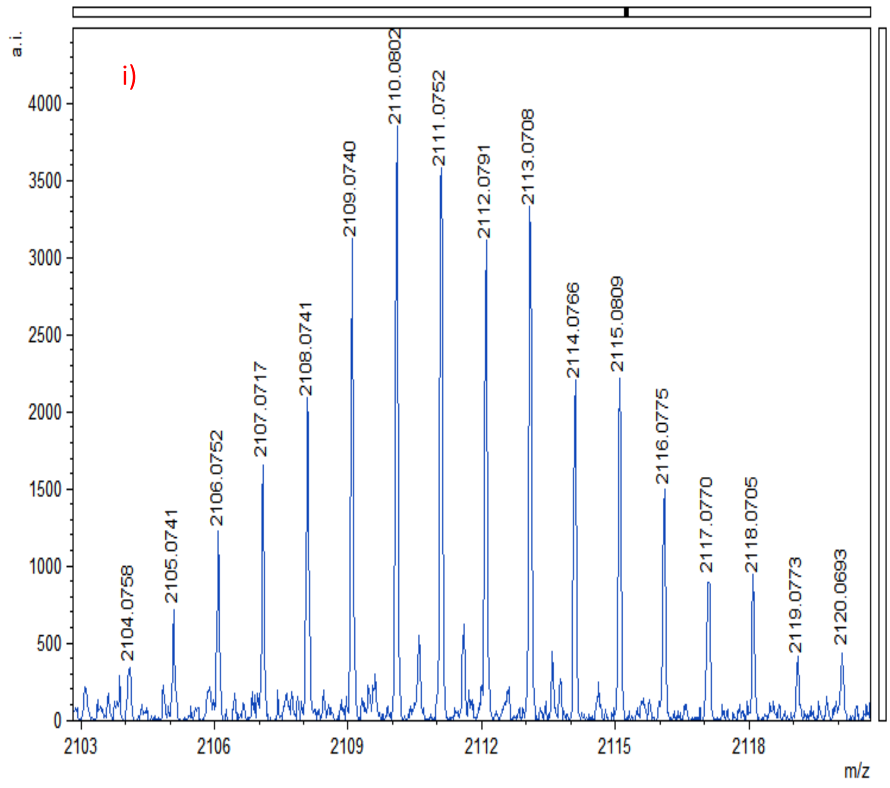

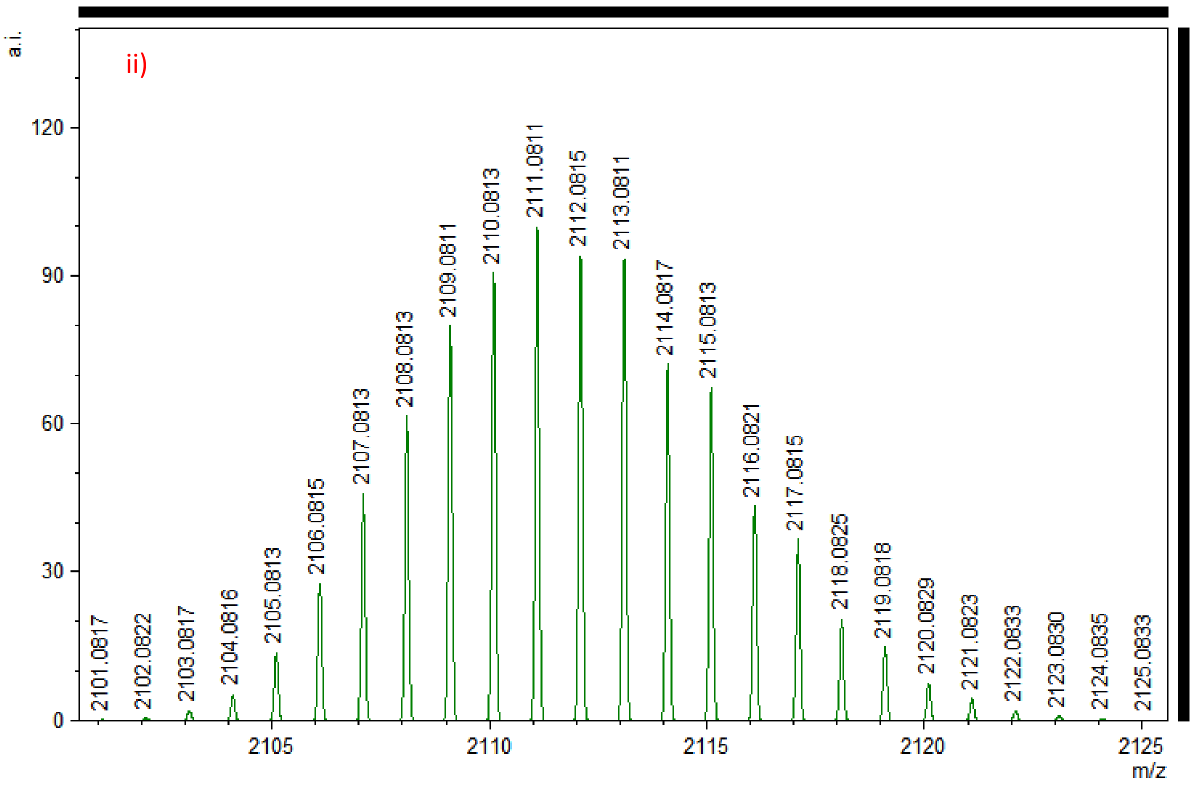

Figure S26: ESI-MS, isotopic pattern for [**2d** − OTf]$^{+1}$ i) experimental and ii) theoretical.

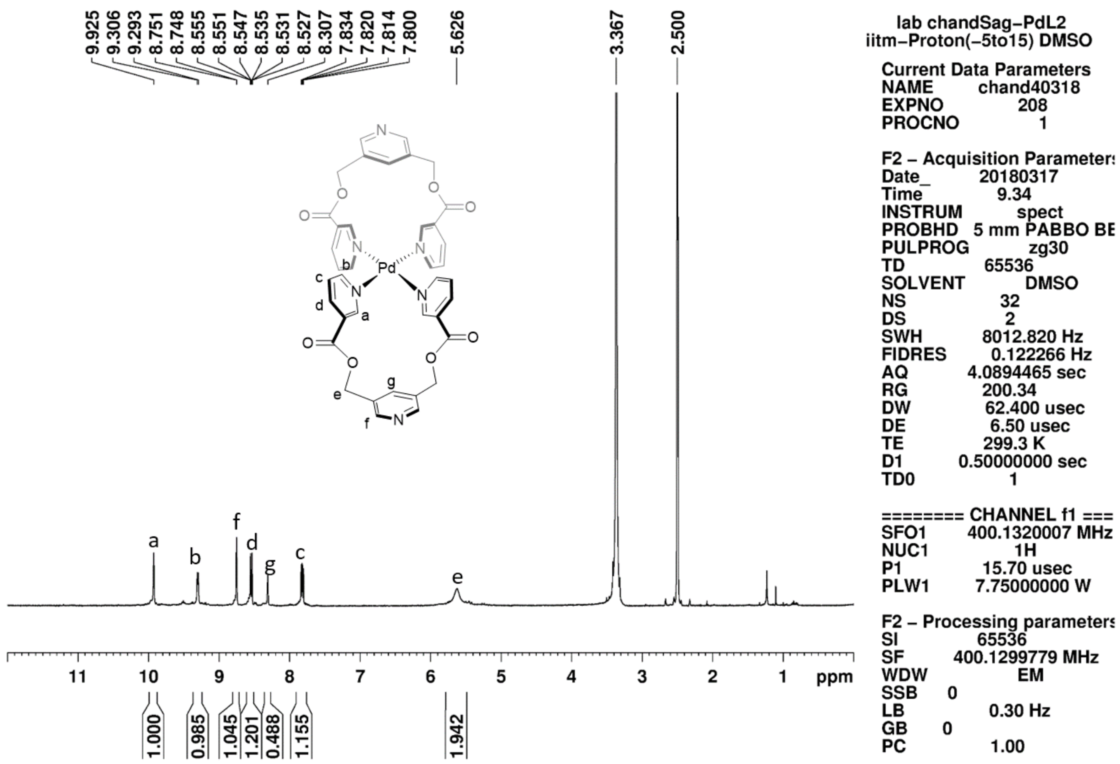

Figure S27: ^{1}H NMR (400 MHz, DMSO-d_6, 300 K) for [Pd(**L1**)$_2$](NO$_3$)$_2$, **3a**.

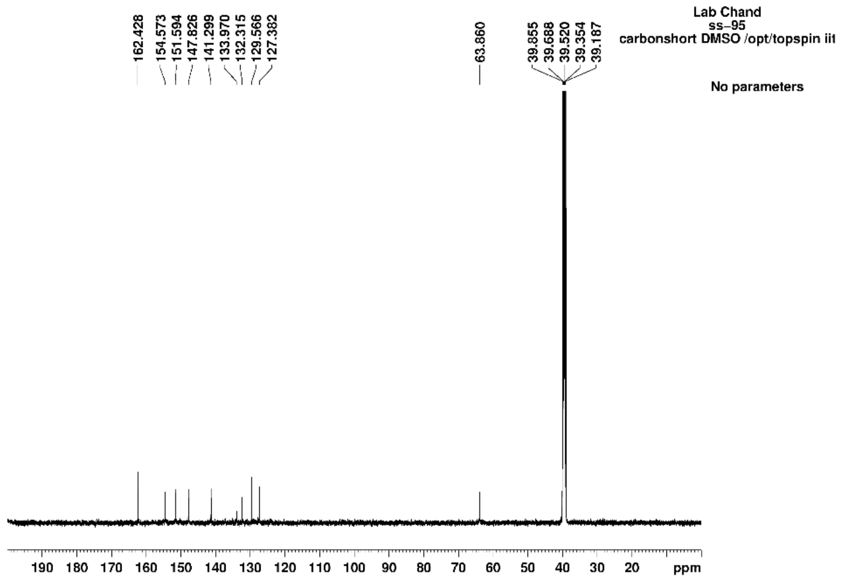

Figure S28: ^{13}C NMR (125 MHz, DMSO-d_6, 300 K) for [Pd(**L1**)$_2$](NO$_3$)$_2$, **3a**.

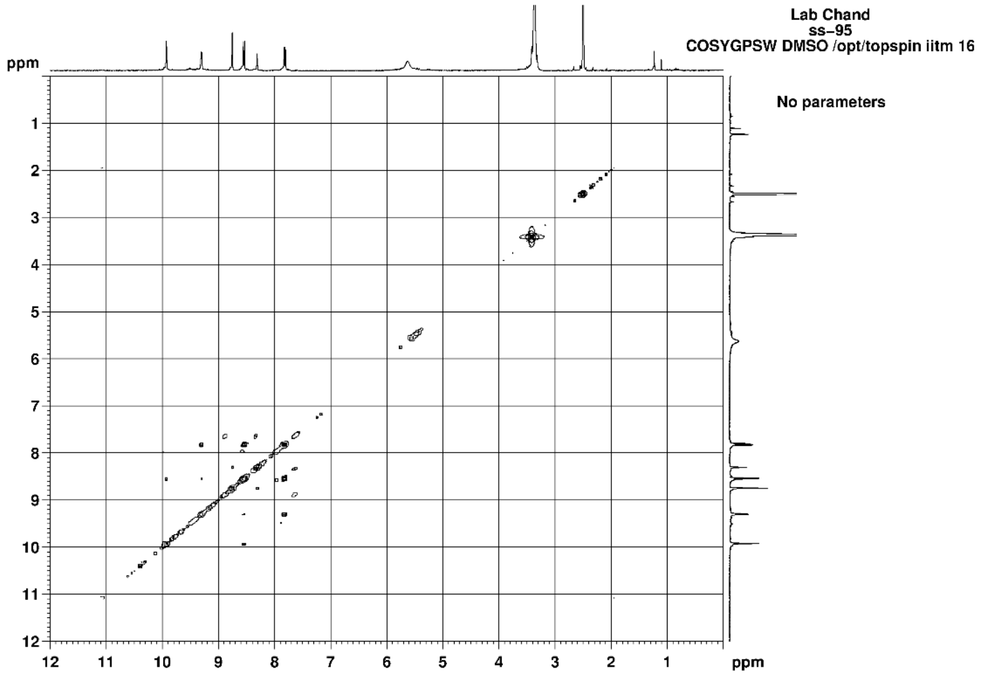

Figure S29a: H-H COSY (500 MHz, DMSO-d_6, 300 K) for [Pd(**L1**)$_2$](NO$_3$)$_2$, **3a**.

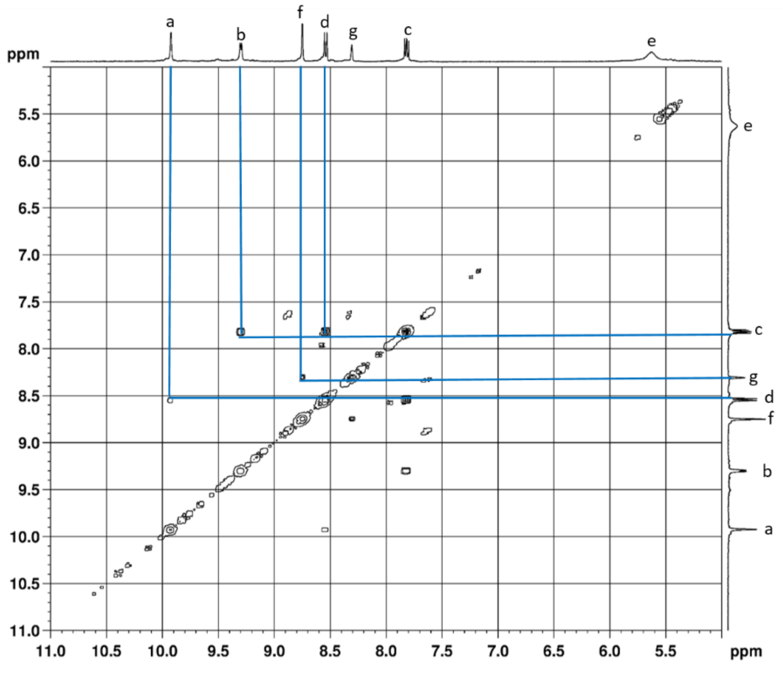

Figure S29b: Expansion of H-H COSY (500 MHz, DMSO-d_6, 300 K) for [Pd(**L1**)$_2$](NO$_3$)$_2$, **3a**.

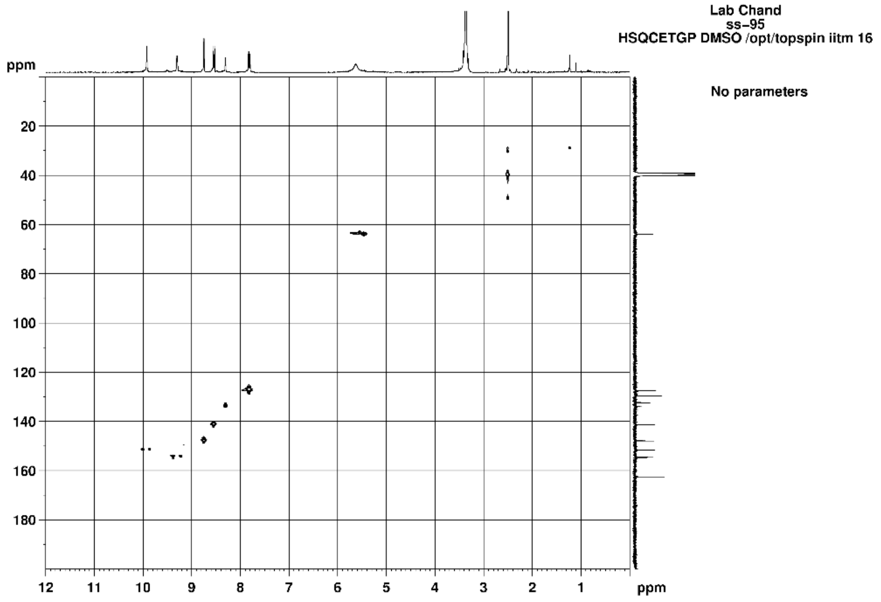

Figure S30a: C-H COSY (500 MHz, DMSO-d_6, 300 K) for $[Pd(\mathbf{L1})_2](NO_3)_2$, **3a**.

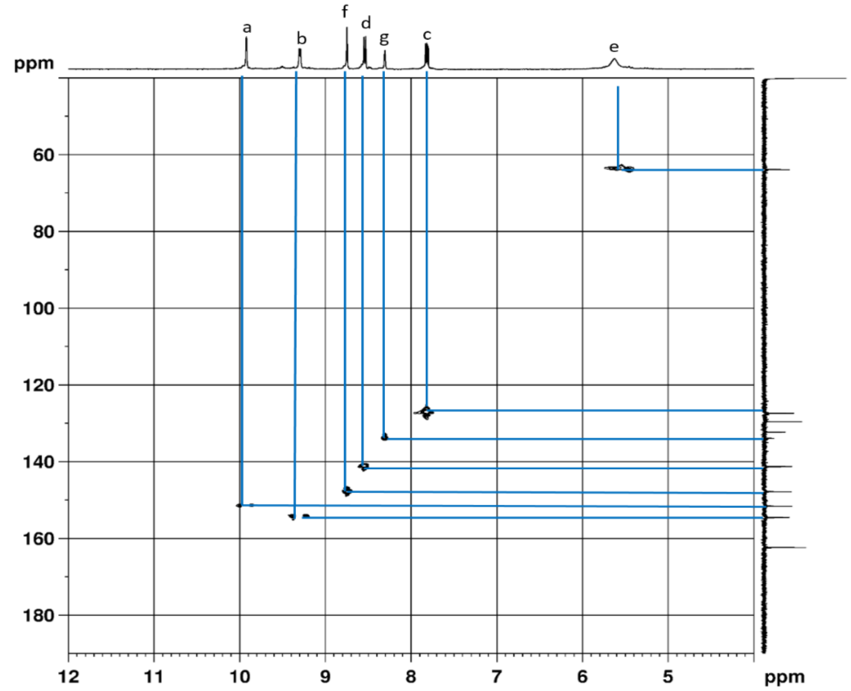

Figure S30b: Expansion of C-H COSY (500 MHz, DMSO-d_6, 300 K) for $[Pd(\mathbf{L1})_2](NO_3)_2$, **3a**.

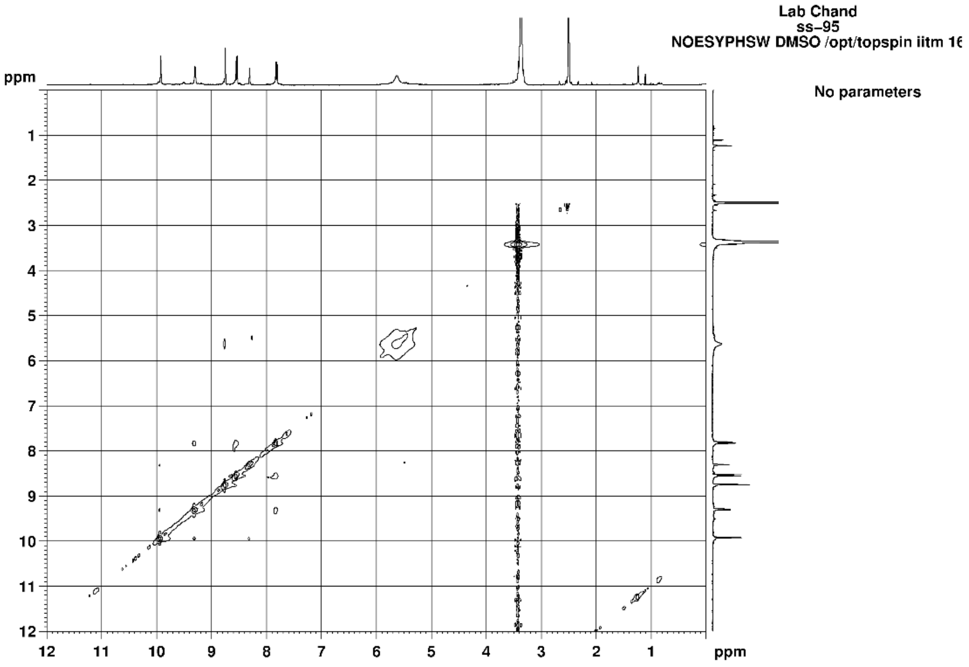

Figure S31a: NOESY (500 MHz, DMSO-d_6, 300 K) for [Pd(**L1**)$_2$](NO$_3$)$_2$, **3a**.

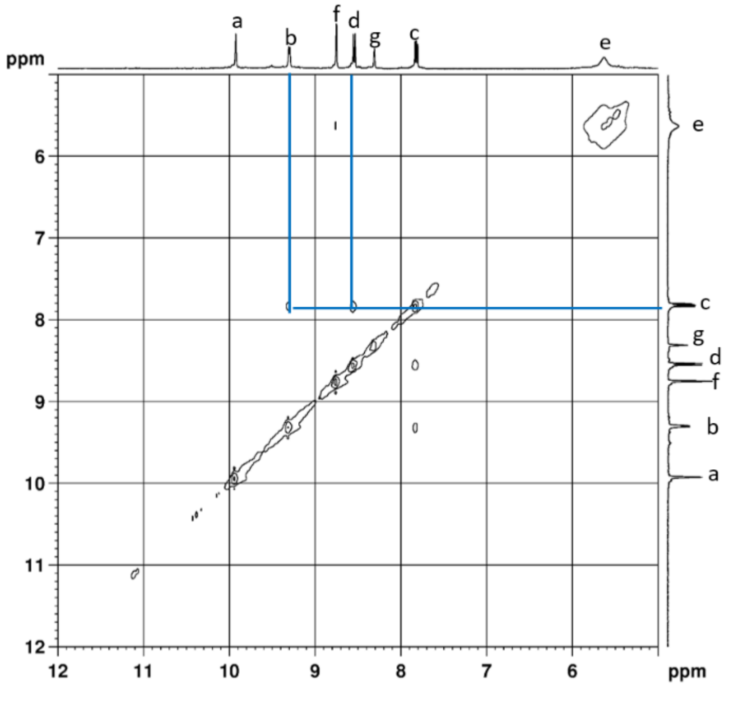

Figure S31b: Expansion for NOESY (500 MHz, DMSO-d_6, 300 K) for [Pd(**L1**)$_2$](NO$_3$)$_2$, **3a**.

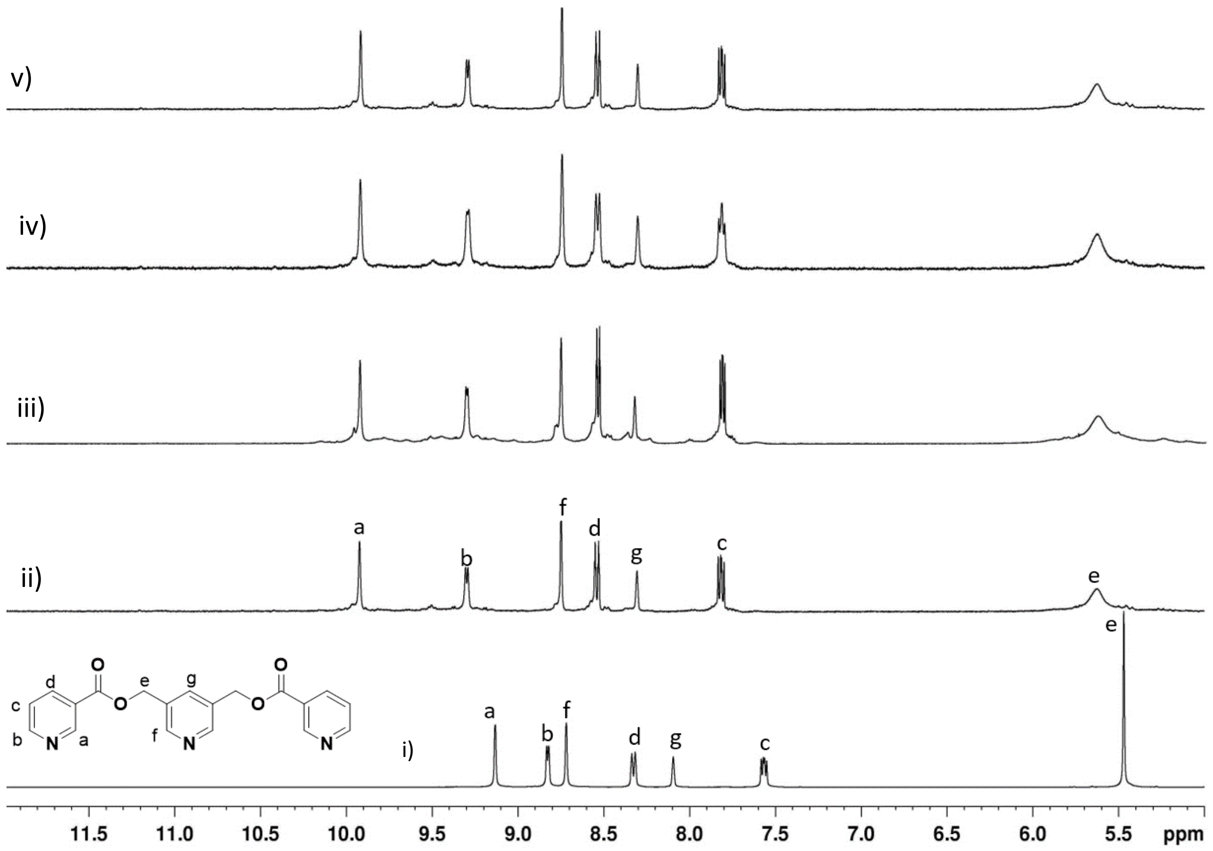

Figure S32: Partial ^{1}H NMR spectra (400 MHz, DMSO-d_6, 300 K) for (i) ligand **L1**; (ii) [Pd(**L1**)$_2$](NO_3)$_2$, **3a**; (iii) [Pd(**L1**)$_2$](BF_4)$_2$, **3b**; (iv) [Pd(**L1**)$_2$](ClO_4)$_2$, **3c** and (v) [Pd(**L1**)$_2$](OTf)$_2$, **3d**.

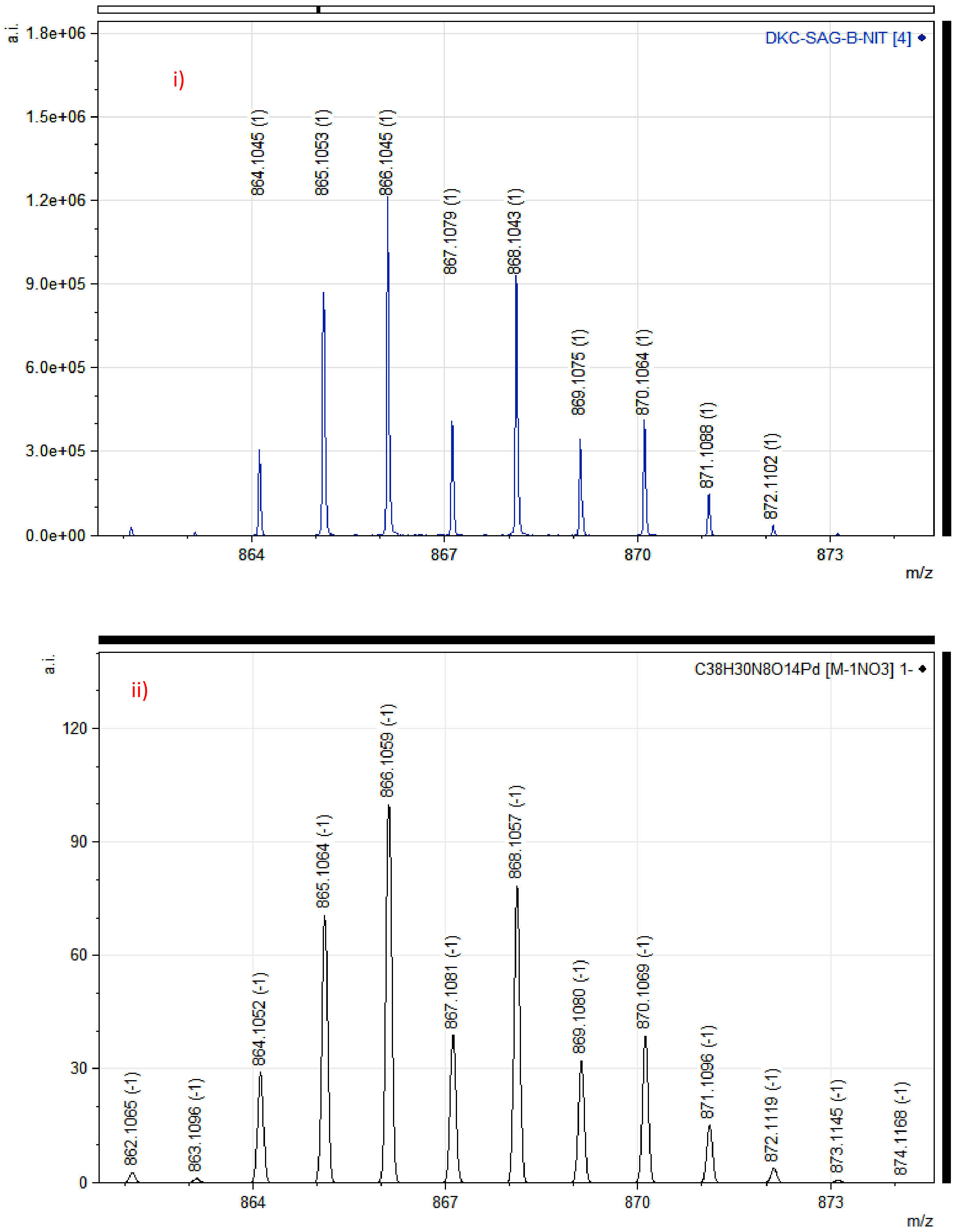

Figure S33a: ESIMS, isotopic pattern for [**3a** − NO_3]$^+$ i) experimental and ii) theoretical.

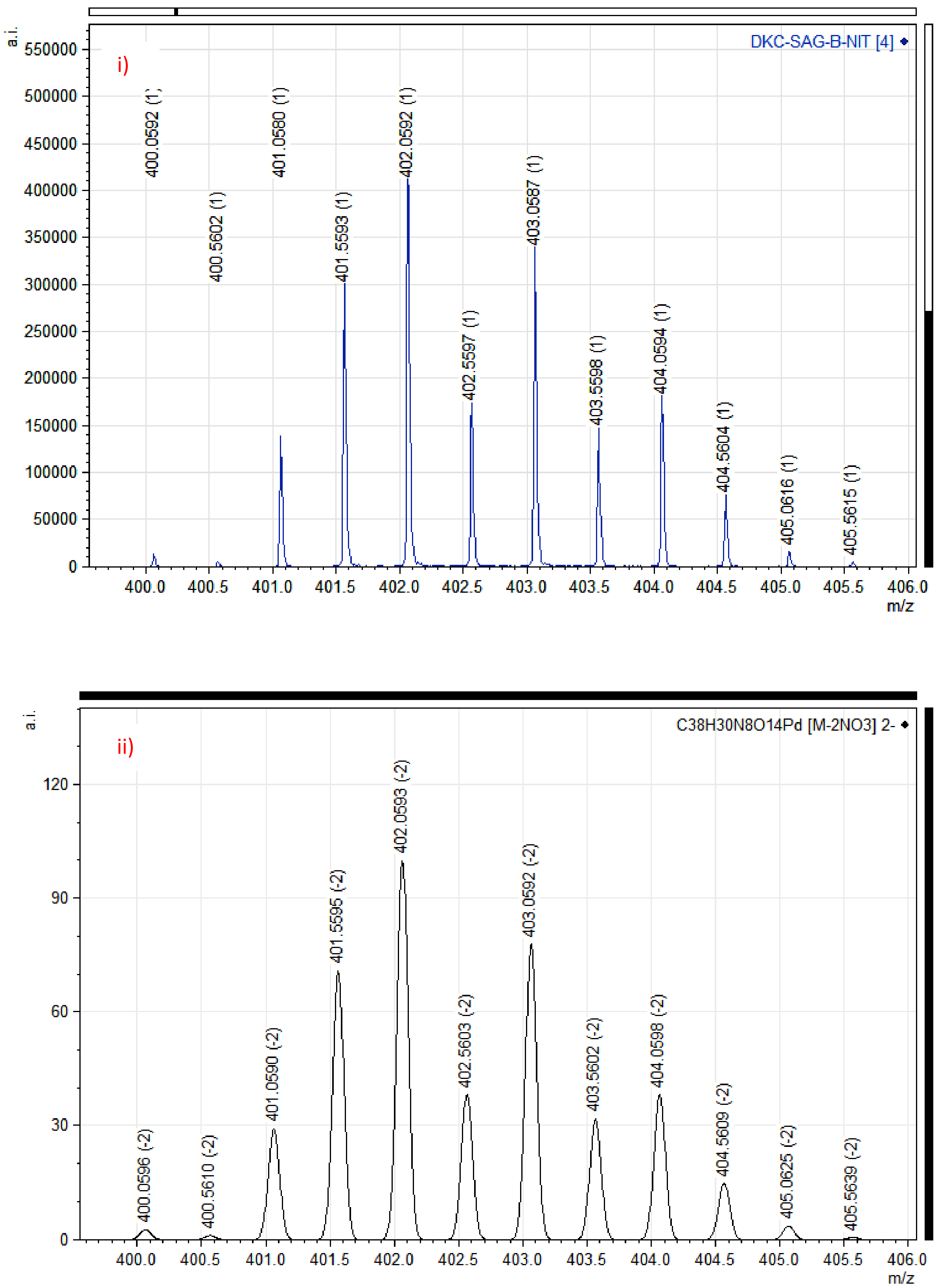

Figure S33b: ESIMS, isotopic pattern for [**3a** − $2NO_3]^{2+}$ i) experimental and ii) theoretical.

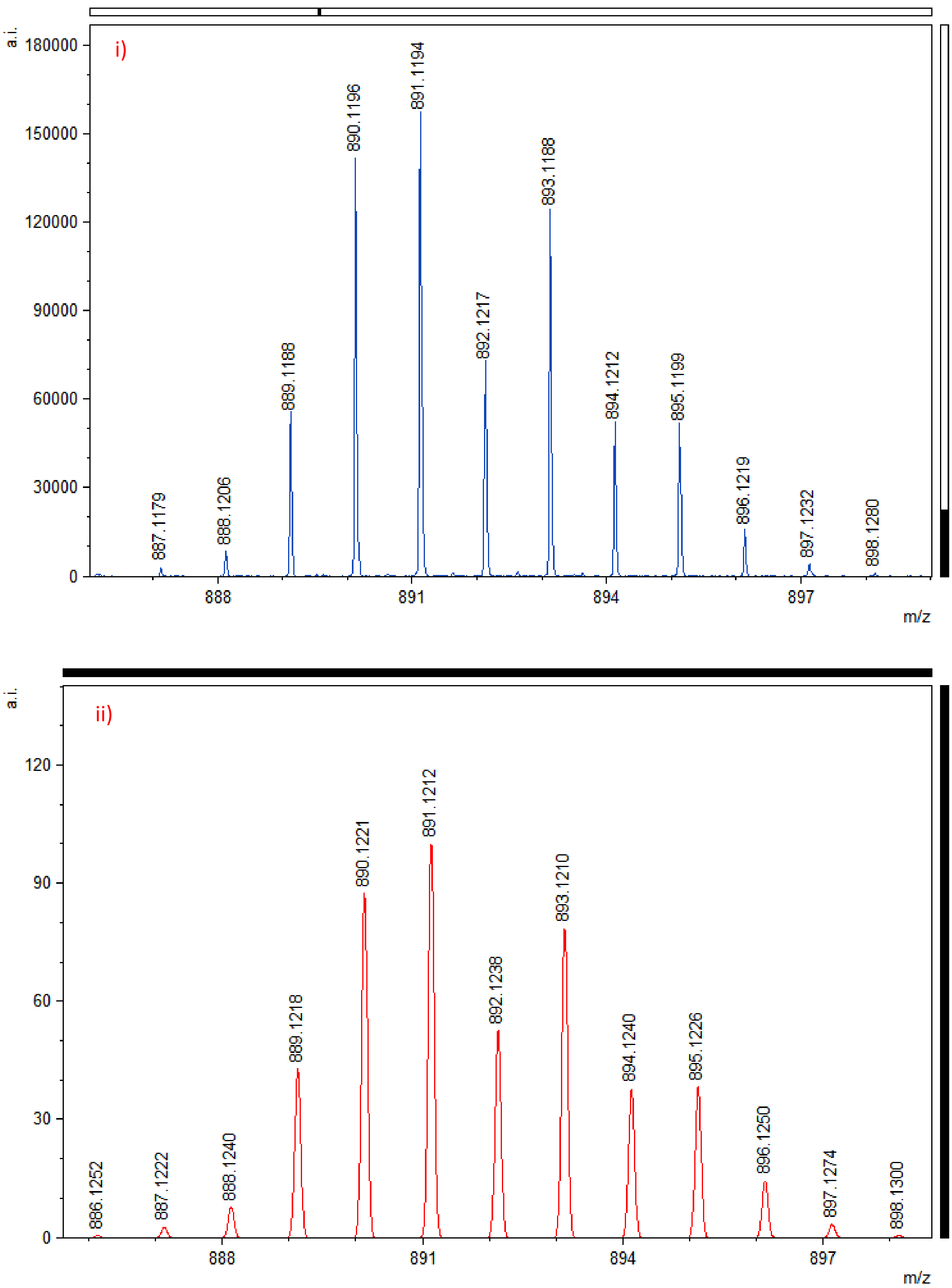

Figure S34a: ESIMS, isotopic pattern for [**3b** − BF_4]$^+$ i) experimental and ii) theoretical.

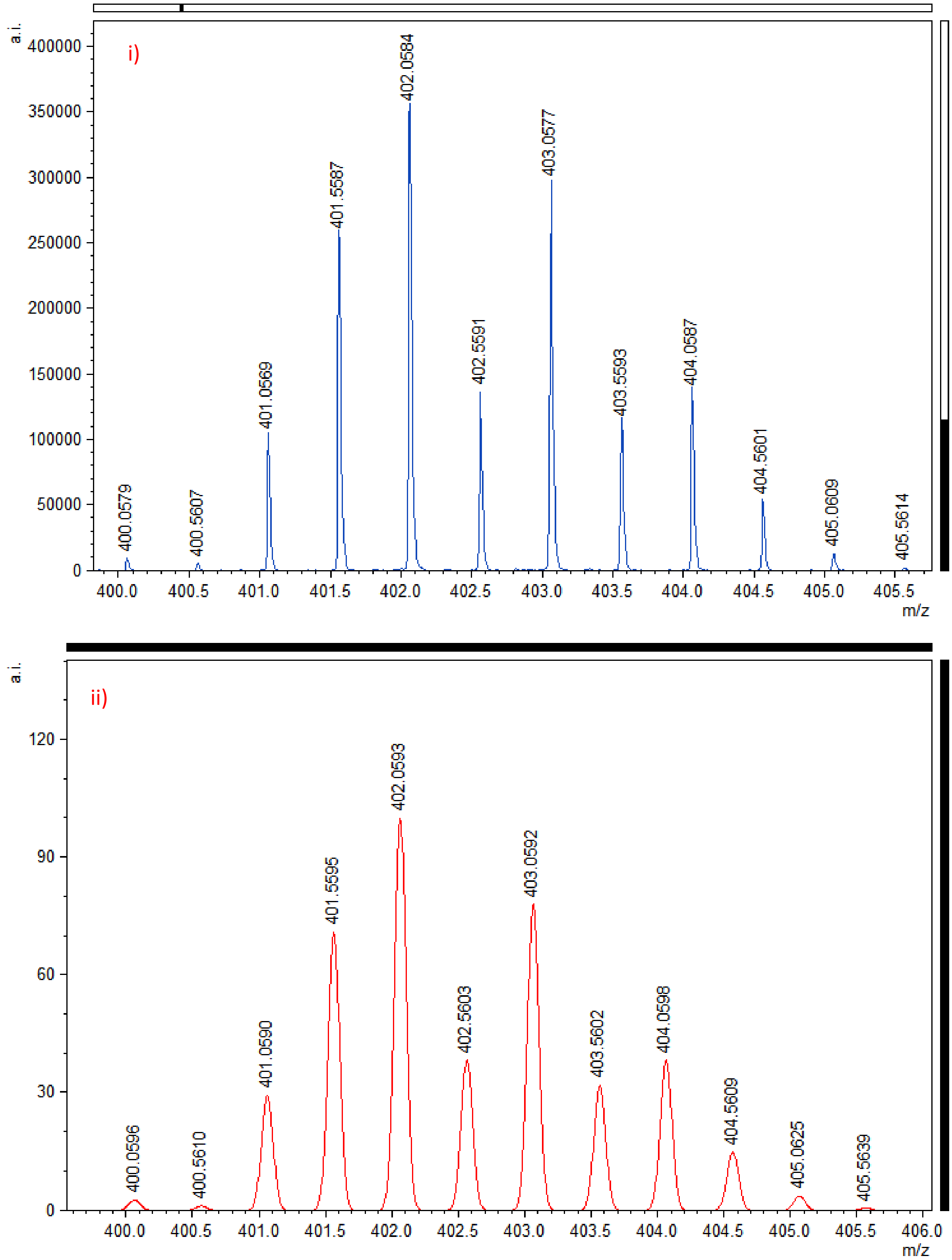

Figure S34b: ESIMS, isotopic pattern for [**3b** − $2BF_4$]$^{2+}$ i) experimental and ii) theoretical.

Monitoring the formation of complex [$(NO_3)_2$@Pd_3(L1)$_4$]$(NO_3)_4$, 4a at room temperature:

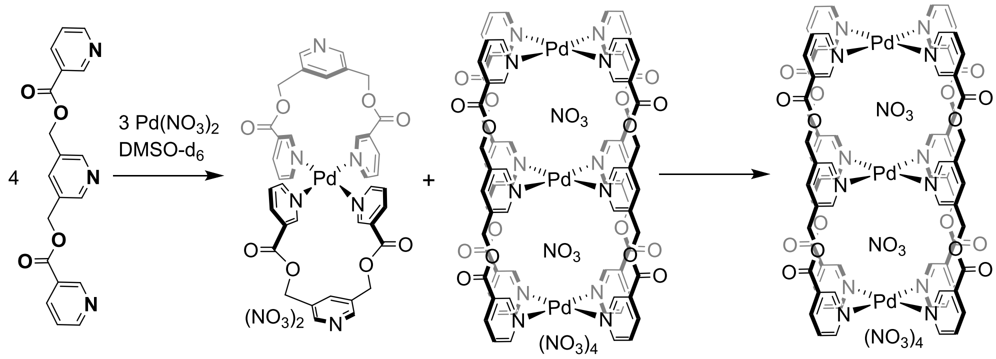

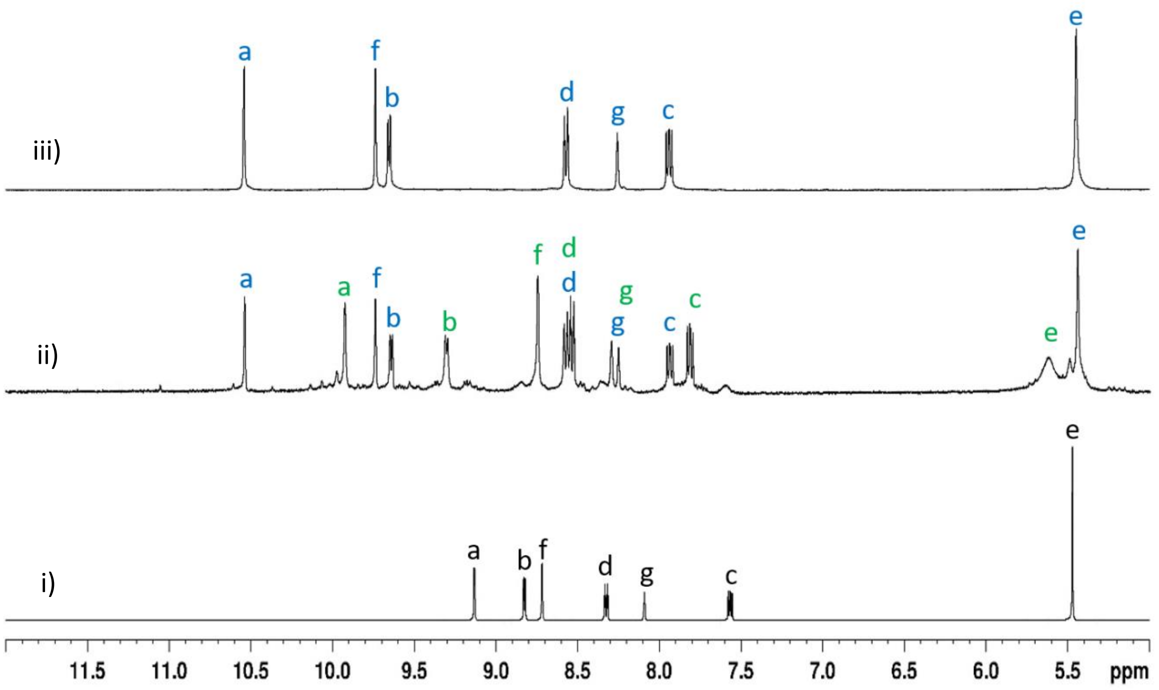

Figure S35a: Partial ^{1}H NMR spectra (400 MHz, DMSO-d_6, 300 K) for (i) ligand **L1**; (ii), (iii) monitoring the reaction of **L1** and $Pd(NO_3)_2$ in 4 :3 ratio upon mixing at room temperature (ii) formation of mixture of [Pd_1(**L1**)$_2$]$(NO_3)_2$, **3a** (labelled in green) and [$(NO_3)_2$@Pd_3(**L1**)$_4$]$(NO_3)_4$, **4a** (labelled in blue) after 10 min; (iii) exclusive formation of [$(NO_3)_2$@Pd_3(**L1**)$_4$]$(NO_3)_4$, **4a** after 20 min.

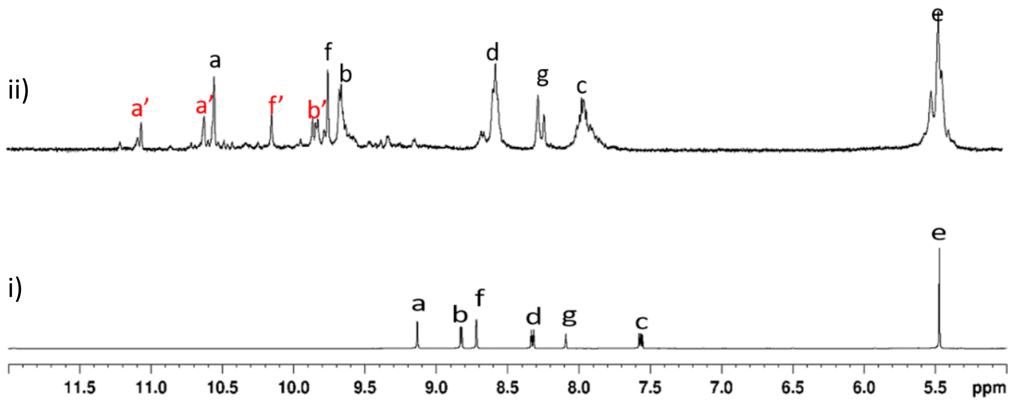

Figure S35b: Partial ^{1}H NMR spectra (400 MHz, DMSO-d_6, 300 K) for (i) ligand **L1**; (ii) the presence of $[(NO_3)_2@Pd_3(\mathbf{L1})_4](NO_3)_4$, **4a** peaks labelled in black color and $[(NO_3)(Cl)@Pd_3(\mathbf{L1})_4](NO_3)_4$, **5a'** peaks labelled in red color. [When the metal-to-ligand ratio was 3:4 and the $Pd(NO_3)_2$ was prepared from $PdCl_2$]

Monitoring the formation of complex $[(NO_3)_2@Pd_3(L1)_4](NO_3)_4$, 4a at 90 °C:

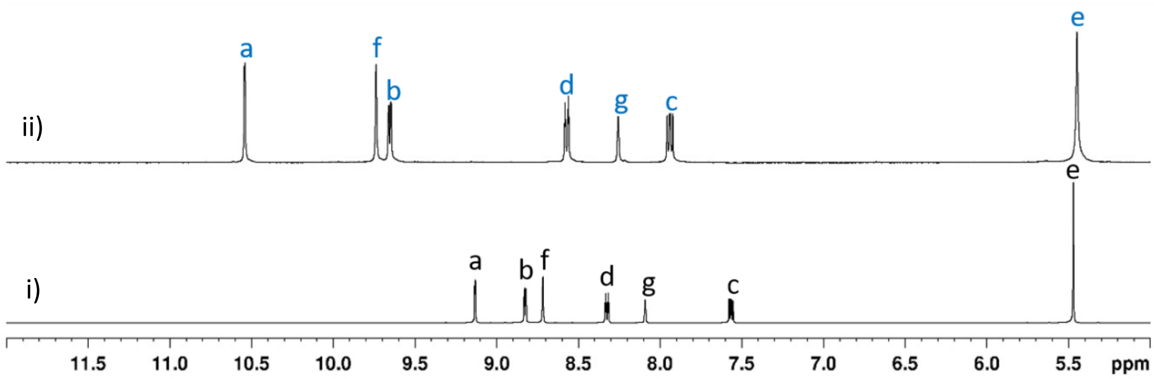

Figure S36: Partial ^{1}H NMR spectra (400 MHz, DMSO-d_6, 300 K) for (i) ligand **L1**; (ii) exclusive formation of $[(NO_3)_2@Pd_3(\mathbf{L1})_4](NO_3)_4$, **4a** formed by stirring **L1** and $Pd(NO_3)_2$ in 4:3 ratio for 5 min at 90 °C.

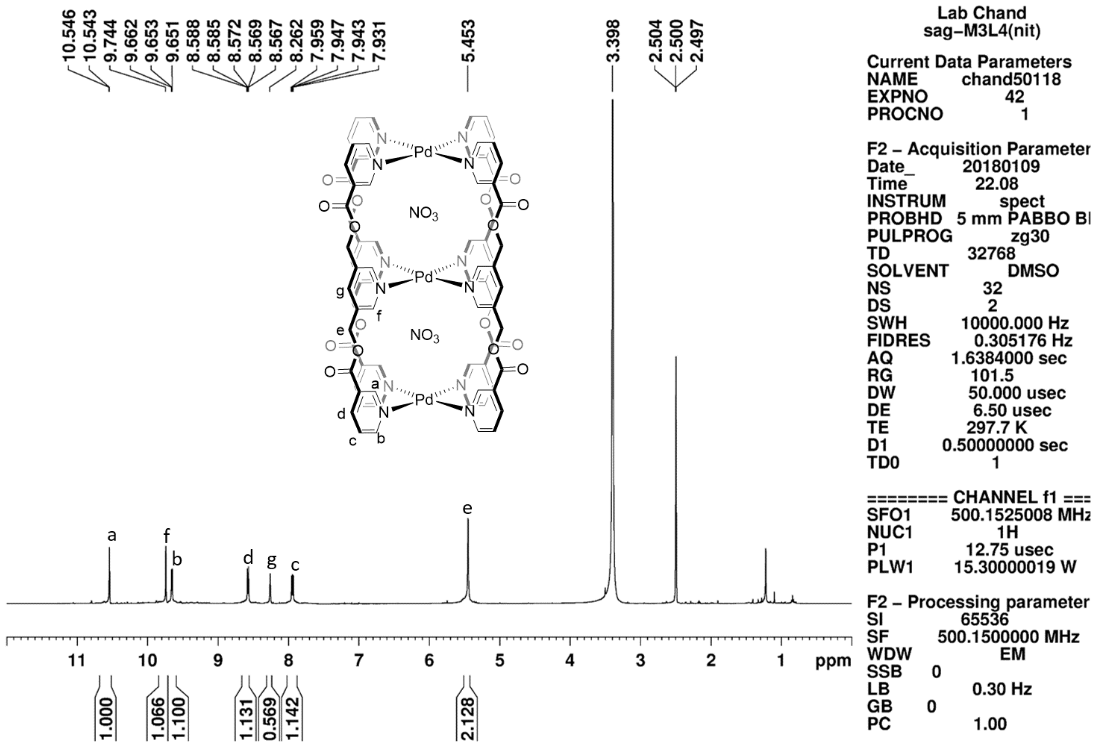

Figure S37: ^{1}H NMR (500 MHz, DMSO-d_6, 300 K) for $[(NO_3)_2@Pd_3(\mathbf{L1})_4](NO_3)_4$, **4a**.

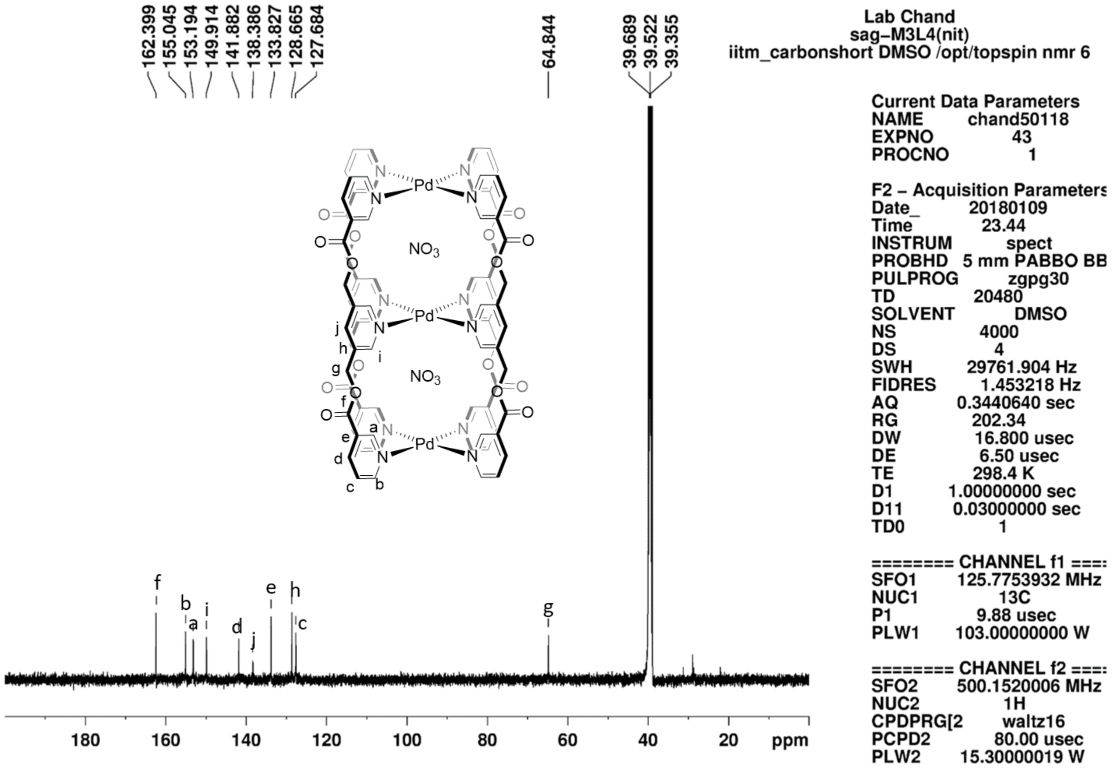

Figure S38: ^{13}C NMR (125 MHz, DMSO-d_6, 300 K) for $[(NO_3)_2@Pd_3(\mathbf{L1})_4](NO_3)_4$, **4a**.

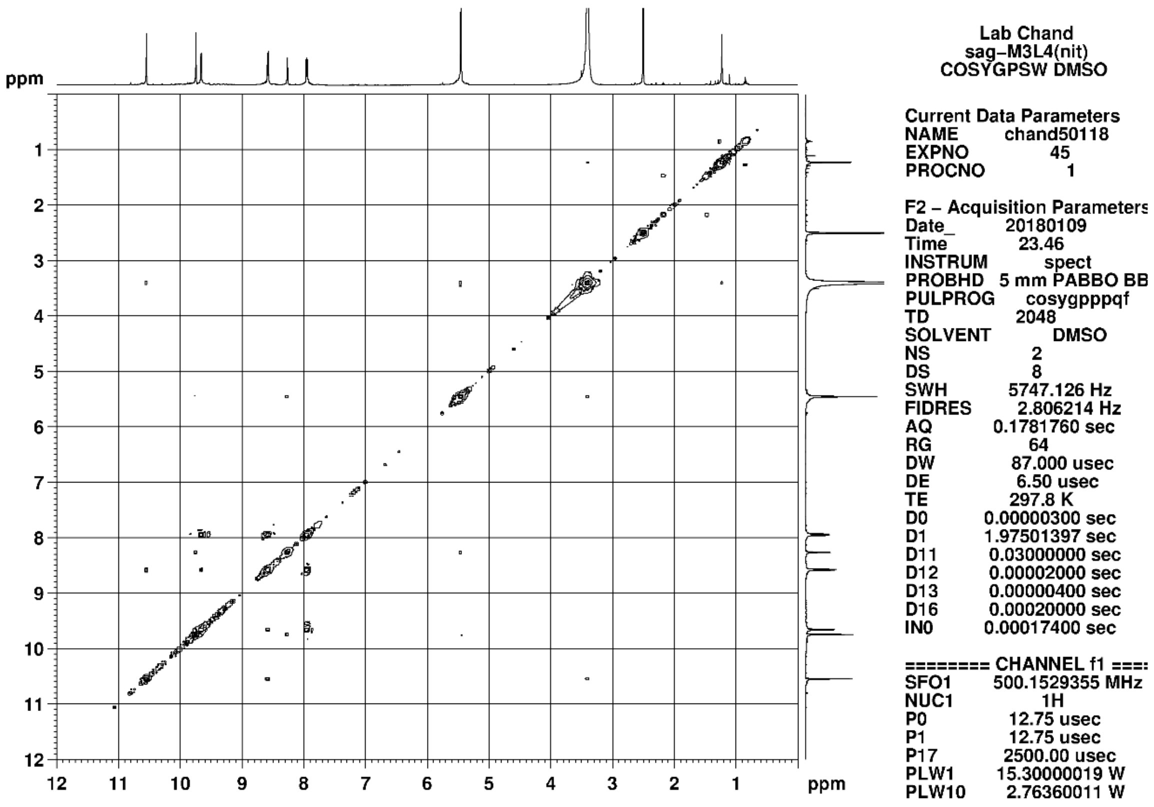

Figure S39a: H-H COSY (500 MHz, DMSO-d_6, 300 K) for $[(NO_3)_2@Pd_3(\mathbf{L1})_4](NO_3)_4$, **4a**.

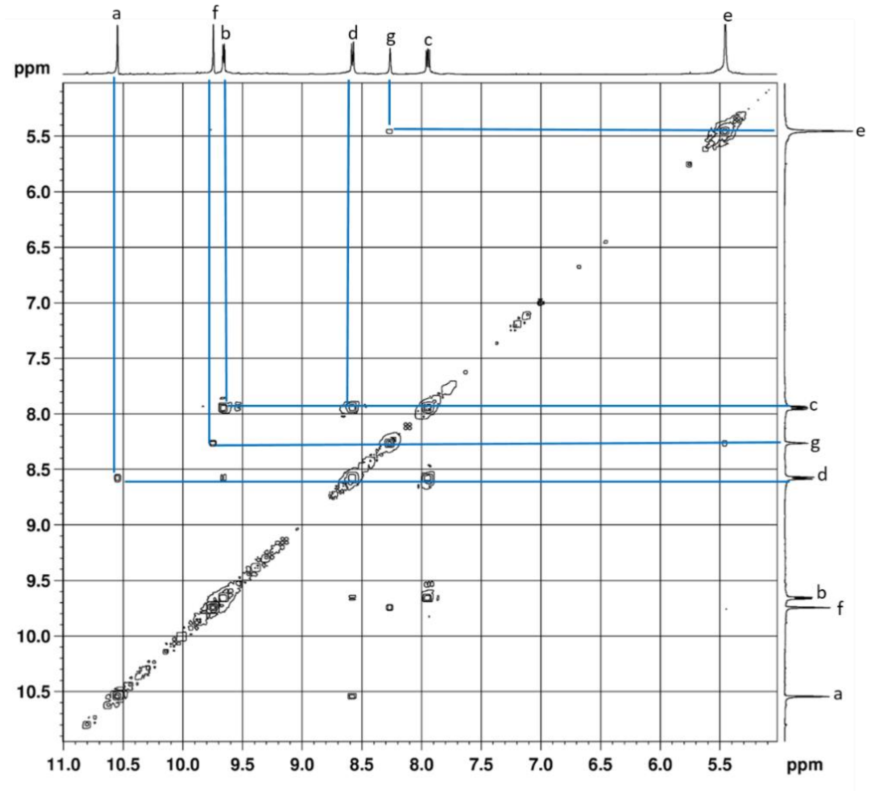

Figure S39b: H-H COSY expansion (500 MHz, DMSO-d_6, 300 K) for $[(NO_3)_2@Pd_3(\mathbf{L1})_4](NO_3)_4$, **4a**.

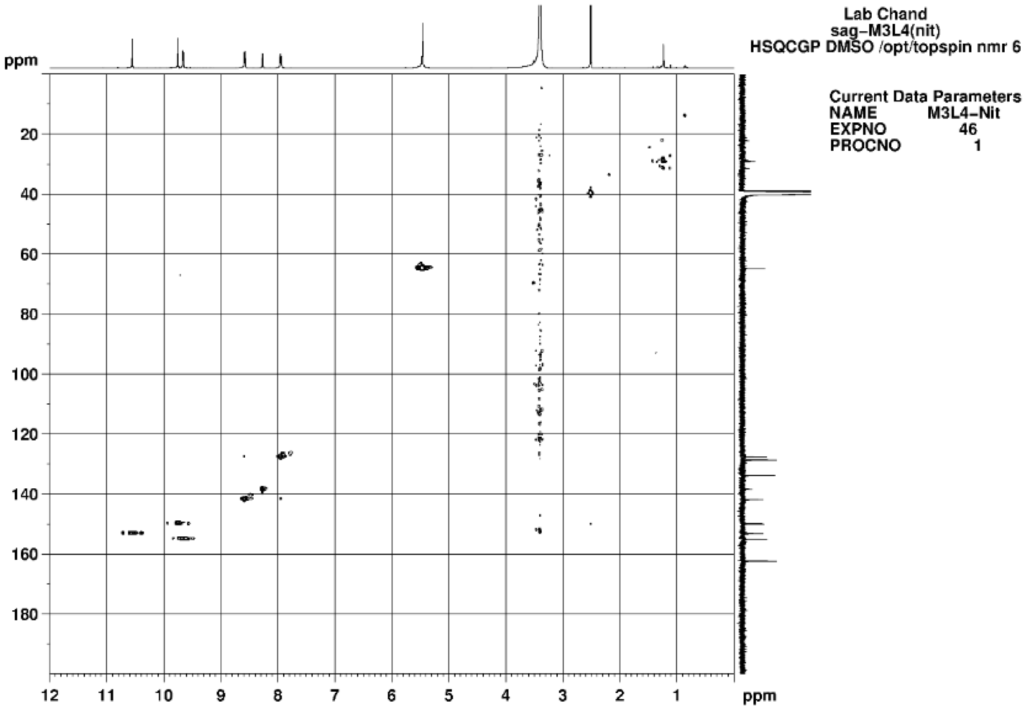

Figure S40a: C-H COSY (500 MHz, DMSO-d_6, 300 K) for $[(NO_3)_2@Pd_3(\mathbf{L1})_4](NO_3)_4$, **4a**.

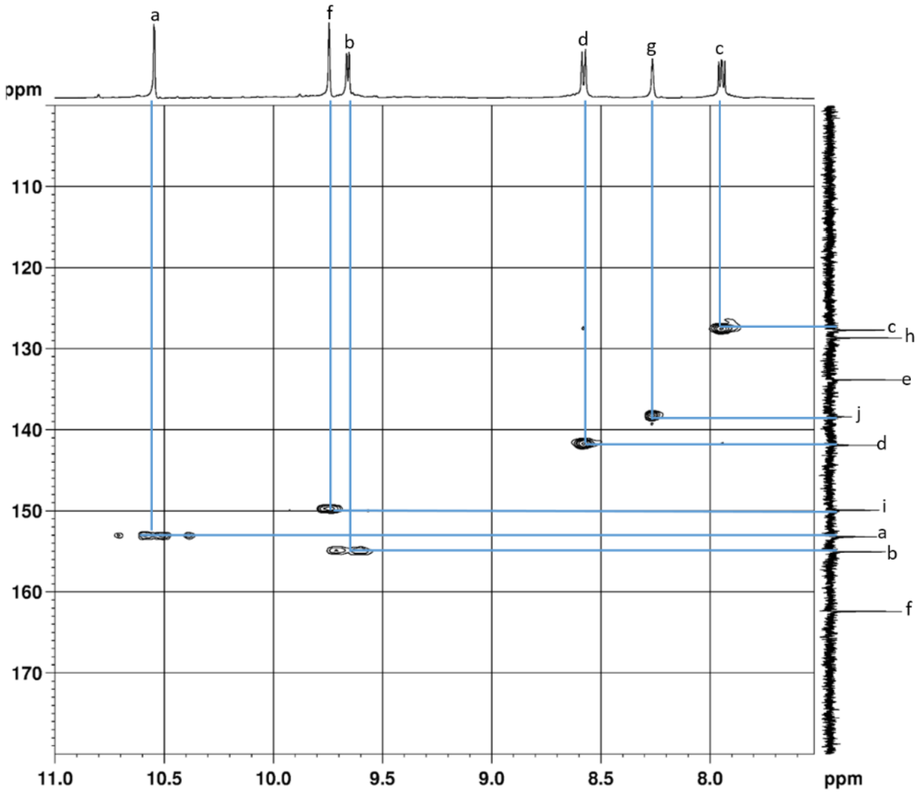

Figure S40b: C-H COSY expansion (500 MHz, DMSO-d_6, 300 K) for $[(NO_3)_2@Pd_3(\mathbf{L1})_4](NO_3)_4$, **4a**.

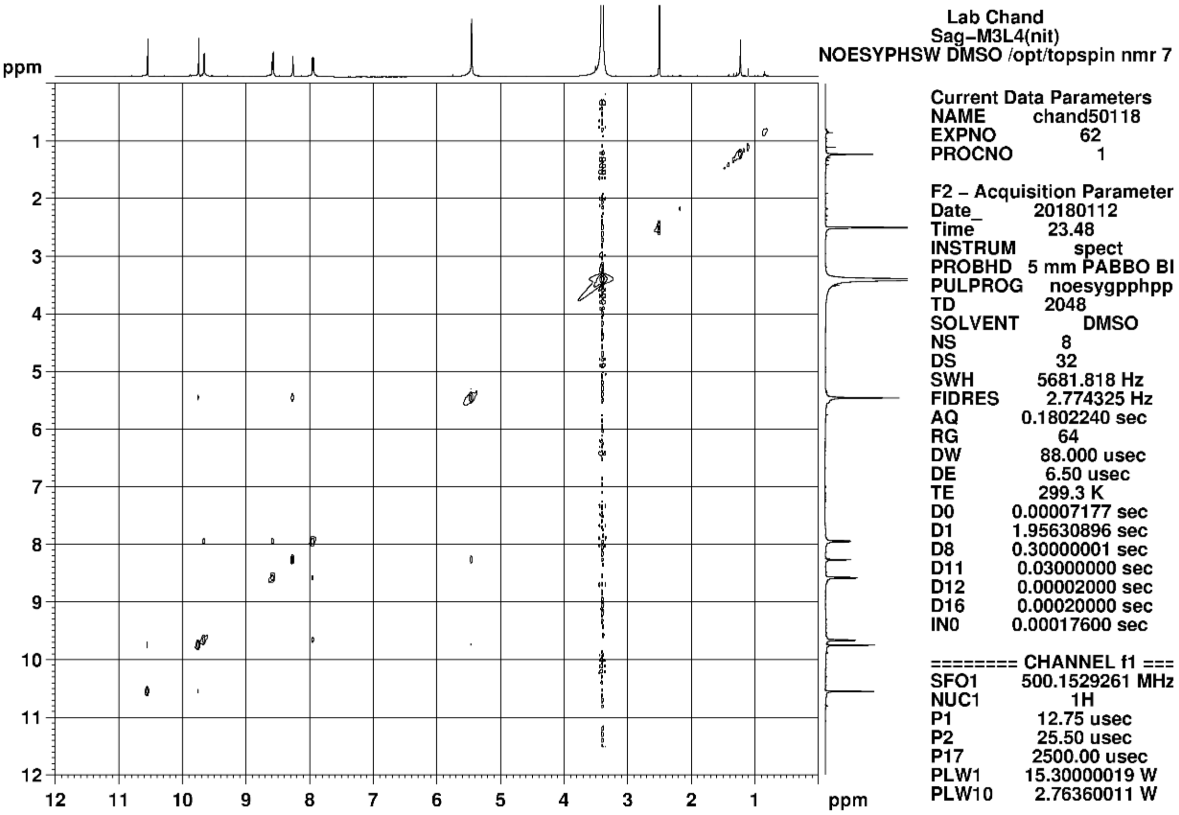

Figure S41a: NOESY (500 MHz, DMSO-d_6, 300 K) for $[(NO_3)_2@Pd_3(\mathbf{L1})_4](NO_3)_4$, **4a**.

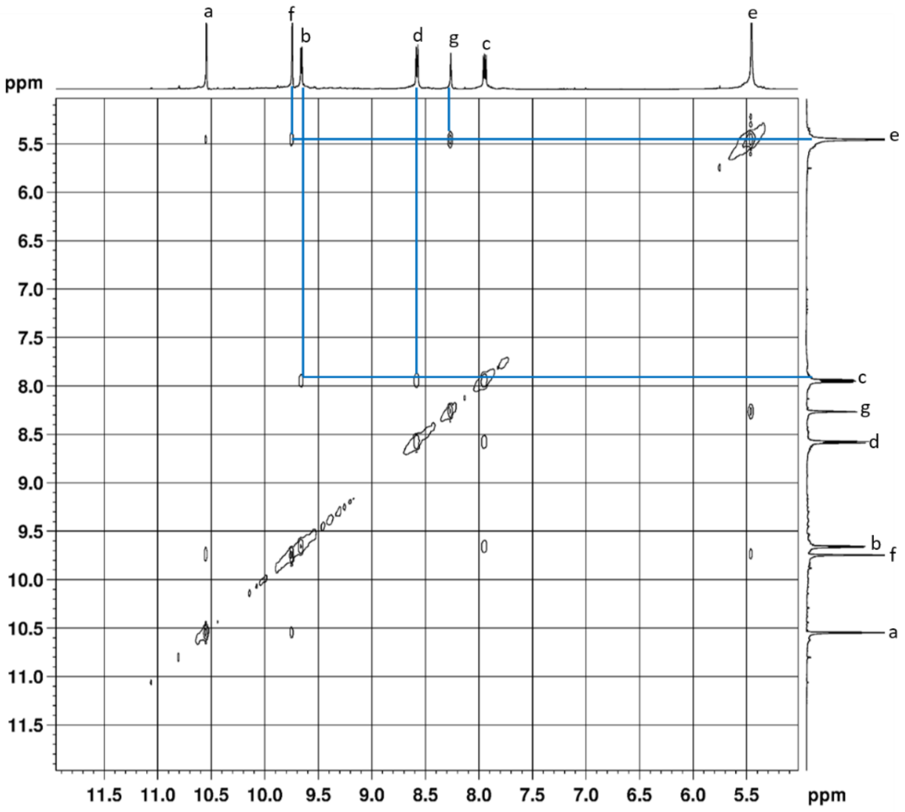

Figure S41b: NOESY expansion (500 MHz, DMSO-d_6, 300 K) for $[(NO_3)_2@Pd_3(\mathbf{L1})_4](NO_3)_4$, **4a**.

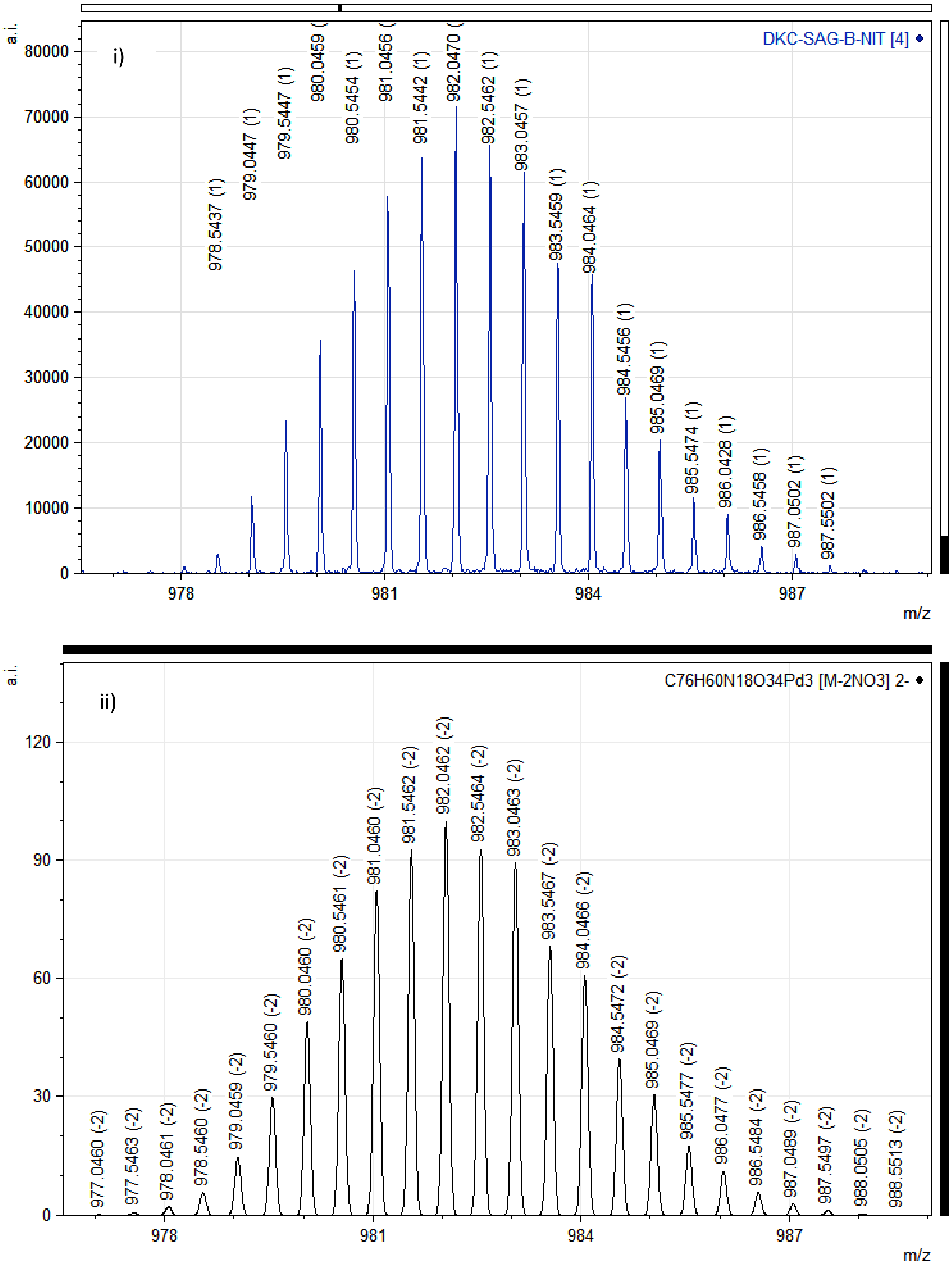

Figure S42a: ESIMS, isotopic pattern for [**4a** − $2NO_3$]$^{2+}$ i) experimental and ii) theoretical.

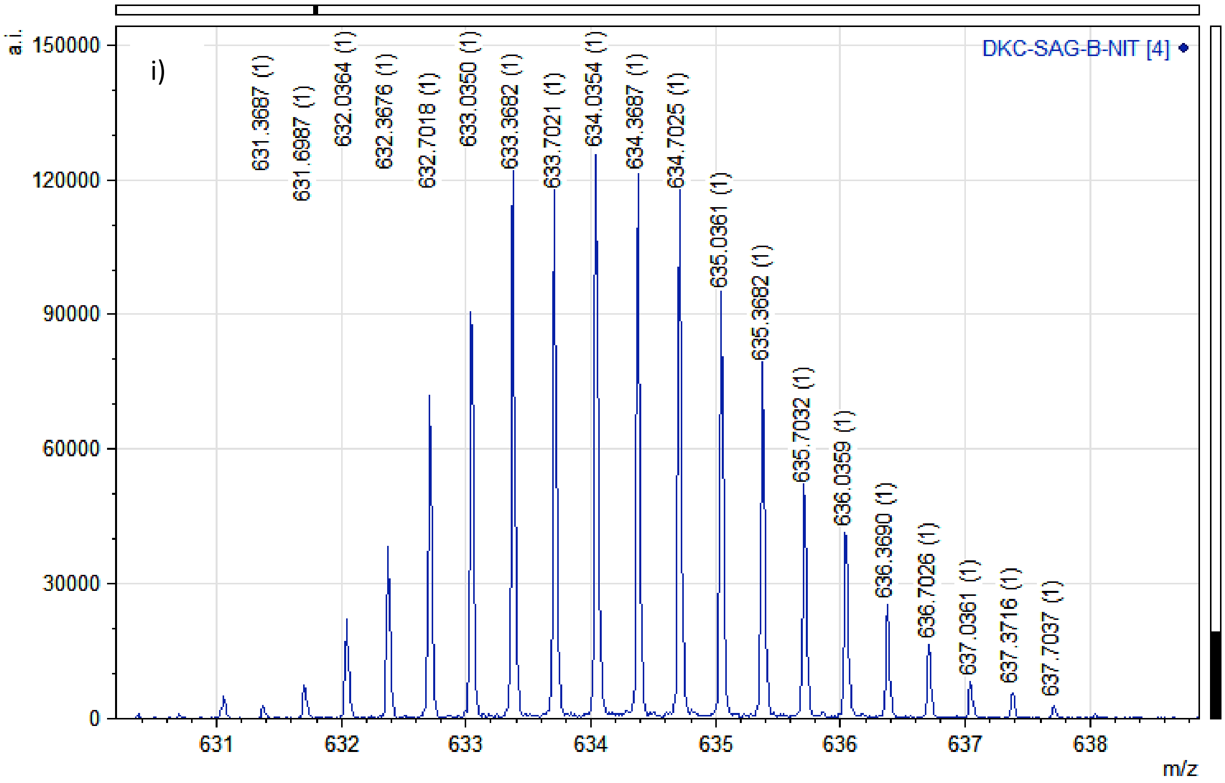

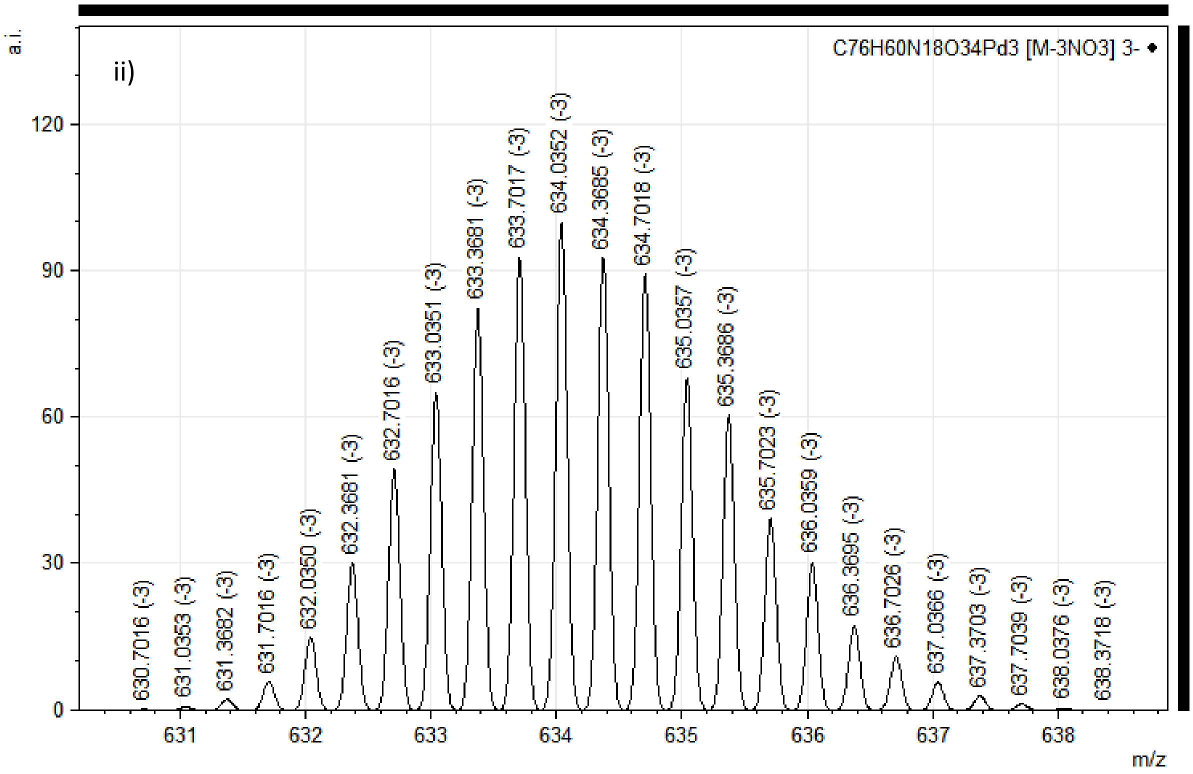

Figure S42b: ESIMS, isotopic pattern for [**4a** – $3NO_3]^{3+}$ i) experimental and ii) theoretical.

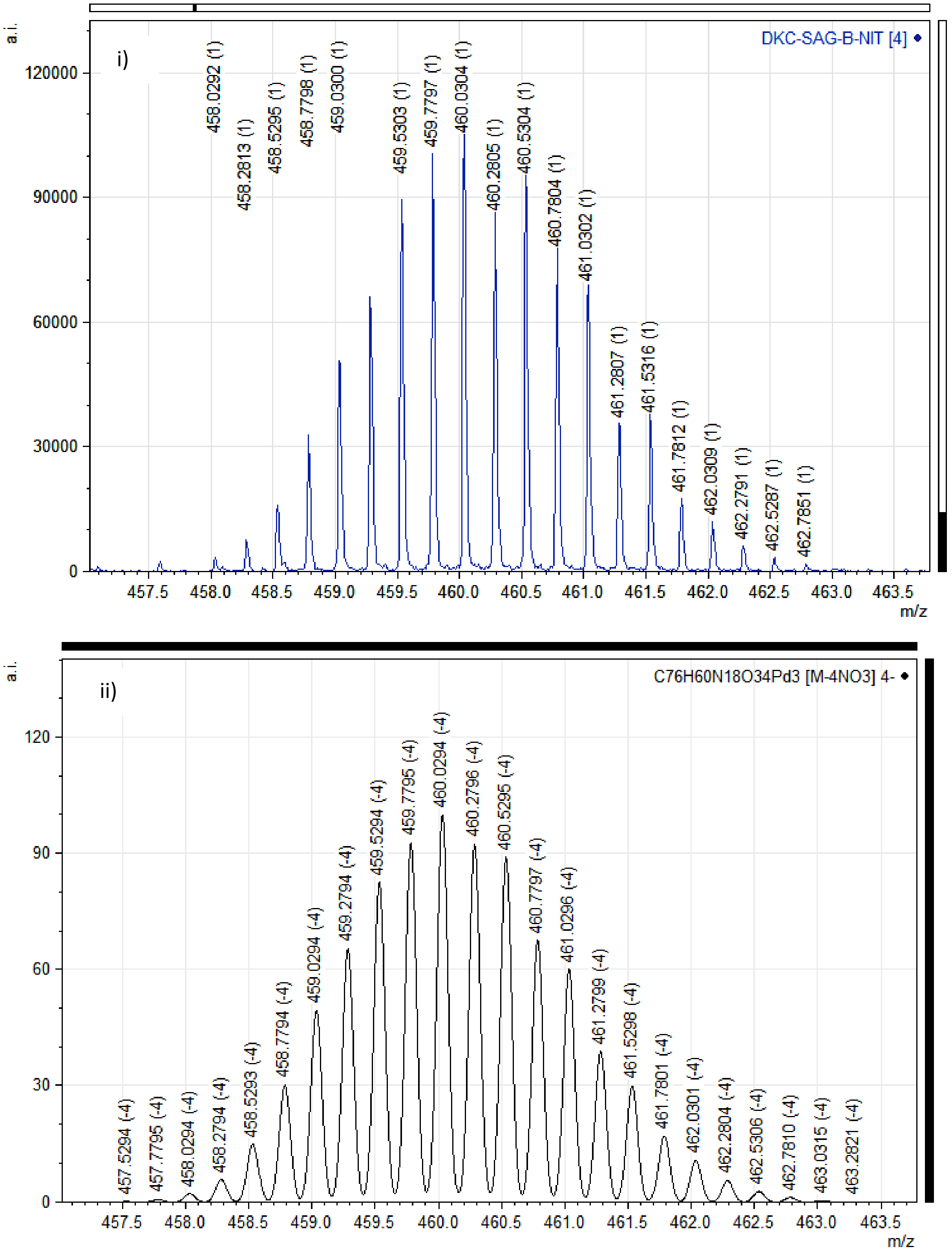

Figure S42c: ESIMS, isotopic pattern for [**4a** − $4NO_3]^{4+}$ i) experimental and ii) theoretical.

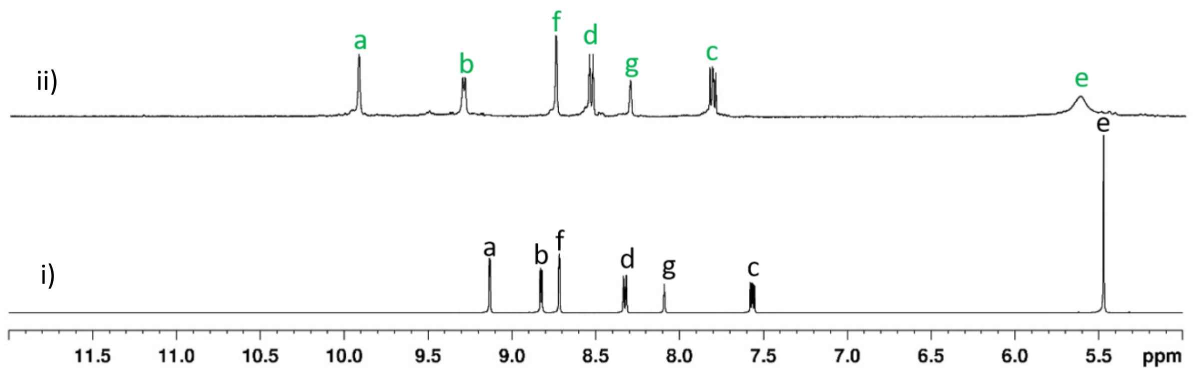

Figure S43: Partial ^{1}H NMR spectra (400 MHz, DMSO-d_6, 300 K) for (i) ligand **L1**; (ii) a solution of [Pd(**L1**)$_2$](NO$_3$)$_2$, **3a** after standing for 15 days.

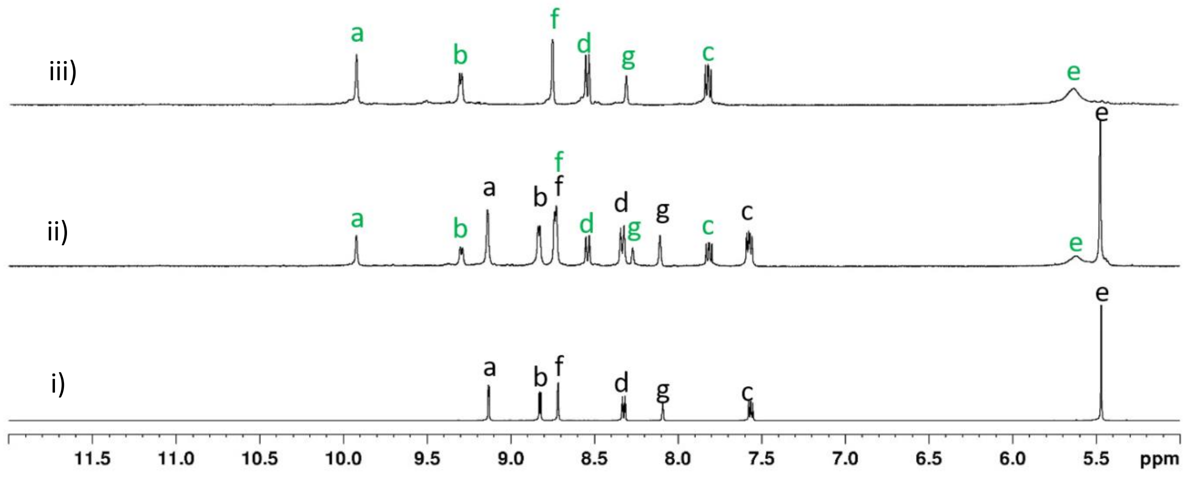

Figure S44: Partial ^{1}H NMR spectra (400 MHz, DMSO-d_6, 300 K) for (i) ligand **L1**; (ii) a solution of [Pd(**L1**)$_2$](NO$_3$)$_2$, **3a** after heating at 90 ºC 24 h (**3a** labelled in green and free ligand **L1** in black) (iii) pure [Pd(**L1**)$_2$](NO$_3$)$_2$, **3a** for comparison.

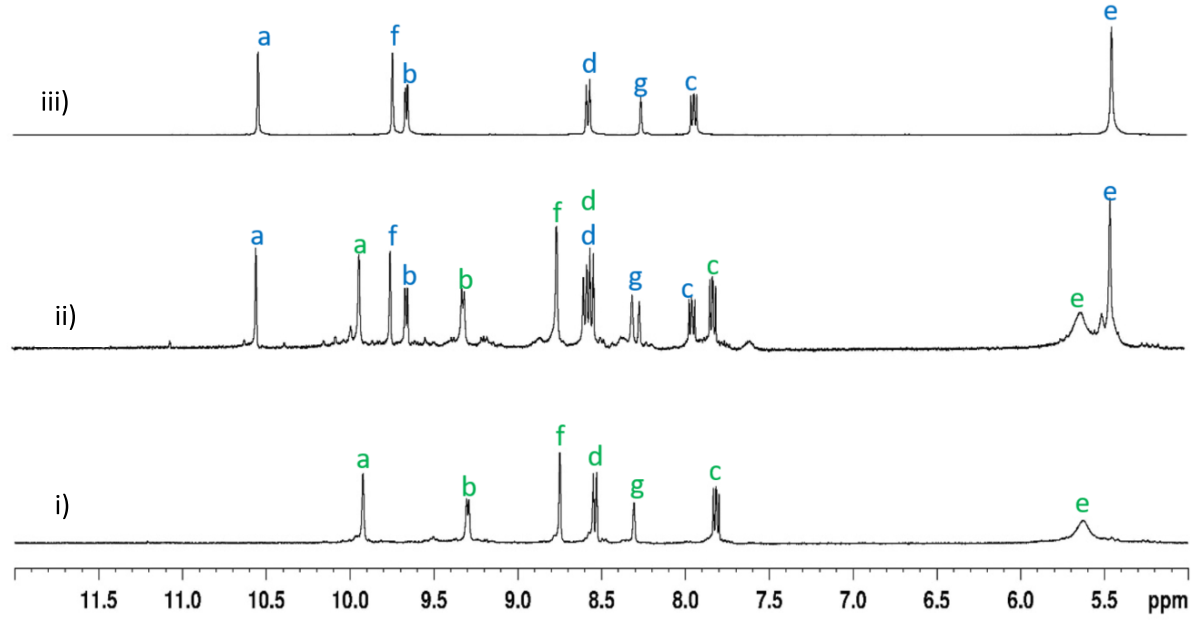

Figure S45: Partial ^{1}H NMR spectra (400 MHz, DMSO-d_6, 300 K) for (i) complex $[Pd_1(\mathbf{L1})_2](NO_3)_2$, **3a**; (ii) Monitoring formation of complex $[Pd_1(\mathbf{L1})_2](NO_3)_2$, **3a** (labelled in green) and $[(NO_3)_2@Pd_3(\mathbf{L1})_4](NO_3)_4$, **4a** (labelled in blue) by combining $Pd(NO_3)_2$ with **3a** and recorded after 10 min (iii) exclusive formation of $[(NO_3)_2@Pd_3(\mathbf{L1})_4](NO_3)_4$, **4a** after 20 min.

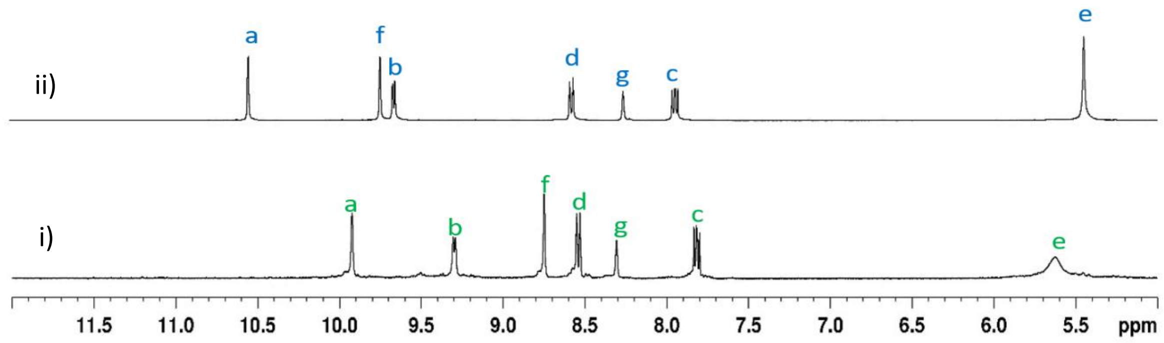

Figure S46: Partial ^{1}H NMR spectra (400 MHz, DMSO-d_6, 300 K) for (i) complex $[Pd_1(\mathbf{L1})_2](NO_3)_2$, **3a**; (ii) exclusive formation of $[(NO_3)_2@Pd_3(\mathbf{L1})_4](NO_3)_4$, **4a** by heating a mixture of $Pd(NO_3)_2$ and **3a** at 90 °C.

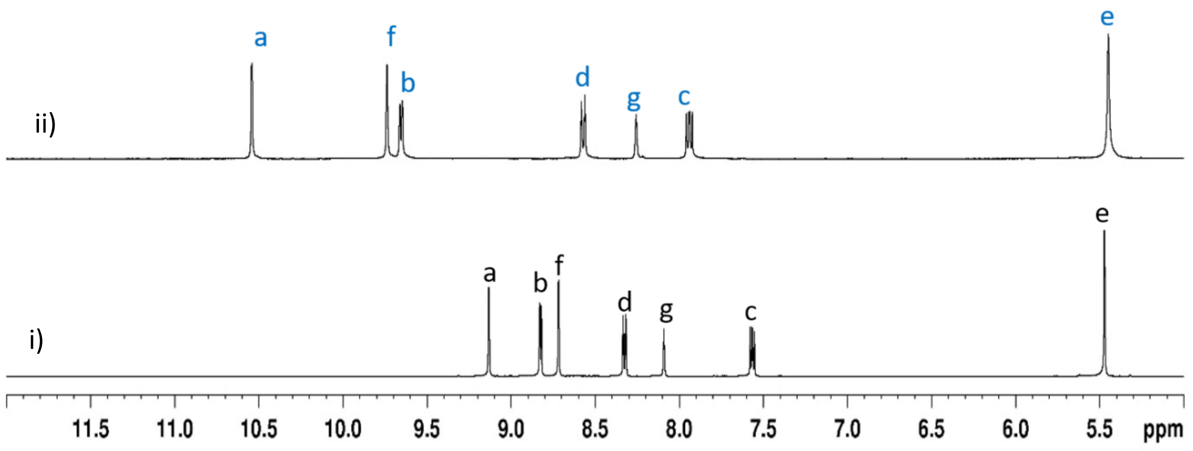

Figure S47: Partial ^{1}H NMR spectra (400 MHz, DMSO-d_6, 300 K) for (i) ligand **L1**; (ii) $[(NO_3)_2@Pd_3(\mathbf{L1})_4](NO_3)_4$, **4a** after standing for15 days.

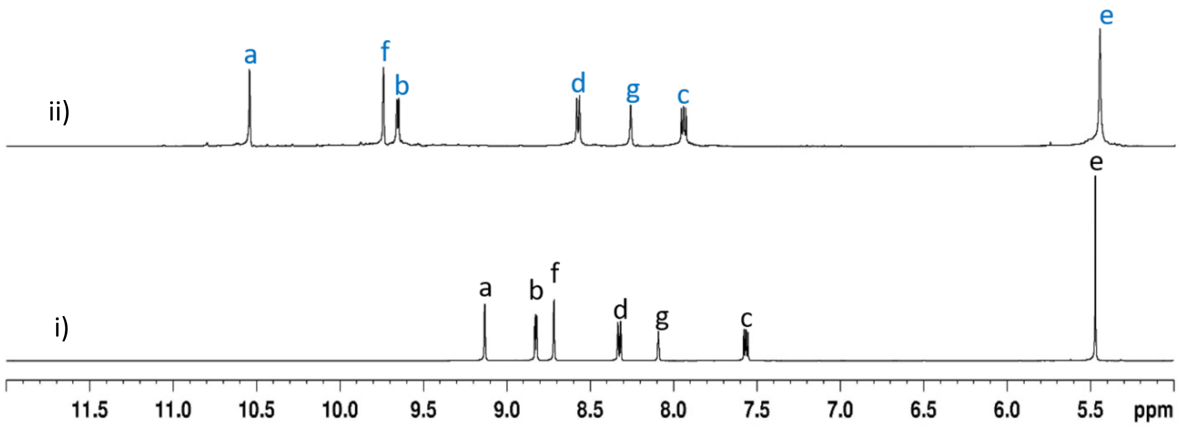

Figure S48: Partial ^{1}H NMR spectra (400 MHz, DMSO-d_6, 300 K) for (i) ligand **L1**; (ii) a solution of $[(NO_3)_2@Pd_3(\mathbf{L1})_4](NO_3)_4$, **4a** heated at 90 ºC for 5 days showing no change.

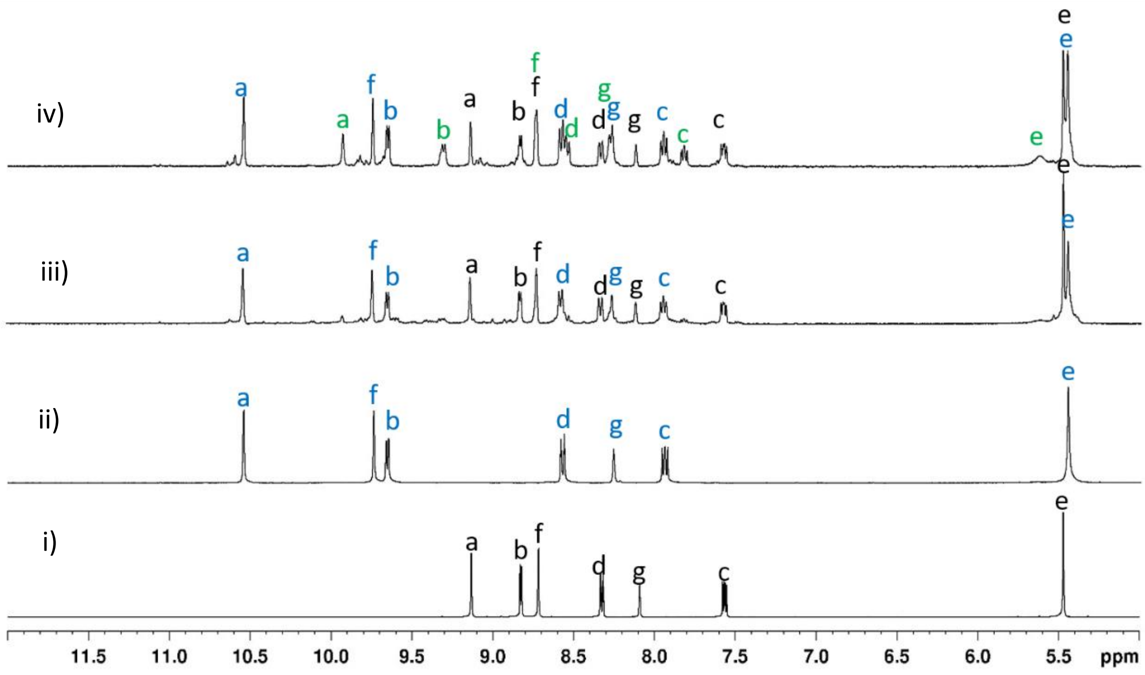

Figure S49: Partial ^{1}H NMR spectra (400 MHz, DMSO-d_6, 300 K) for (i) ligand **L1** ii) $[(NO_3)_2@Pd_3($**L1**$)_4](NO_3)_4$, **4a**; (iii) a mixture of pre-prepared $[(NO_3)_2@Pd_3($**L1**$)_4](NO_3)_4$, **4a** (labelled in blue) and ligand **L1** (iv) minor amount of $[Pd_1($**L1**$)_2](NO_3)_2$, **3a** (labelled in green) generated from a mixture of **4a** and **L1** after stirring for 20 min.

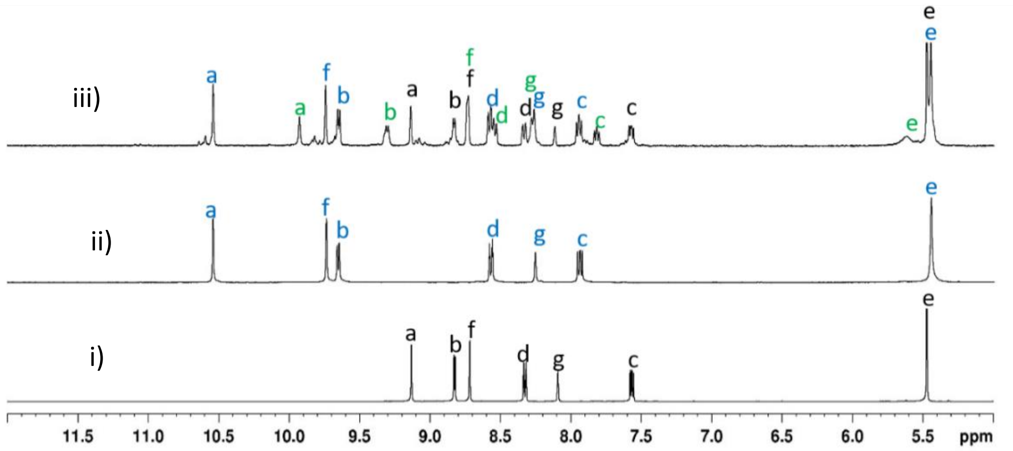

Figure S50: Partial ^{1}H NMR spectra (400 MHz, DMSO-d_6, 300 K) for (i) ligand **L1** ii) $[(NO_3)_2@Pd_3($**L1**$)_4](NO_3)_4$, **4a**; (iii) a mixture of pre-prepared $[(NO_3)_2@Pd_3($**L1**$)_4](NO_3)_4$, **4a** (labelled in blue) and ligand **L1** that generated minor amount of $[Pd_1($**L1**$)_2](NO_3)_2$, **3a** (labelled in green) after stirring at 90 °C for 5 min.

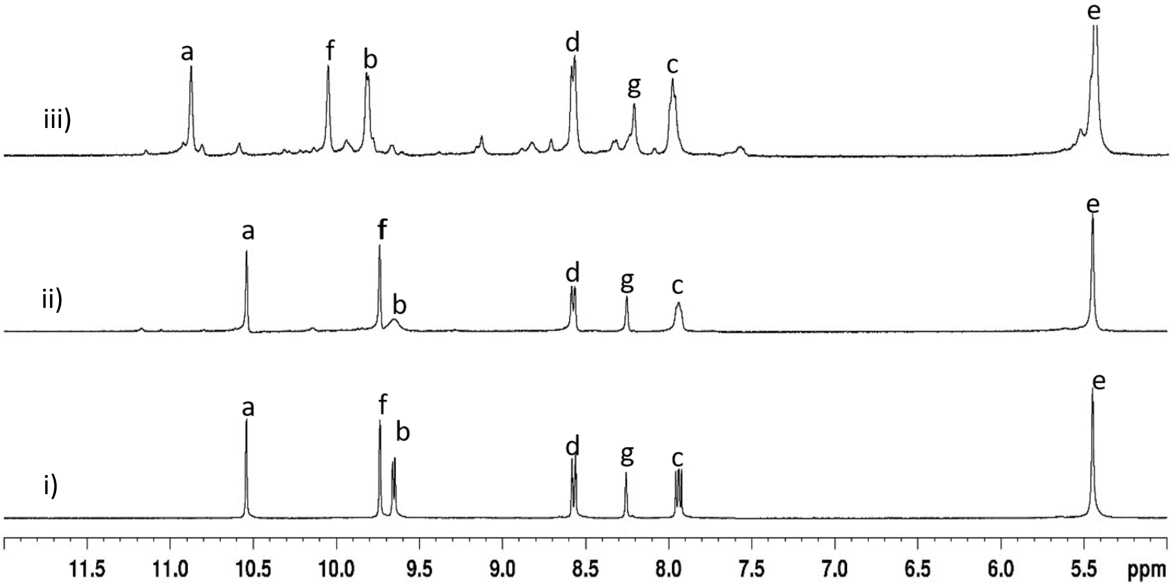

Figure S51: 400 MHz ^{1}H NMR spectra in DMSO-d_6 for in situ prepared (i) $[(NO_3)_2@Pd_3(\mathbf{L1})_4](NO_3)_4$, **4a**; (ii) a sample prepared by addition of 1.5 equivalent of *n*-Bu_4NF to $[(NO_3)_2@Pd_3(\mathbf{L1})_4](NO_3)_4$, **4a** (iii) $[(F)_2@Pd_3(\mathbf{L1})_4](NO_3)_4$, **5a** obtained from a 3:1 combination of *n*-Bu_4NF with **4a**.

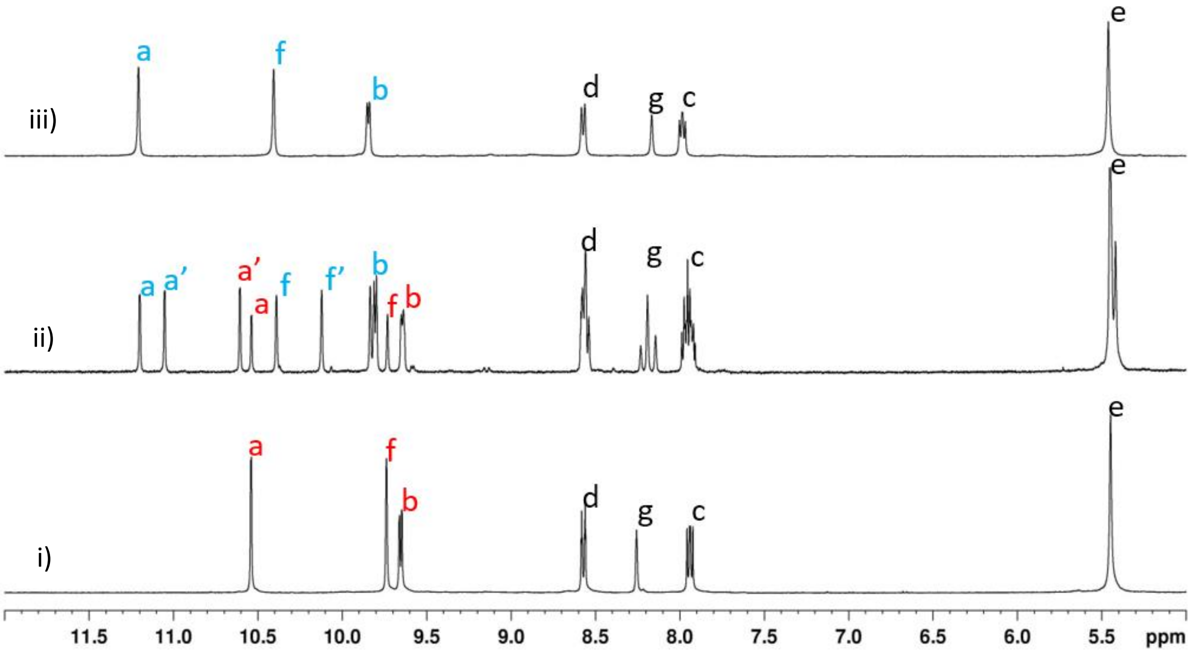

Figure S52: 400 MHz partial ^{1}H NMR spectra in DMSO-d_6 for in situ prepared (i) $[(NO_3)_2@Pd_3(\mathbf{L1})_4](NO_3)_4$, **4a**; (ii) a sample prepared by addition of 1.5 equivalent of *n*-Bu_4NCl to $[(NO_3)_2@Pd_3(\mathbf{L1})_4](NO_3)_4$, **4a** (iii) $[(Cl)_2@Pd_3(\mathbf{L1})_4](NO_3)_4$, **6a** obtained from a 3:1 combination of *n*-Bu_4NCl with **4a**.

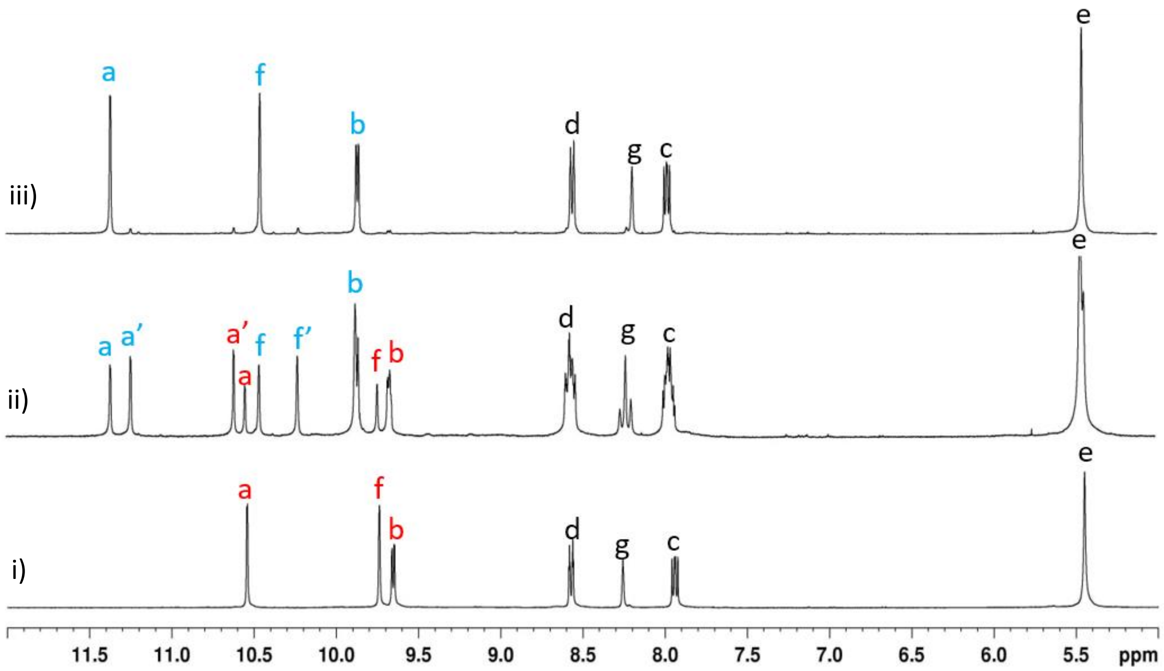

Figure S53: 400 MHz partial ^{1}H NMR spectra in DMSO-d_6 for in situ prepared (i) $[(NO_3)_2@Pd_3(\mathbf{L1})_4](NO_3)_4$, **4a**; (ii) a sample prepared by addition of 1.5 equivalent of *n*-Bu_4NBr to $[(NO_3)_2@Pd_3(\mathbf{L1})_4](NO_3)_4$, **4a** (iii) $[(Br)_2@Pd_3(\mathbf{L1})_4](NO_3)_4$, **7a** obtained from a 3:1 combination of *n*-Bu_4NBr with **4a**.

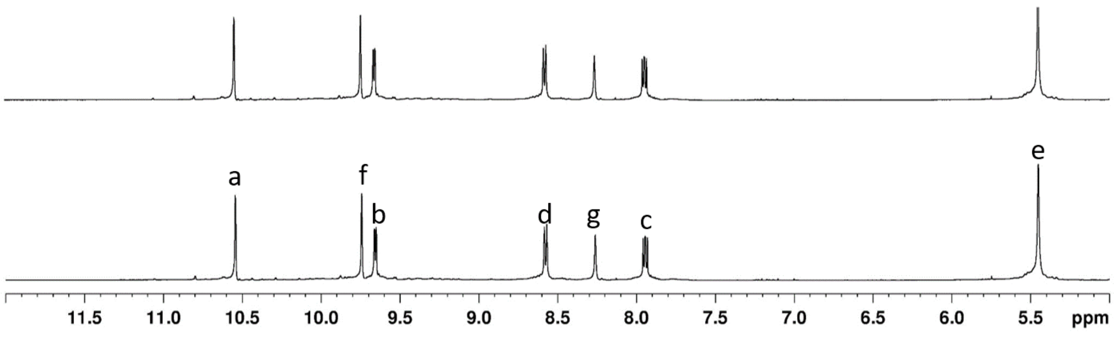

Figure S54: Partial ^{1}H NMR for complex $[(NO_3)_2@Pd_3(\mathbf{L1})_4](NO_3)_4$, **4a** and ii) a mixture of addition of TBAI **4a** (no changes).

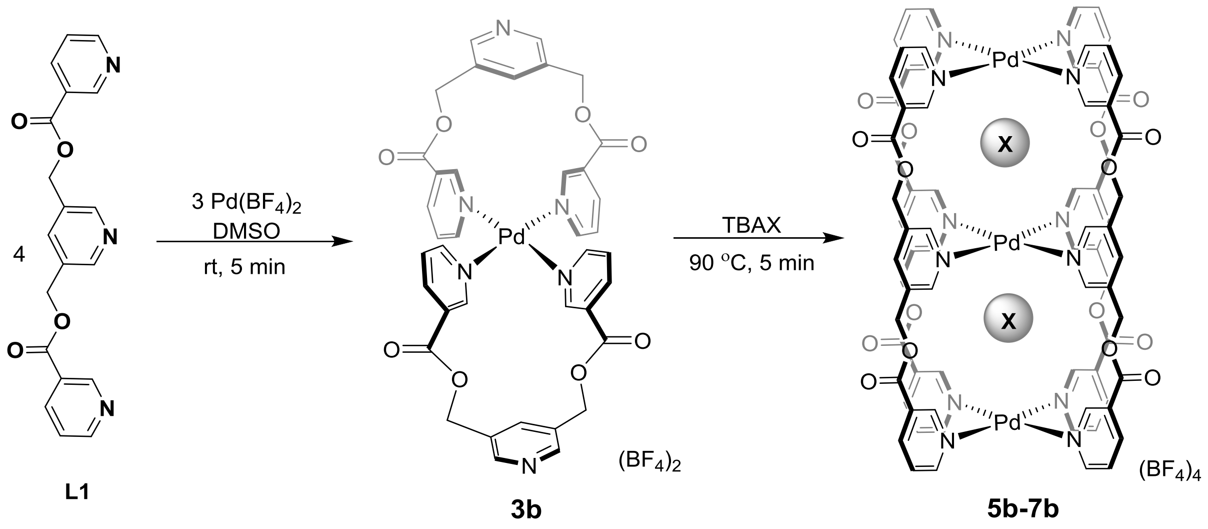

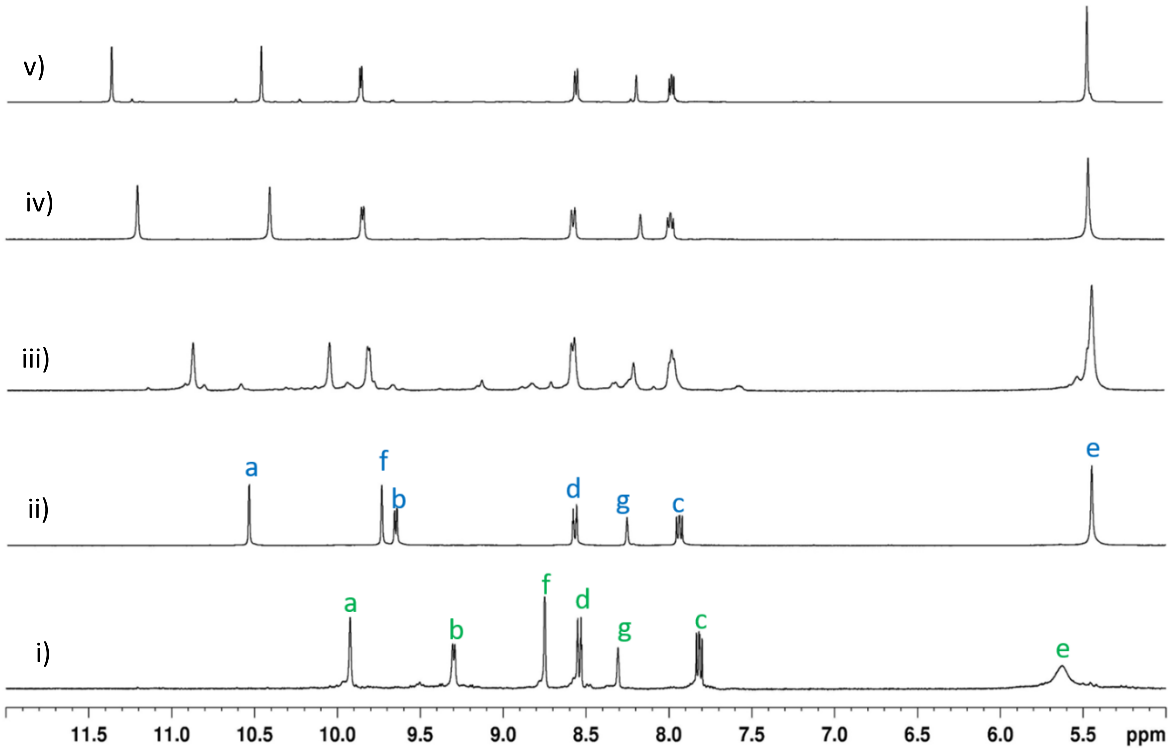

Figure S55: Partial ^{1}H NMR spectra (400 MHz, DMSO-d_6, 300 K) for (i) [Pd$_1$(**L1**)$_2$](BF$_4$)$_2$, **3b**; (ii) [(NO$_3$)$_2$@Pd$_3$(**L1**)$_4$](BF$_4$)$_4$, **4b**; (iii) [(F)$_2$@Pd$_3$(**L1**)$_4$](BF$_4$)$_4$, **5b**; (iv) [(Cl)$_2$@Pd$_3$(**L1**)$_4$](BF$_4$)$_4$, **6b** and (v) [(Br)$_2$@Pd$_3$(**L1**)$_4$](BF$_4$)$_4$, **7b**.

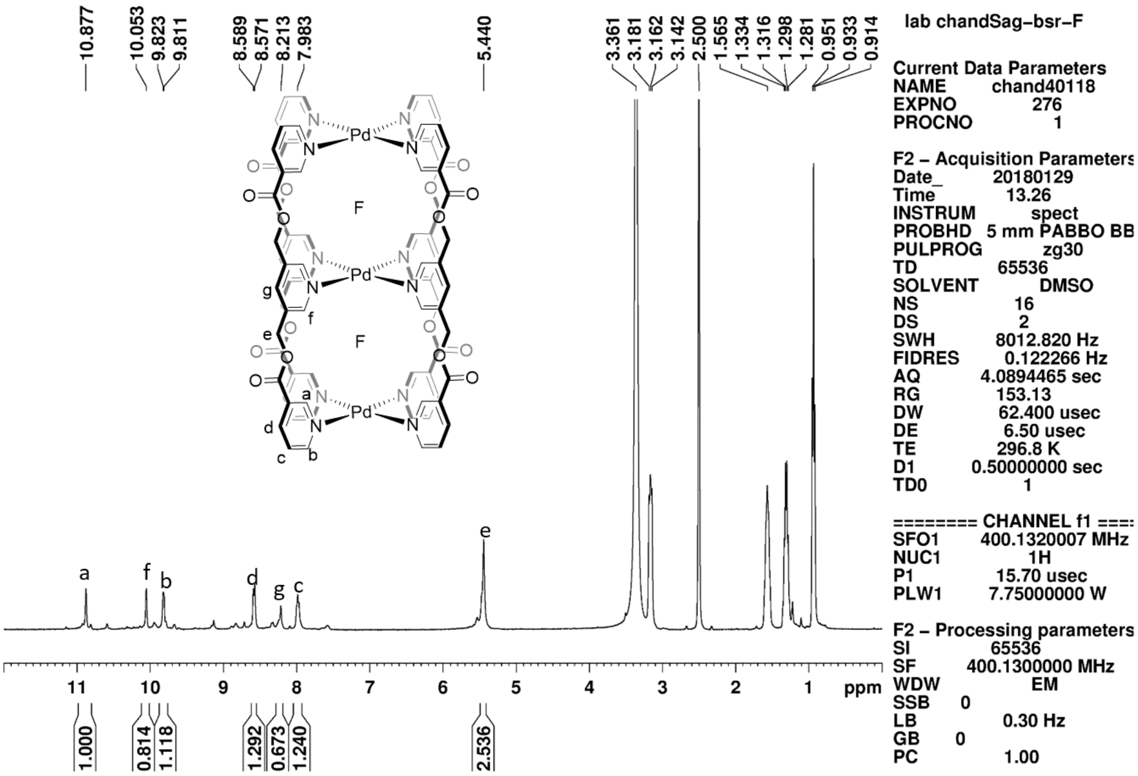

Figure S56: ^{1}H NMR (400 MHz, DMSO-d_6, 300 K) for **5a**.

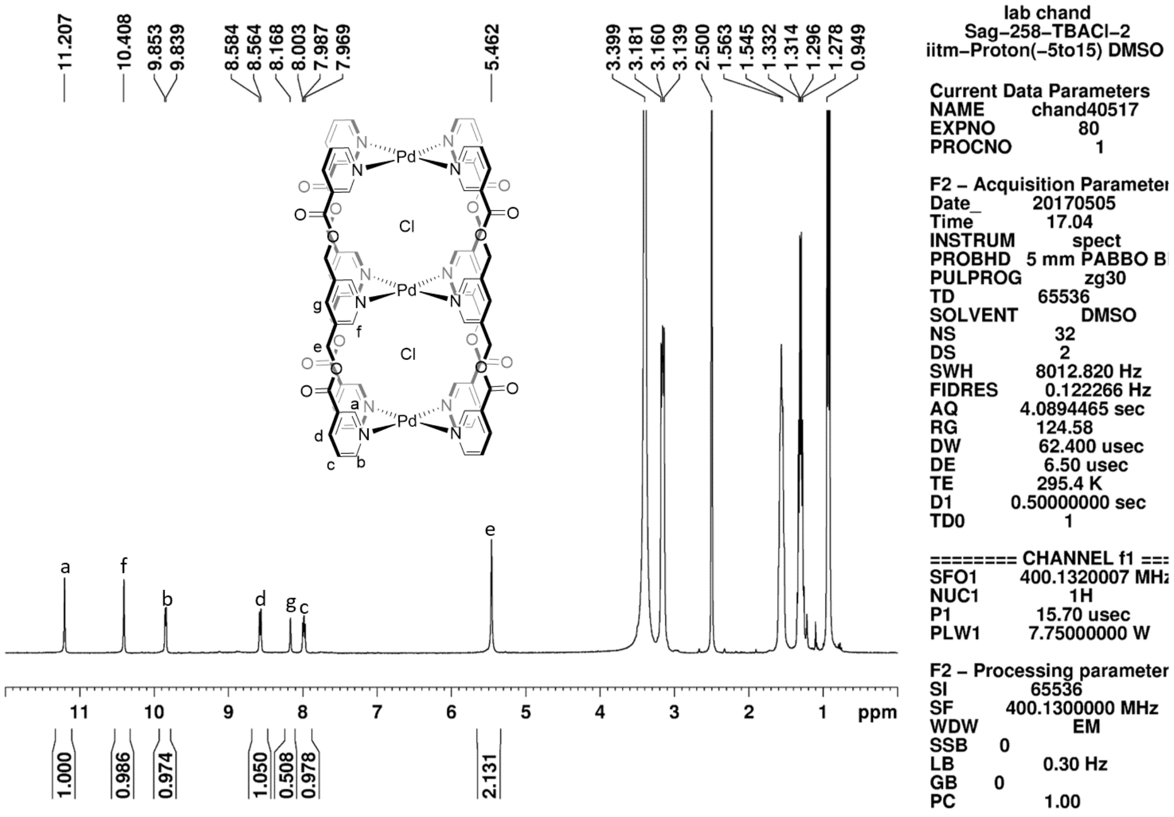

Figure S57: ^{1}H NMR (400 MHz, DMSO-d_6, 300 K) for **6a**.

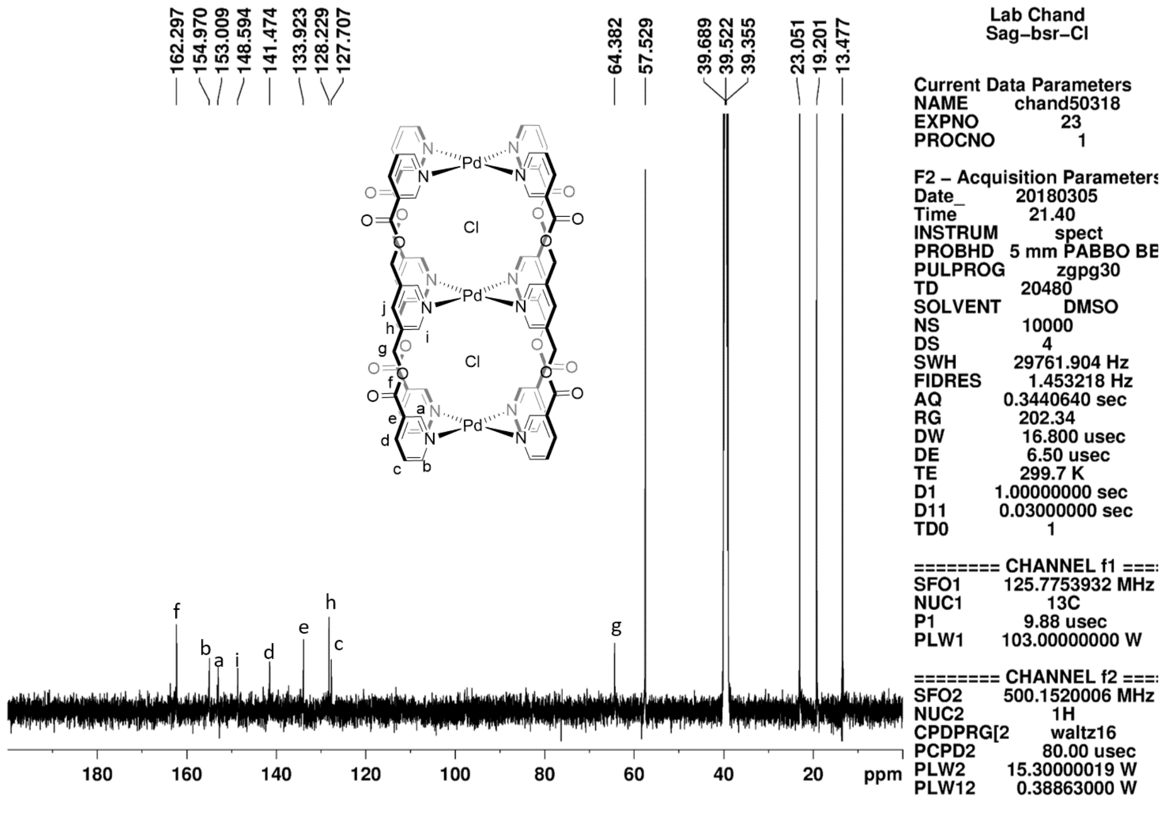

Figure S58: ^{13}C NMR (125 MHz, DMSO-d_6, 300 K) for **6a**.

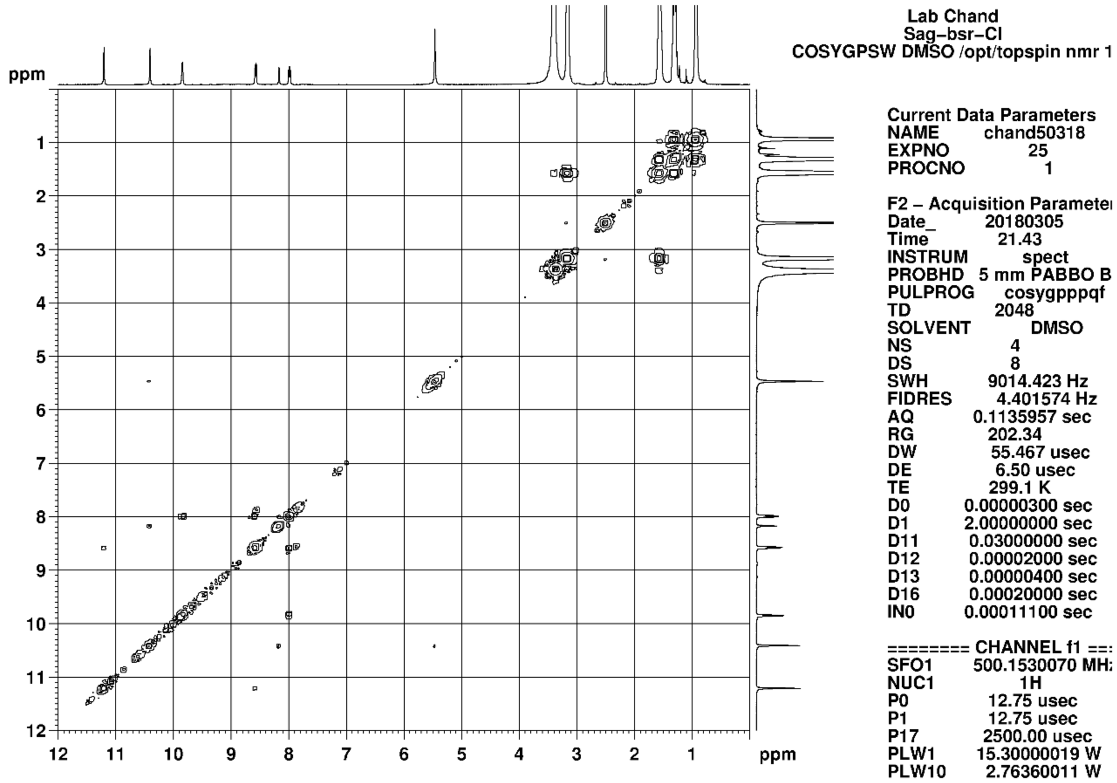

Figure S59a: H-H COSY (500 MHz, DMSO-d_6, 300 K) for **6a.**

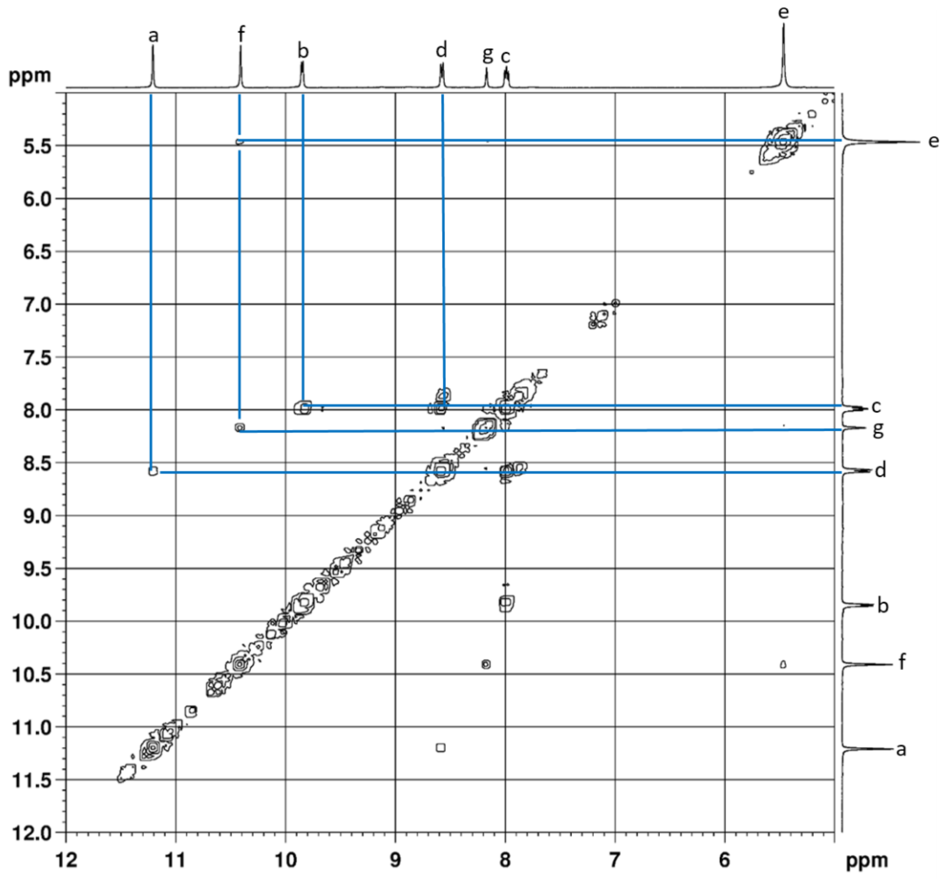

Figure S59b: H-H COSY expansion (500 MHz, DMSO-d_6, 300 K) for **6a**

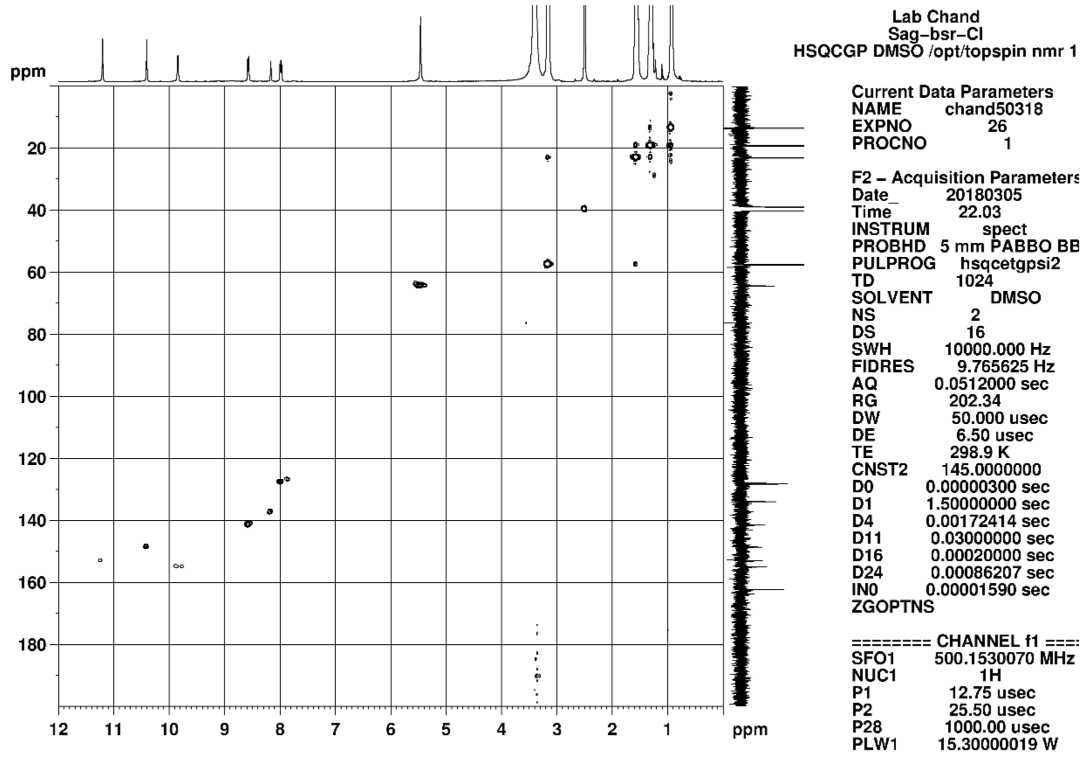

Figure S60a: C-H COSY (500 MHz, DMSO-d_6, 300 K) for **6a**

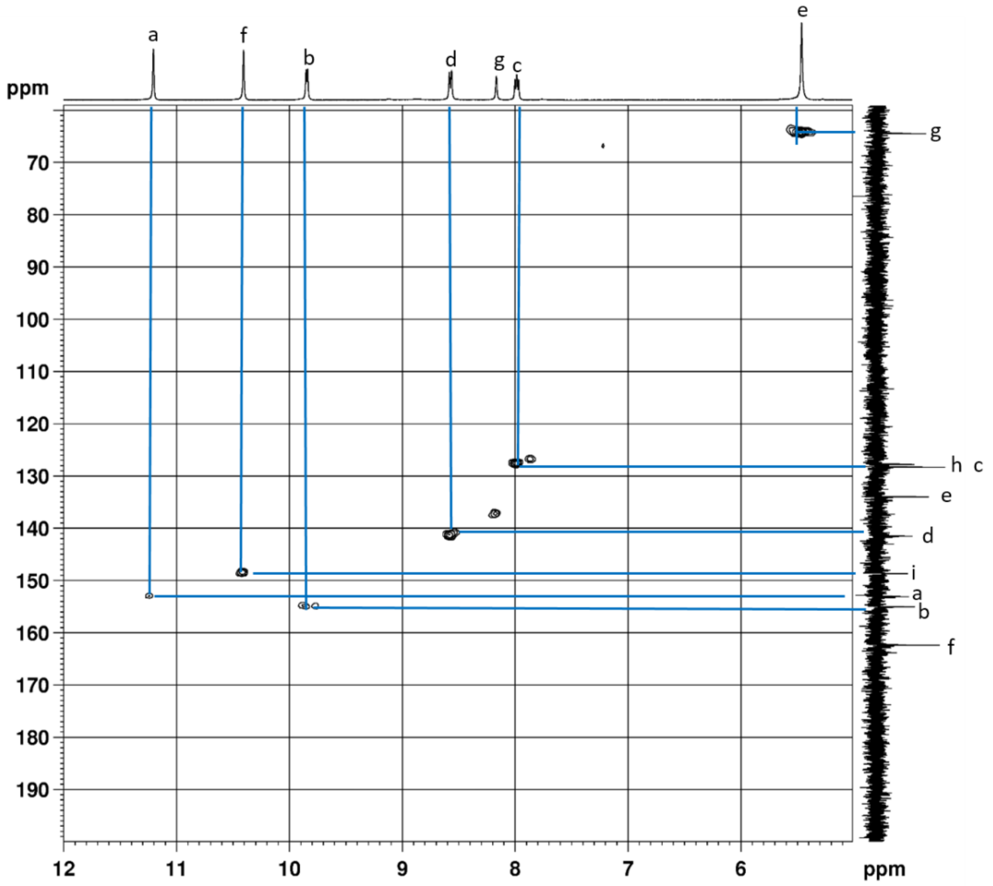

Figure S60b: C-H COSY expansion (500 MHz, DMSO-d_6, 300 K) for **6a**

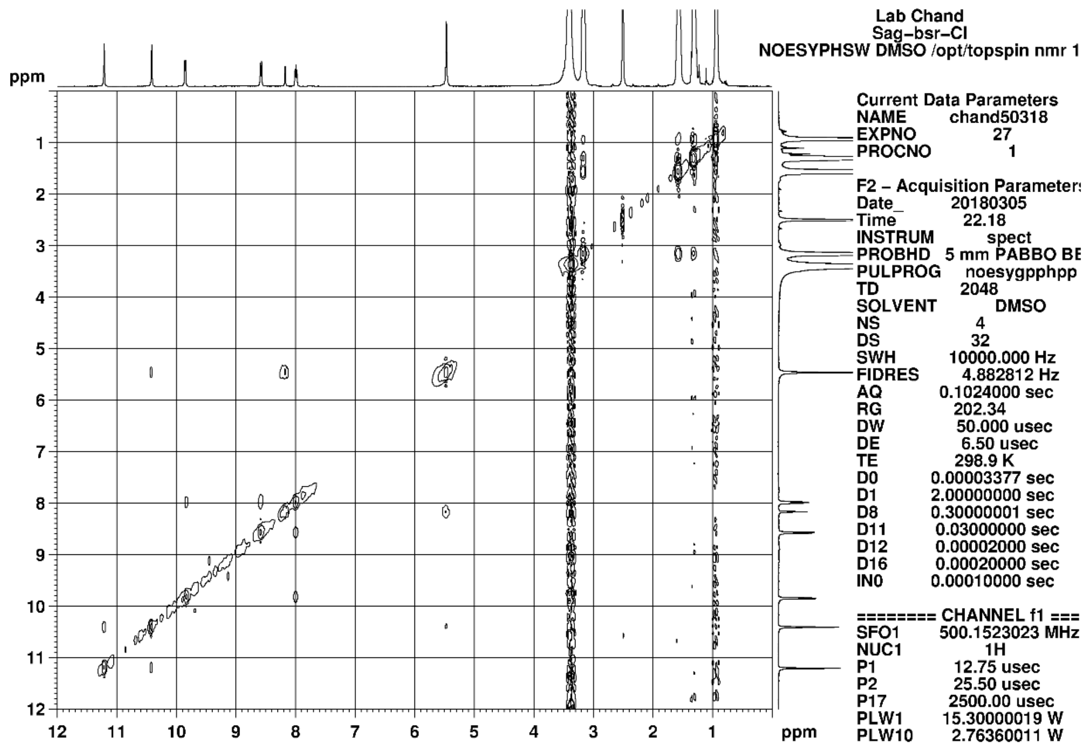

Figure S61a: NOESY (500 MHz, DMSO-d_6, 300 K) for **6a**

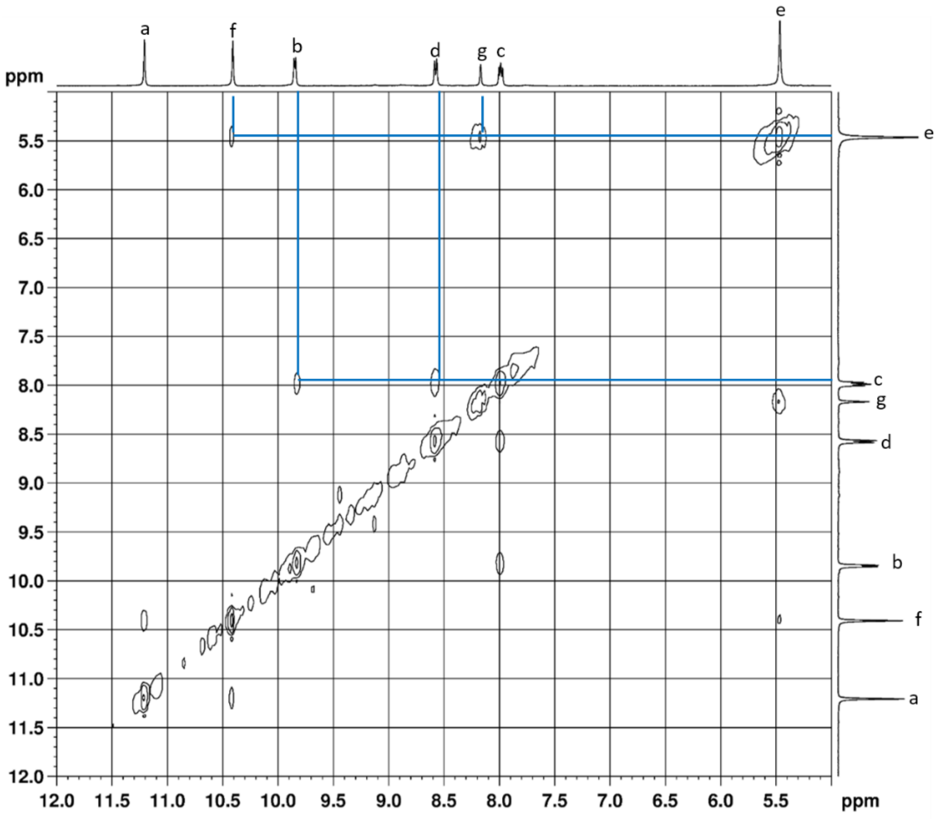

Figure S61b: NOESY expansion (500 MHz, DMSO-d_6, 300 K) for **6a**

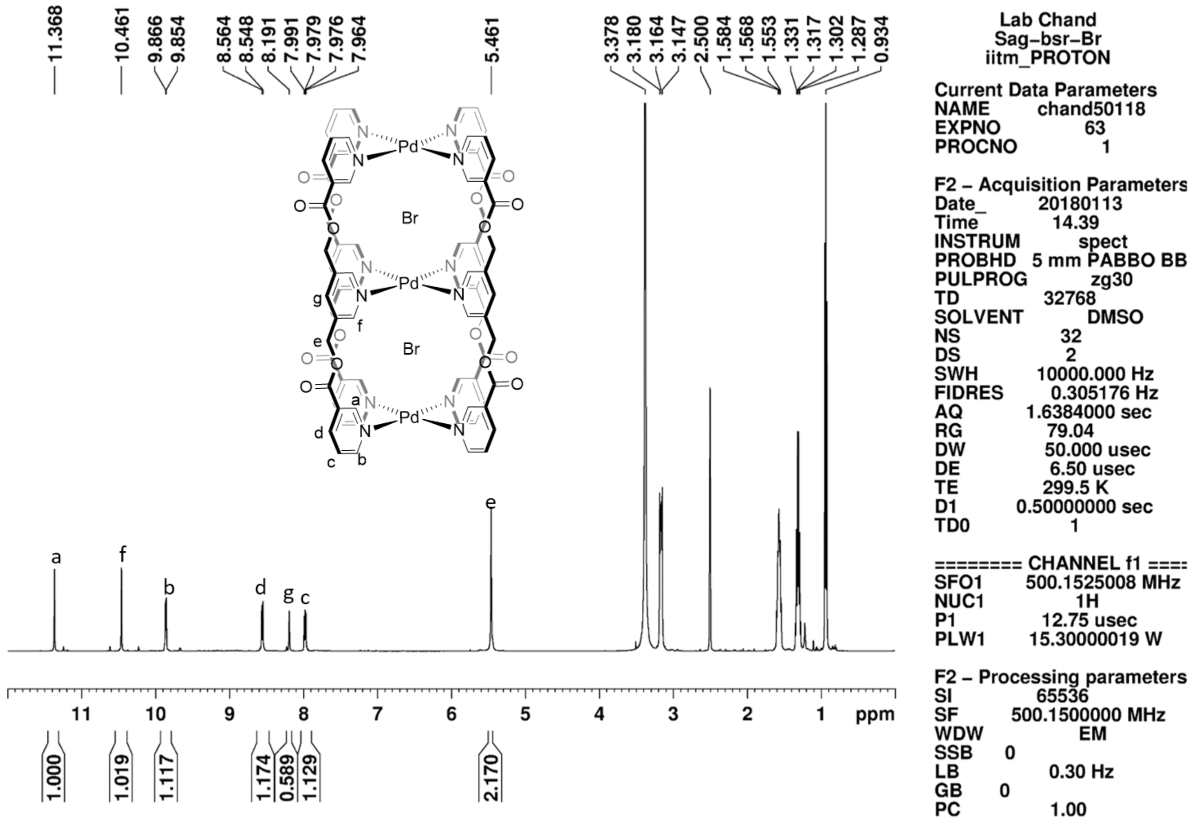

Figure S62: ^{1}H NMR (500 MHz, DMSO-d_6, 300 K) for **7a**.

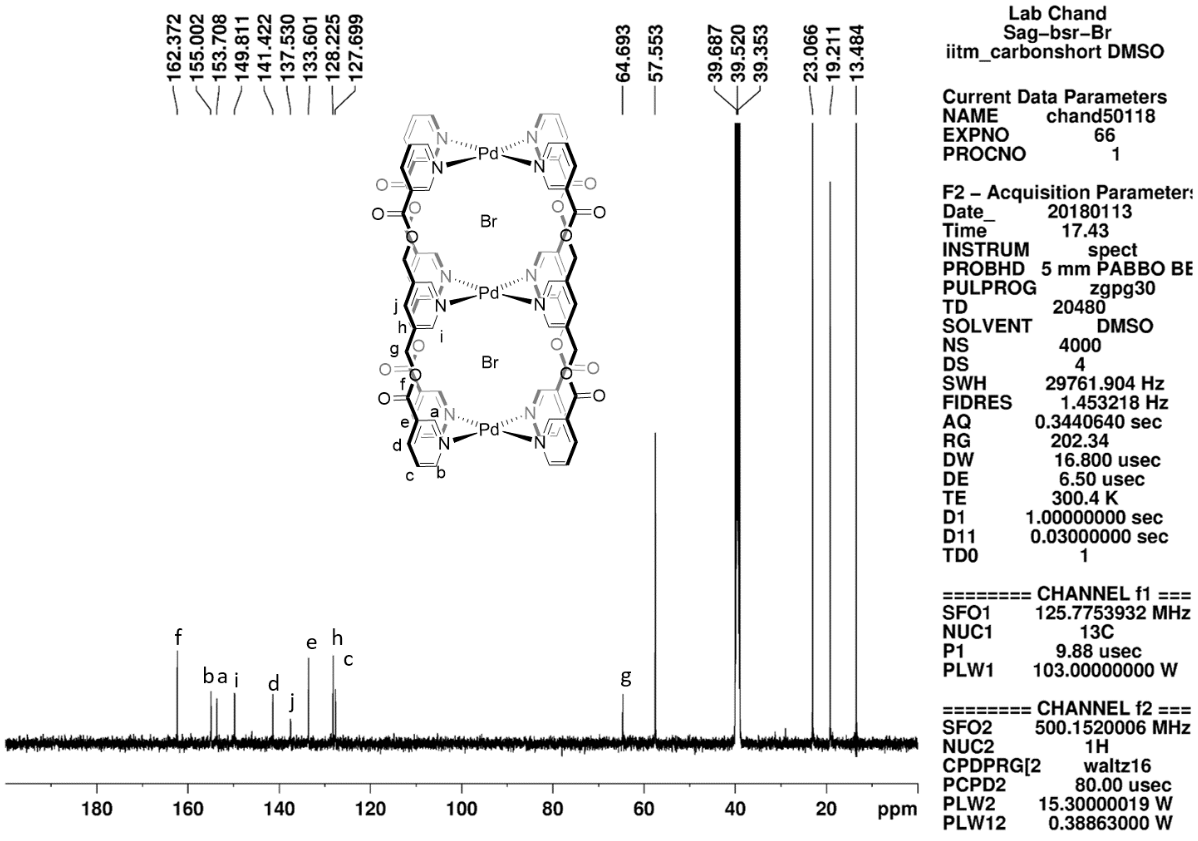

Figure S63: ^{13}C NMR (125 MHz, DMSO-d_6, 300 K) for **7a**.

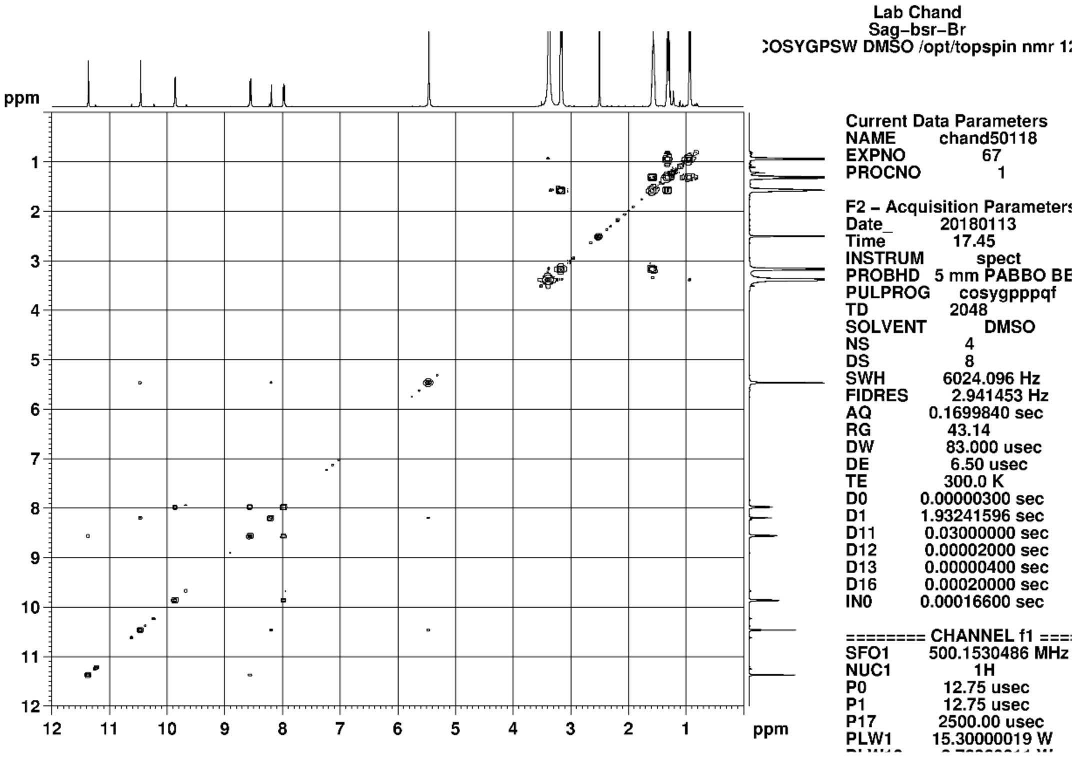

Figure S64a: H-H COSY (500 MHz, DMSO-d_6, 300 K) for **7a**.

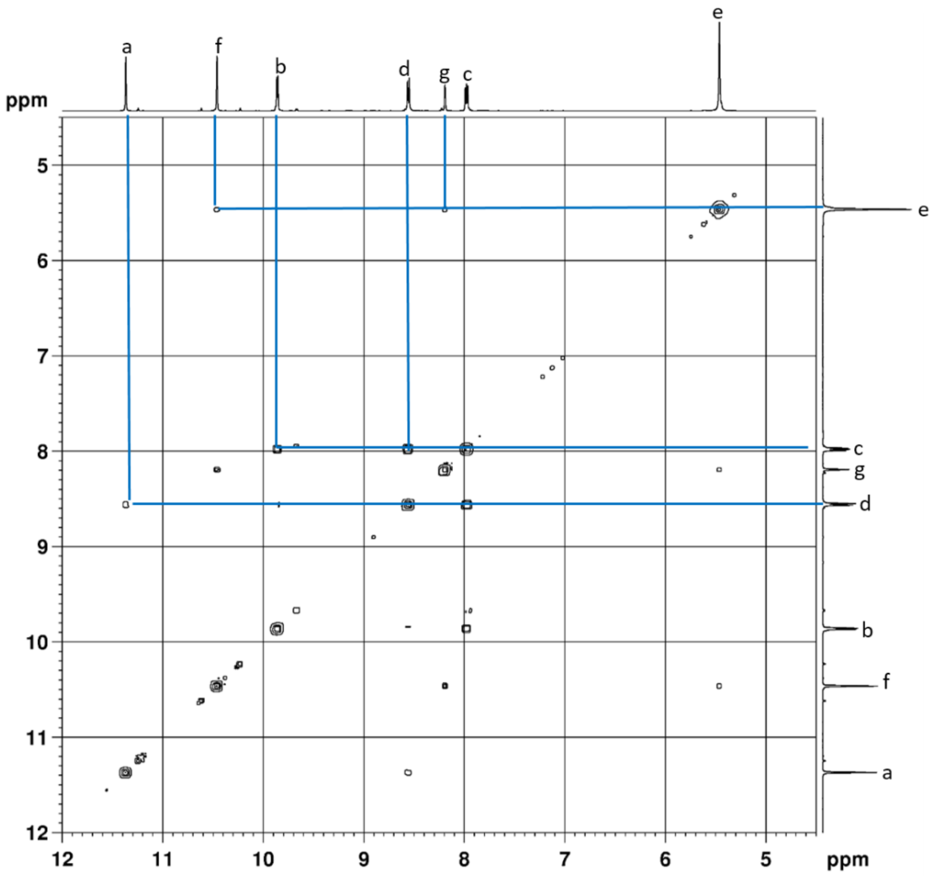

Figure S64b: H-H COSY expansion (500 MHz, DMSO-d_6, 300 K) for **7a**.

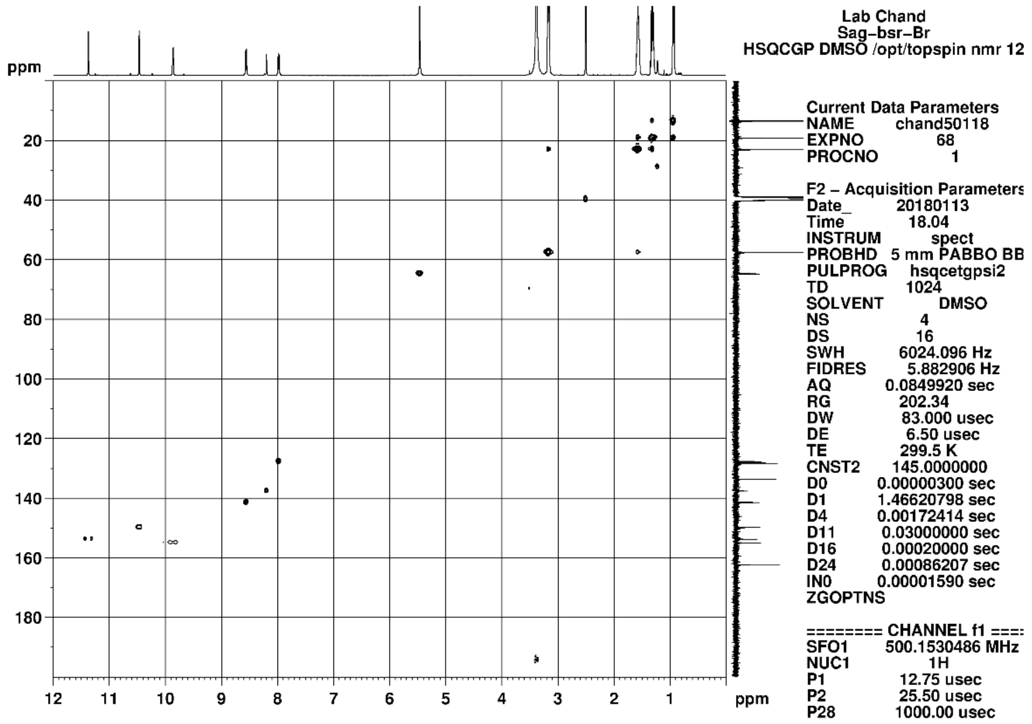

Figure S65a: C-H COSY (500 MHz, DMSO-d_6, 300 K) for **7a**.

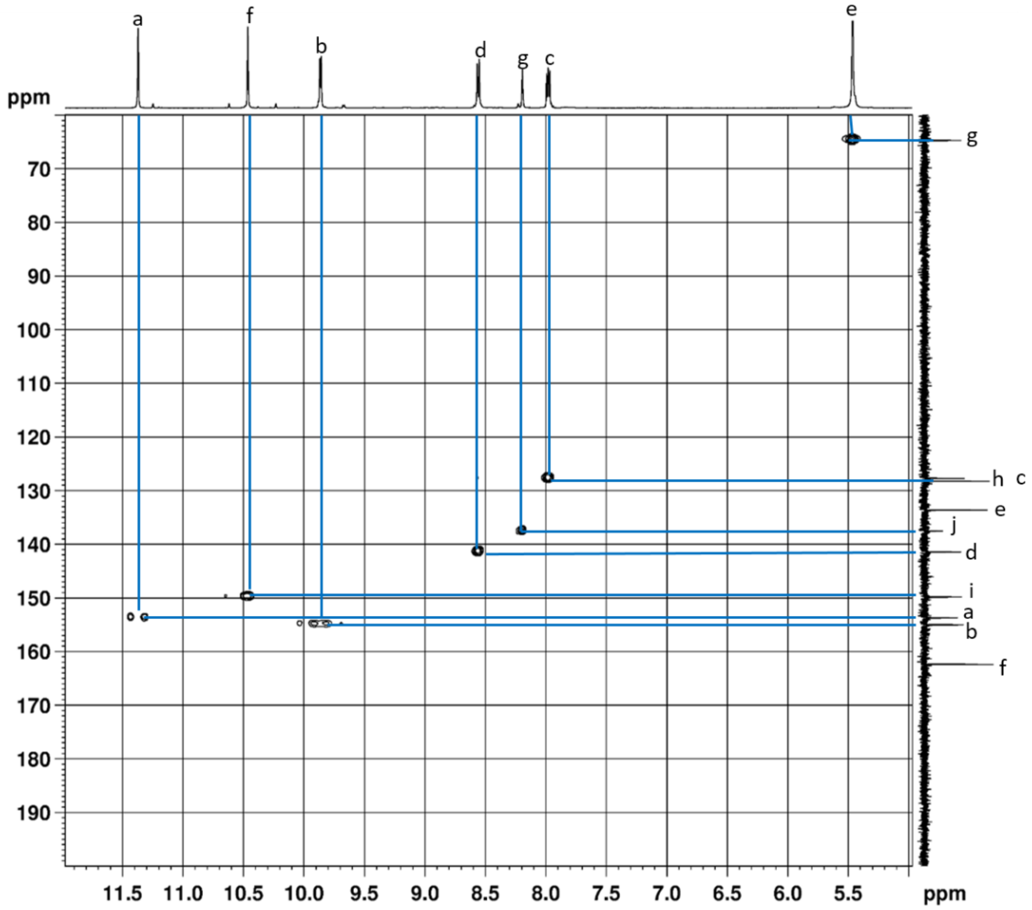

Figure S65b: C-H COSY expansion (500 MHz, DMSO-d_6, 300 K) for **7a**.

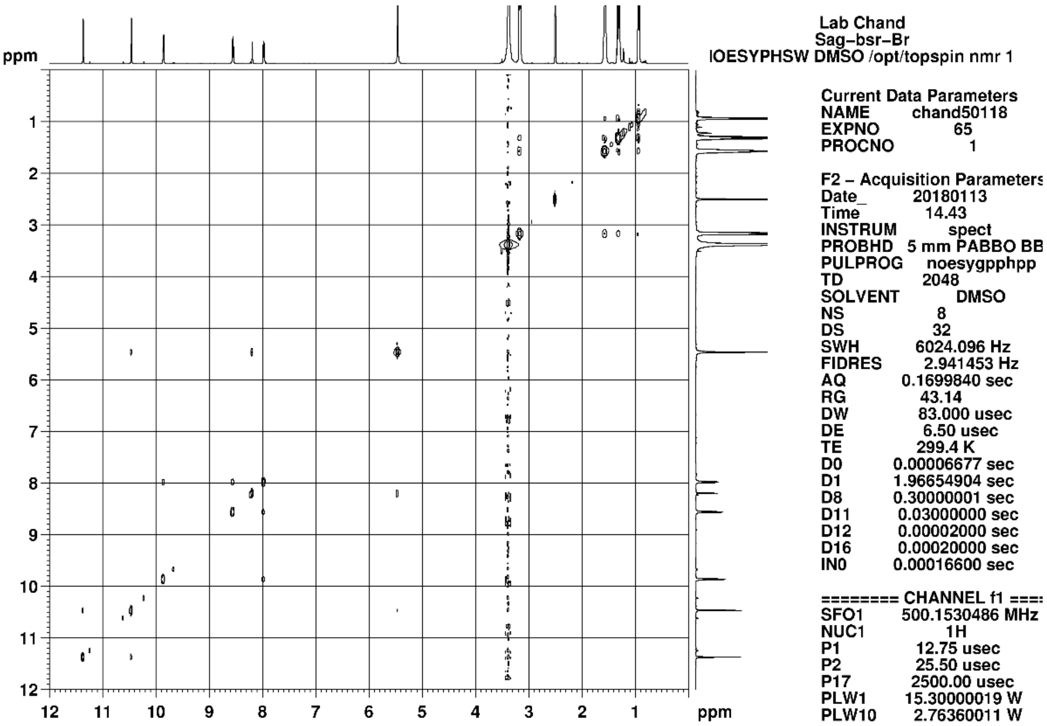

Figure S66a: NOESY (500 MHz, DMSO-d_6, 300 K) for **7a**.

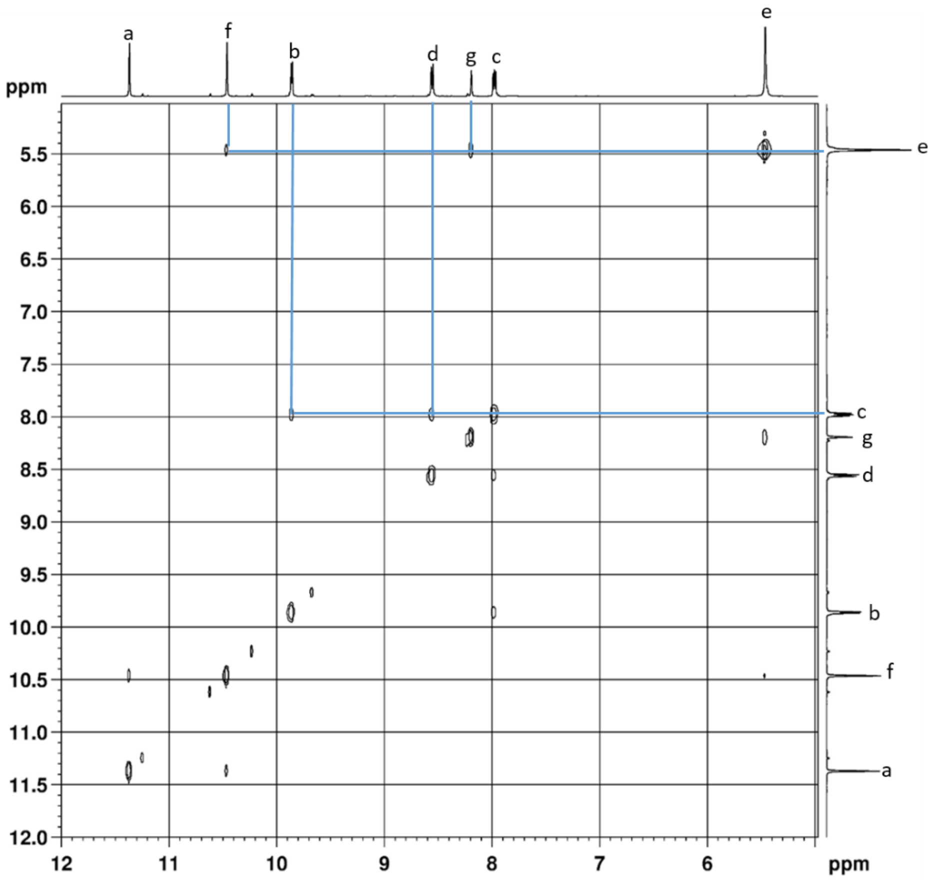

Figure S66b: NOESY expansion (500 MHz, DMSO-d_6, 300 K) for **7a**.

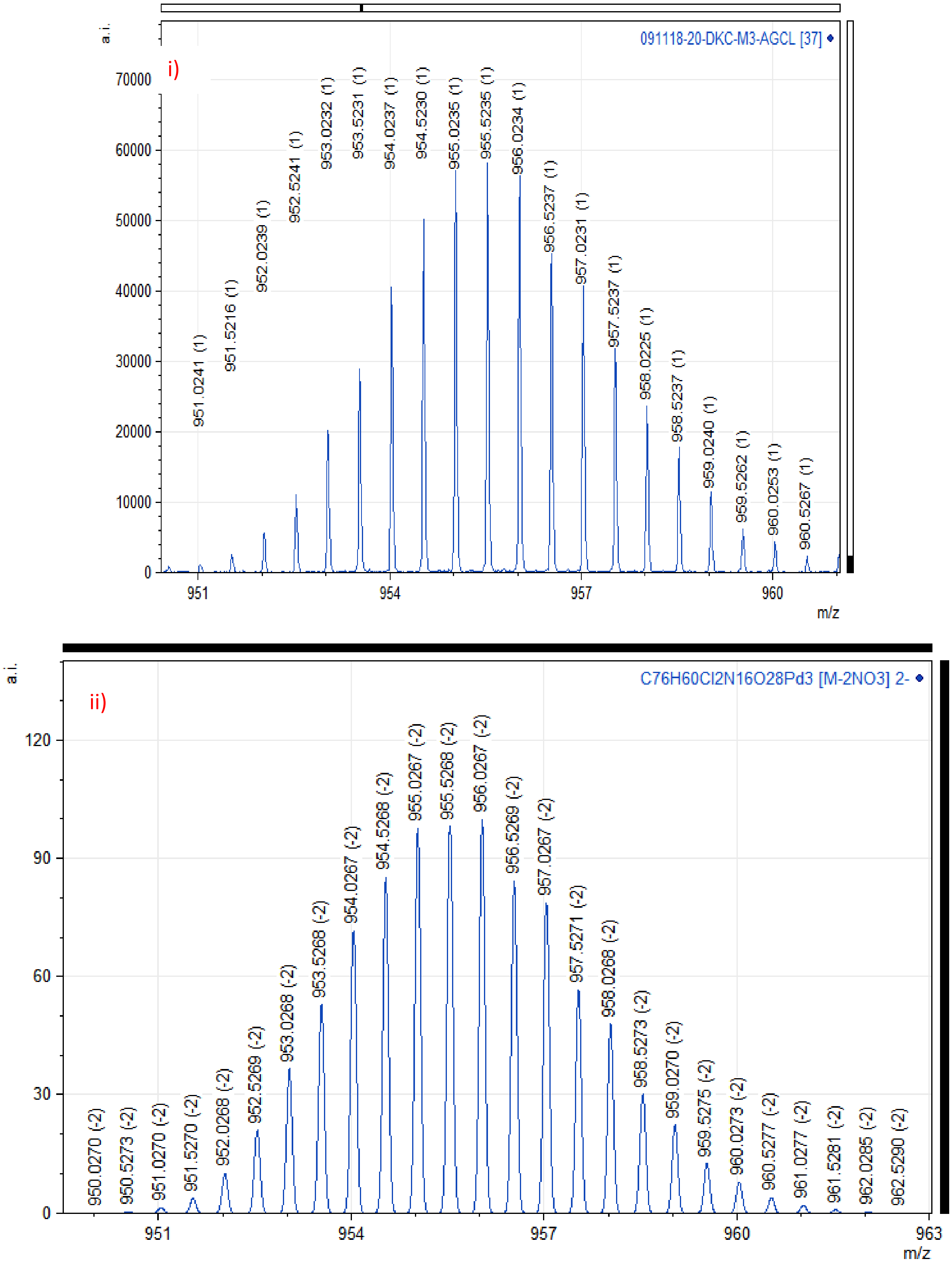

Figure S67: ESIMS, isotopic pattern for [**6a** − $2NO_3$]$^{2+}$ i) experimental and ii) theoretical.

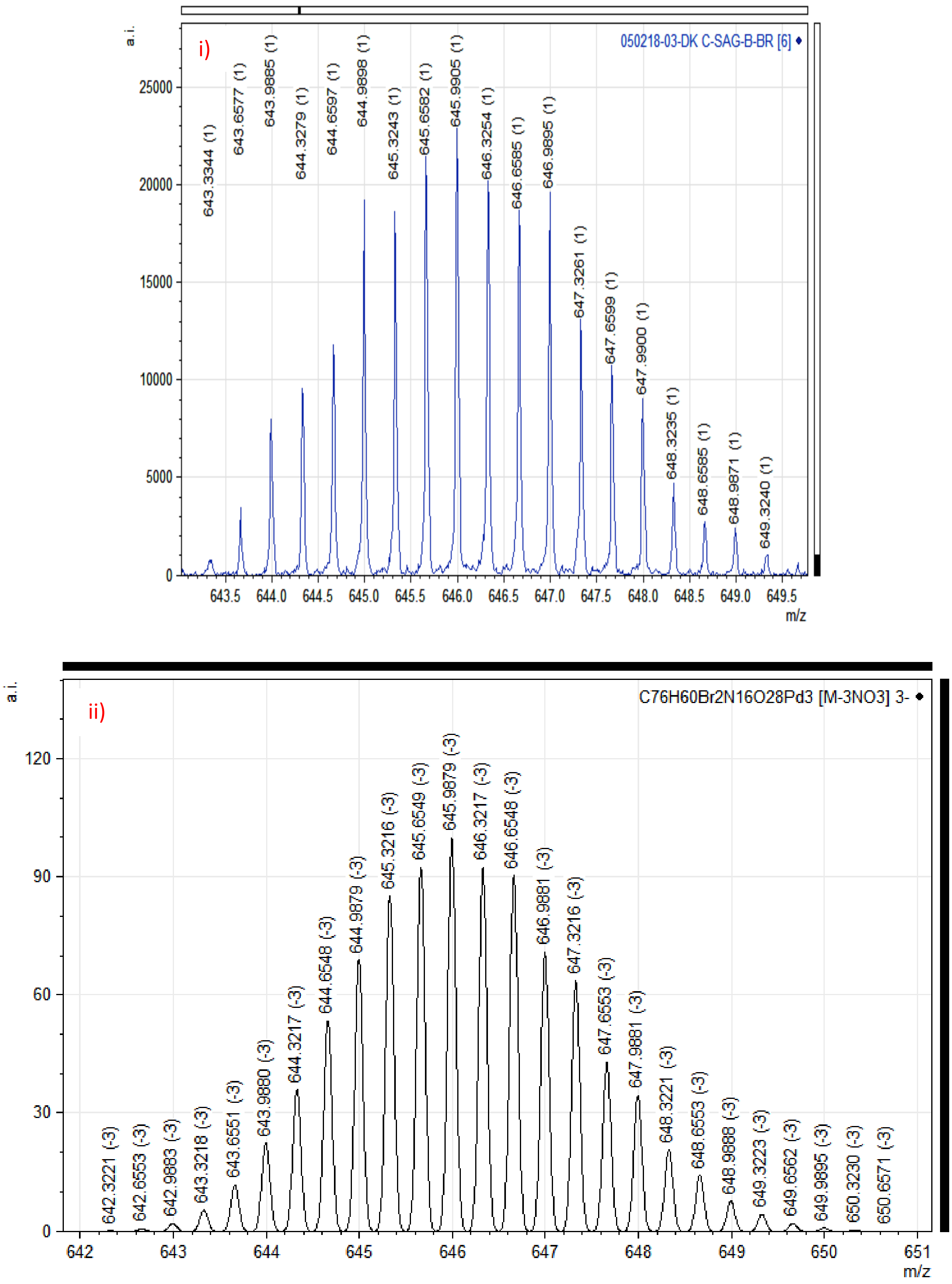

Figure S68a: ESIMS, isotopic pattern for [**7a** − $3NO_3$]$^{3+}$i) experimental and ii) theoretical.

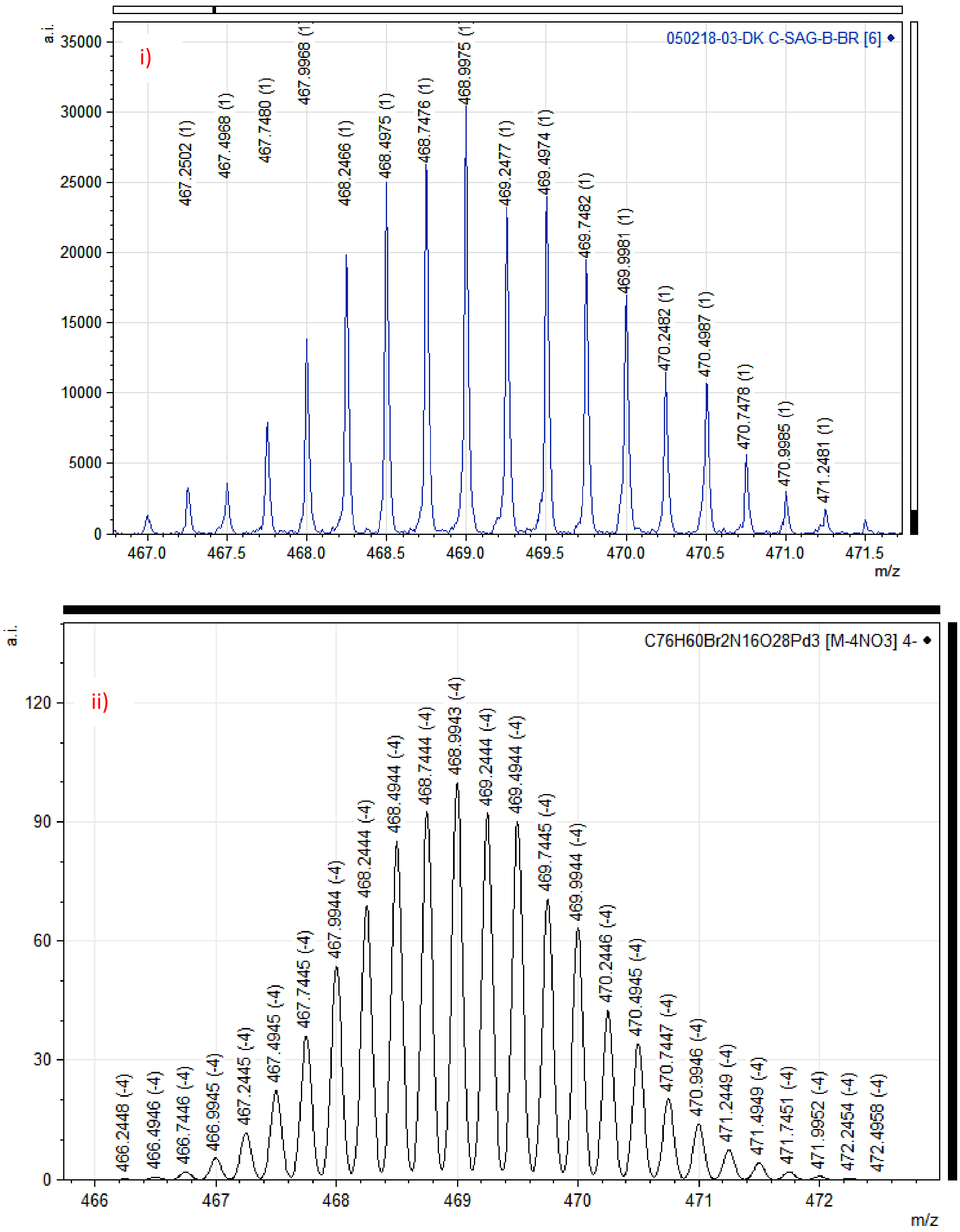

Figure S68b: ESIMS, isotopic pattern for [**7a** − $4NO_3$]$^{4+}$ i) experimental and ii) theoretical.

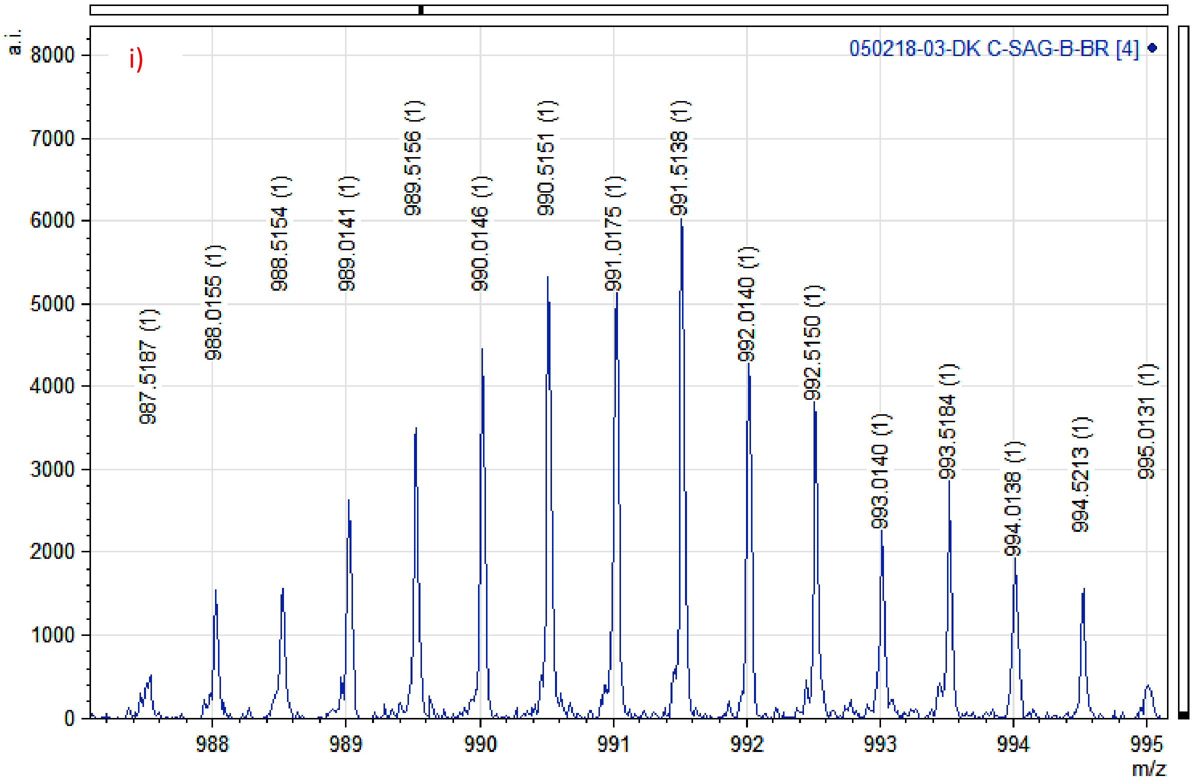

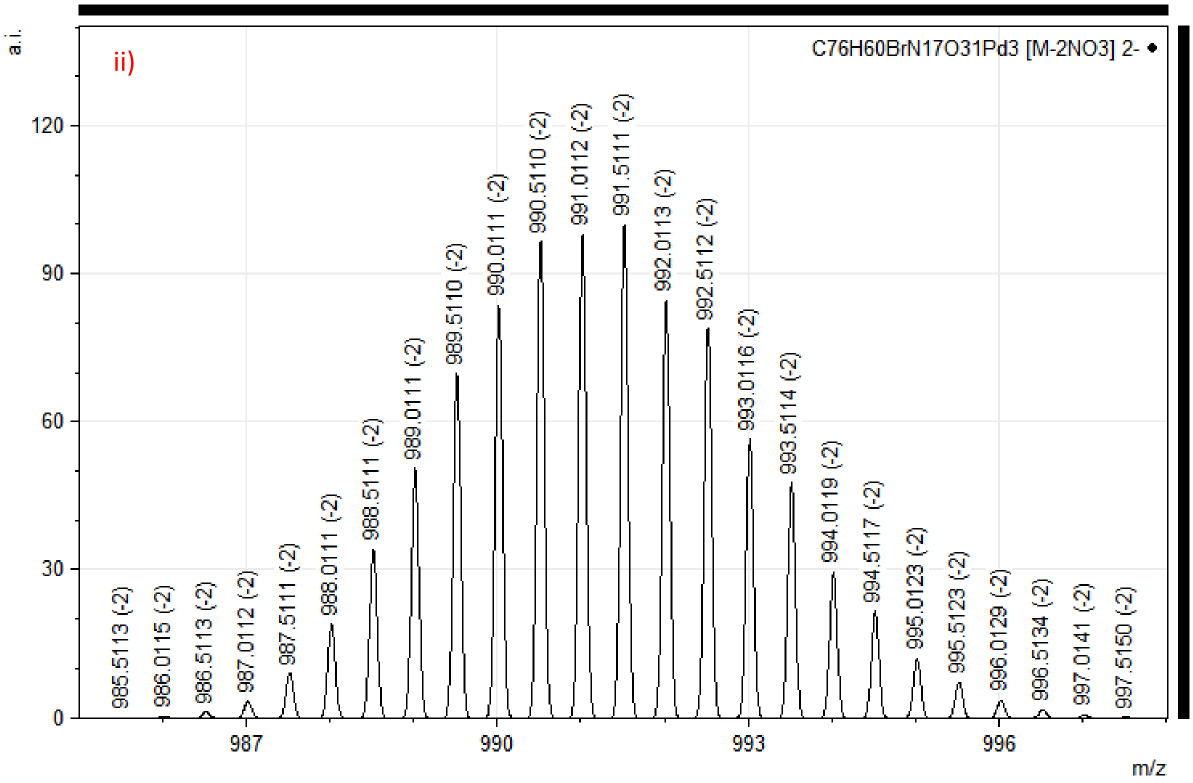

Figure S69: ESIMS, isotopic pattern for $[(NO_3)(Br)@Pd_3(\mathbf{L1})_4](NO_3)_4 - 2NO_3]^{2+}$, i.e., $[\mathbf{7a'} - 2NO_3]^{2+}$ i) experimental and ii) theoretical.

Table S1: ^{1}H NMR (DMSO-d_6) chemical shift of protons in ppm for the ligand **L1** and complexes **3a–7a**.

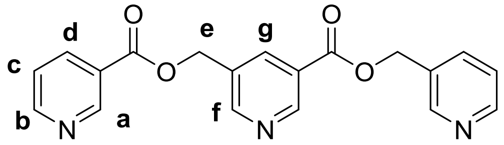

	L	Pd_1L_2 **3a**	$(NO_3)_2Pd_3L_4$ **4a**	$(F)_2Pd_3L_4$ **5a**	$(Cl)_2Pd_3L_4$ **6a**	$(Br)_2Pd_3L_4$ **7a**
H_a	9.13	9.92	10.55	10.88	11.21	11.37
H_b	8.83	9.30	9.65	9.82	9.84	9.85
H_c	7.57	7.82	7.95	7.98	7.99	7.98
H_d	8.33	8.54	8.57	8.58	8.57	8.55
H_e	5.47	5.63	5.45	5.44	5.46	5.46
H_f	8.72	8.75	9.74	10.05	10.41	10.46
H_g	8.09	8.31	8.26	8.21	8.17	8.19

Section S8: Figures S70, S71, Tables S2, S3) and explanatory note related to theoretical study. (Energy-minimized structures of various components and self-assembled compounds, calculated data and explanation)

DFT studies were performed with the Gaussian 09 software package. The B3LYP (Becke's three parameter hybrid functional using the LYP correlation) functional was used for geometry optimizations and frequencies with LANL2DZ for Pd atom, and the 6-31G* basis set for carbon, nitrogen, oxygen, chloride and hydrogen. Frequency calculations were performed for the optimized structures to confirm the absence of any imaginary frequencies.

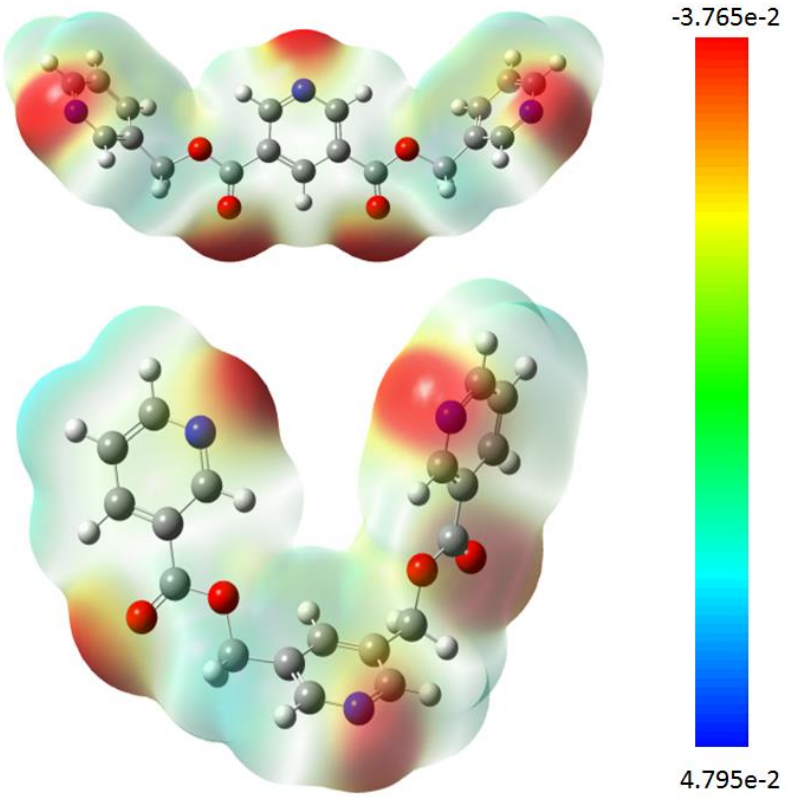

Figure S70. Energy minimization and electrostatic potential map of ligand **L1** (bottom) and ligand **L2** (bottom).

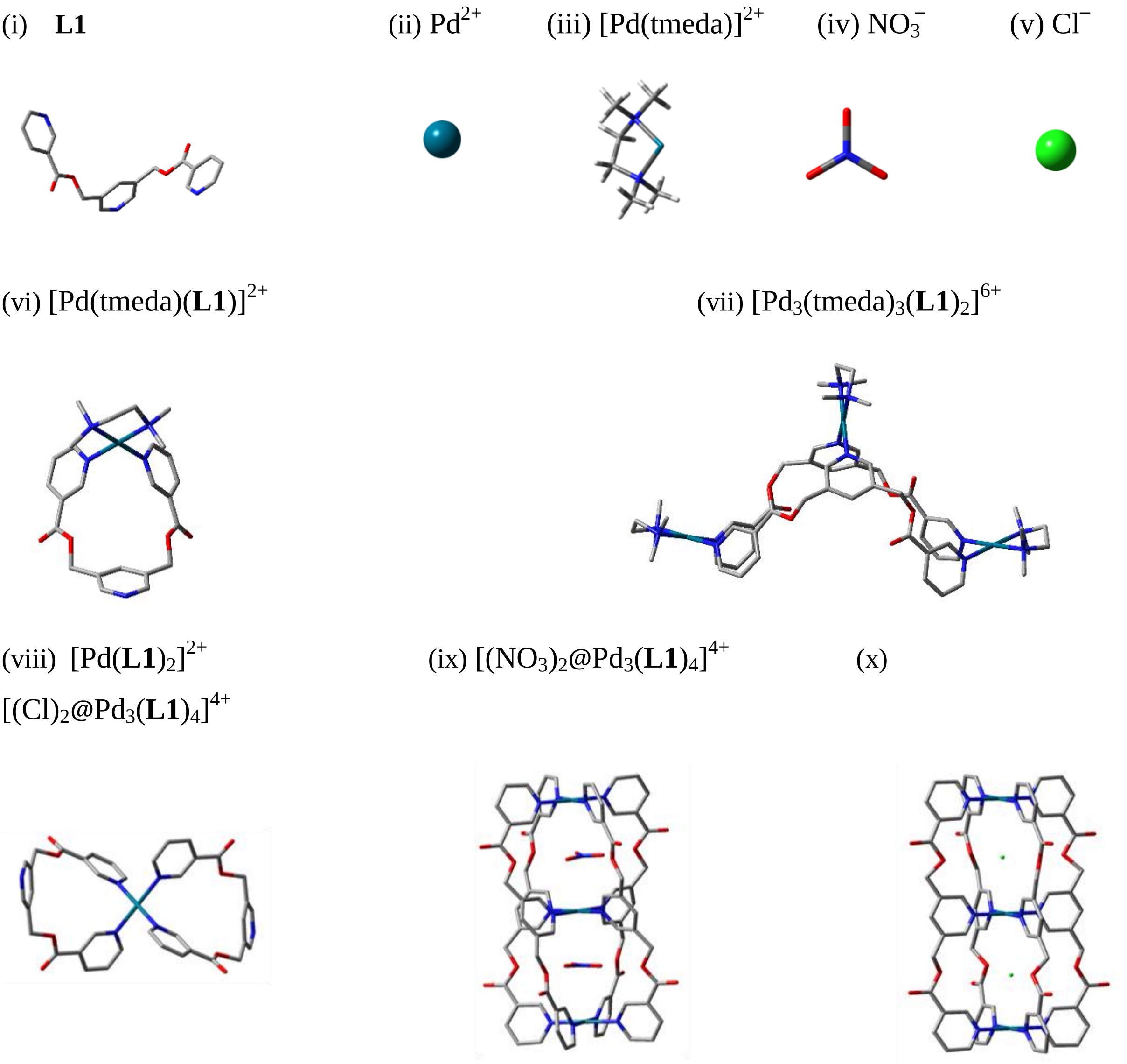

Figure S71: Energy-minimized structures of (i) ligand **L1**, (ii) Pd^{2+}, (iii) [Pd(tmeda)]$^{2+}$, (iv) NO_3^-, (v) Cl^-, (vi) [Pd(tmeda)(**L1**)]$^{2+}$, i.e., complexed cation of **1a**, (vii) [Pd_3(tmeda)$_3$(**L**)$_2$]$^{6+}$, i.e., complexed cation of **2a**, (viii) [Pd(**L**)$_2$]$^{2+}$, i.e., complexed cation of **3a**, (ix) [$(NO_3)_2$@Pd_3(**L**)$_4$]$^{4+}$, i.e., complexed cation (with encapsulated anion) of **4a**, and (x) [$(Cl)_2$@Pd_3(**L**)$_4$]$^{4+}$, i.e., complexed cation (with encapsulated anion) of **5a**.

Table S2. DFT calculated electron density at the terminal and internal pyridine nitrogen atoms of ligands, **L1** and **L2**.

Ligands	Electron density of terminal pyridine nitrogen atoms	Electron density of internal pyridine nitrogen atom
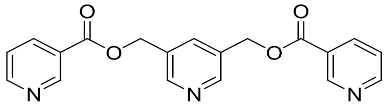 **L1**	-0.412 a.u.	-0.407 a.u.
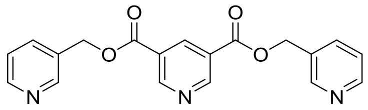 **L2**	-0.430 a.u.	-0.443 a.u.

Table S3: Δ*G* and Δ*H* values of (i) ligand **L1**, (ii) [Pd(DMSO)$_2$(NO$_3$)$_2$], (iii) [Pd(tmeda)(NO$_3$)$_2$] (iv) NO_3^-, (v) Cl$^-$, (vi) [Pd(tmeda)(**L**)]$^{2+}$, (vii) [Pd$_3$(tmeda)$_3$(**L**)$_2$]$^{6+}$, (viii) [Pd(**L**)$_2$]$^{2+}$, (ix) [(NO$_3$)$_2$@Pd$_3$(**L**)$_4$]$^{4+}$, and (x) [(Cl)$_2$@Pd$_3$(**L**)$_4$]$^{4+}$ (xi) DMSO.

Entry	Compounds	Enthalpy (in kcal mol^{-1})	Gibb's Free Energy (in kcal mol^{-1})
(i)	**L**	-751682.0595	-751733.5335
(ii)	[Pd(DMSO)$_2$(NO$_3$)$_2$]	-1125449.0769	-1125496.8523
(iii)	[Pd(tmeda)(NO$_3$)$_2$]	-649375.0234	-649418.72882
(iv)	NO_3^-	-175919.6833	-175901.7535
(v)	Cl^-	-288810.9376	-288821.8455
(vi)	[Pd(tmeda)(**L**)]$^{2+}$	-1048994.798	-1049057.135
(vii)	[Pd$_3$(tmeda)$_3$(**L**)$_2$]$^{6+}$	-2394987.525	-2395113.142
(viii)	[Pd(**L**)$_2$]$^{2+}$	-1582603.696	-1582685.713
(ix)	[(NO$_3$)$_2$ in Pd$_3$(**L**)$_4$]$^{4+}$	-3596488.124	-3596641.832
(x)	[(Cl)$_2$ in Pd$_3$(**L**)$_4$]$^{4+}$	-3822347.415	-3822512.255
(xi)	DMSO	-347075.8591	-347097.8013

Explanatory note1:

$$2\,[\mathrm{Pd(tmeda)}(\mathbf{L})]^{2+} + [\mathrm{Pd(tmeda)(NO_3)_2}] \longrightarrow [\mathrm{Pd_3(tmeda)_3}(\mathbf{L})_2]^{6+} + 2\mathrm{NO_3}^- \quad \text{-------- (Eq. 1)}$$

$$2\,[\mathrm{Pd}(\mathbf{L})_2]^{2+} + [\mathrm{Pd(DMSO)_2(NO_3)_2}] \longrightarrow [(\mathrm{NO_3})_2 \text{ in } \mathrm{Pd_3}(\mathbf{L})_4]^{4+} + 2\mathrm{DMSO} \quad \text{-------- (Eq. 2)}$$

$$[(\mathrm{NO_3})_2 \text{ in } \mathrm{Pd_3}(\mathbf{L})_4]^{4+} + 2\,\mathbf{L} \longrightarrow 3\,[\mathrm{Pd}(\mathbf{L})_2]^{2+} + 2\,\mathrm{NO_3}^- \quad \text{-------- (Eq. 3)}$$

$$[(\mathrm{NO_3})_2 \text{ in } \mathrm{Pd_3}(\mathbf{L})_4]^{4+} + 2\,\mathrm{Cl}^- \longrightarrow [(\mathrm{Cl})_2 \text{ in } \mathrm{Pd_3}(\mathbf{L})_4]^{4+} + 2\,\mathrm{NO_3}^- \quad \text{-------- (Eq. 4)}$$

(1) With reference to the equation 1: The overall free energy and the enthalpy for the formation of $[Pd_3(tmeda)_3(\mathbf{L})_2]^{6+}$ from 2 equiv of $[Pd(tmeda)(\mathbf{L})]^{2+}$ and 1 equiv of $[Pd(tmeda)(NO_3)_2]$ (equation 1) is 616.349 kcal mol^{-1} and 537.727 kcal mol^{-1}, respectively, which indicates that the reaction is non-feasible and endothermic. The global entropy (ΔS) value is −0.264 kcal mol^{-1} K^{-1} ($\Delta S = (\Delta H - \Delta G)/T$; T = 298.150 K) indicates that the reaction is non-spontaneous. This reaction did not undergo completion in reality, as per experimental data.

(2) With reference to the equation 2: The free energy and the enthalpy for the formation of $[(NO_3)_2@Pd_3(\mathbf{L})_4]^{4+}$ from 2 equiv of $[Pd(\mathbf{L})_2]^{2+}$, 2 equiv of $[Pd(DMSO)_2(NO_3)_2]$ (equation 2) is 30.843 kcal mol^{-1} and 16.627 kcal mol^{-1}, respectively, which indicates that the reaction is not feasible and endothermic. The global entropy (ΔS) value is −0.048 kcal mol^{-1} K^{-1} indicates spontaneous. This reaction actually proceeded in reality, as per experimental data.

(3) With reference to the equation 3: Furthermore, the free energy and the enthalpy for the formation of 3 equiv $[Pd(\mathbf{L})_2]^{2+}$, and 2 equiv of NO_3^- from $[(NO_3)_2@Pd_3(\mathbf{L})_4]^{4+}$ and 2 equiv **L** (equation 3) is 248.253 kcal mol^{-1} and −201.788 kcal mol^{-1}, respectively, which indicates that the reaction is non-feasible and exothermic. The global entropy (ΔS) −1.51 kcal mol^{-1} K^{-1} indicates spontaneous. However, this reaction actually proceeded in reality, as per experimental data.

(4) With reference to the equation 4: The free energy and the enthalpy for the formation of $[(Cl)_2@Pd_3(\mathbf{L})_4]^{4+}$ and 2 equiv of NO_3^- from $[(NO_3)_2@Pd_3(\mathbf{L})_4]^{4+}$ and 2 equiv Cl^- (equation 4) is −30.239 kcal mol^{-1} and 76.78 kcal mol^{-1}, respectively, which indicates that

the reaction is feasible and endothermic. The global entropy (ΔS) value is 0.359 kcal mol^{-1} K^{-1} indicates spontaneous. This reaction actually proceeded partially in reality, as per experimental data.

(5) With reference to the equation 2 and equation 3: These equations represent interconversion of mononuclear $[Pd(\mathbf{L})_2]^{2+}$ and trinuclear $[(NO_3)_2@Pd_3(\mathbf{L})_4]^{4+}$ under appropriate conditions. The conversion of the mono- to trinuclear complex is calculated to be endothermic. However, practically the reaction went to completion. On the other hand, the conversion of the tri- to mononuclear complex is calculated to be endothermic, probably due to a large energy barrier and difficult to occur. This reaction actually proceeded partially in reality, as per experimental data.
